# CONCERT predicts niche-aware perturbation responses in spatial transcriptomics

**DOI:** 10.1101/2025.11.08.686890

**Published:** 2025-11-20

**Authors:** Xiang Lin, Zhenglun Kong, Soumya Ghosh, Manolis Kellis, Marinka Zitnik

**Affiliations:** 1 Department of Biomedical Informatics, Harvard Medical School, Boston, MA, USA; 2 Merck & Co., Inc., Cambridge, MA, USA; 3 Kempner Institute for the Study of Natural and Artificial Intelligence, Harvard University, Cambridge, MA, USA; 4 Broad Institute of MIT and Harvard, Cambridge, MA, USA; 5 Harvard Data Science Initiative, Cambridge, MA, USA; 6 Computer Science and Artificial Intelligence Laboratory, MIT, Cambridge, MA, USA

## Abstract

Spatial perturbation transcriptomics measures how genetic or chemical edits alter gene expression while preserving tissue context. Perturbation outcomes depend on a cell’s intrinsic state and also on how effects propagate across cellular microenvironments. We present CONCERT, a niche-aware generative model that embeds perturbation context and learns spatial kernels with a Gaussian process variational autoencoder to predict perturbation effects across tissue. We formalize three tasks: patch, border, and niche, predicting responses in nearby unperturbed regions, at tissue interfaces, and as a function of surrounding microenvironments. We evaluate CONCERT on Perturb-map lung datasets. CONCERT outperforms state-of-the-art models (dissociated counterfactuals, spatialized perturbation models, and kNN), reducing E-distance by up to 33.77% (patch), 26.05% (border), and 33.74% (niche) versus the next best, with mean absolute error down by up to 23.28% and Pearson correlation up by up to 9.10%. Two case studies go beyond benchmarking. In dextran sodium sulfate-induced colitis, CONCERT reconstructs spatial gene expression at unmeasured time points, produces longitudinal comparisons across unpaired mice, resolves inter-mouse heterogeneity, and recovers consistent temporal declines of inflammation-associated genes across regions. In ischemic stroke, CONCERT predicts responses under variable lesion sizes and in a 3D formulation across brain sections, capturing lesion-core and peri-lesion patterns. CONCERT performs niche-aware counterfactual prediction, reconstructs missing spatial data, and models perturbation responses across tissues.

Cellular responses to perturbations depend on spatial context and intrinsic factors [1]. The cell niche, defined by neighboring cells and the local microenvironment, shapes transcriptional outcomes by controlling exposure to signaling molecules [2], opportunities for communication [3], access to nutrients [4], and immune surveillance [5]. In immunotherapy, the niche shapes the response to treatment by controlling the composition, activation and expression of immune cells of checkpoint molecules such as PD-L1 [6-8]. Analyses that ignore spatial organization can miss critical biology in development [9], immunity [10], and disease [11].

In spatial transcriptomics, perturbations are introduced *in vivo* while preserving tissue context and intercellular interactions (Supplementary Fig. 2) [12-14]. This approach measures how perturbation effects spread across neighboring cells and niches, which dissociated-cell data cannot capture. Sequencing is destructive, so the same cell cannot be profiled before and after perturbation. Counterfactual prediction is therefore central: models must infer gene expression under conditions that are not directly observed or experimentally feasible.

Single-cell transcriptomics models pretrain on large cell atlases to learn reusable cell and gene representations [15-18]. These models use dissociated scRNA-seq and exclude spatial coordinates and neighborhood graphs, so they lack spatial context and cannot operate on spatial transcriptomics. Perturbation models such as scGEN [19], CPA [20], CellOT [21], GEARS [22], BioLORD [23], and PDGrapher [24] predict responses but treat each cell in isolation, despite evidence that niches shape gene expression [1]. In parallel, spatial transcriptomics methods address deconvolution and spatial patterning [25-33] yet do not analyze responses to perturbations.

Spatial perturbation methods begin to address these challenges. Vespucci [34], River [35], and Perturb-STNet [36] identify regions, genes, programs, or regulators that respond to observed perturbations, but they cannot generate counterfactual predictions for unobserved perturbations or their spatial effects (limitation 1). Celcomen [37] disentangles intra- and inter-cellular regulation and infers perturbation effects from learned gene-gene interactions, but it uses a single global interaction matrix and assumes spatially invariant regulation. This design misses niche-dependent propagation of perturbation effects and leads to inaccurate spatial modeling (limitation 2). stFormer [38], Morpheus [39], and CellAgentChat [40] perform *in silico* perturbations. stFormer models ligand-receptor interactions with a transformer, Morpheus applies counterfactual optimization on spatial proteomic profiles to enhance T-cell infiltration, and CellAgentChat simulates perturbations using an agent-based framework. Each is narrow in scope: stFormer is confined to ligand-receptor interactions, Morpheus to proteomic data and a single immune phenotype, and CellAgentChat to rule-based dynamics (limitation 3).

We introduce CONCERT, a generative model for spatial perturbation prediction (Fig. 1a). CONCERT has three modules: a perturbation module that disentangles perturbation identity in latent space and applies *in silico* perturbation at the target sport (addressing limitation 1), a spatial module that models spatial and cell-specific propagation of effects (addressing limitation 2), and a generative module that reconstructs response gene expression (rGEX) under this perturbation (Fig. 1bcd). CONCERT handles multiple perturbation types and learns their spatial effects across 2D and 3D tissue contexts (addressing limitation 3). Given an unperturbed cell’s gene expression profile, spatial coordinates, and perturbation identity, CONCERT predicts the cell’s rGEX, accounting for information from the cell’s surrounding microenvironment. CONCERT can also impute missing cells on tissue slides and predict their responses, providing expression profiles in regions that lack measurements due to sparse sampling or tissue damage.

We formalize three tasks in spatial perturbation transcriptomics: patch, border, and niche (Extended Data Fig. 1 and Supplementary Note 3) and benchmark CONCERT on Perturb-map, a dataset of Visium lung cancer tissues subjected to single or double CRISPR knockouts [12]. Across all three tasks, CONCERT outperforms seven competing models, lowering E-distance by up to 33.77% (patch), 26.05% (border), and 33.74% (niche) and producing well-calibrated uncertainties (95% coverage). We next evaluate imputation on a mouse colon inflammation dataset [41]. CONCERT reconstructs unmeasured spatial gene expression profiles, generates longitudinal comparisons across mice sampled at different time points, and reveals inflammation-recovery trends for colon regions that were obscured by inter-mouse variability in the dataset. We also use CONCERT for 2D and 3D perturbation prediction using a mouse brain dataset with photothrombosis-induced stroke [42]. CONCERT predicts rGEX at locations and lesion sizes that cannot be experimentally assayed, and its predictions align with spatial patterns observed in stroke. Benchmarking on lung cancer tissues, reconstruction of missing time points in colitis, and 2D/3D lesion-size prediction in stroke show that CONCERT performs niche-aware prediction across tissues and conditions.

## Results

### Generative CONCERT model for spatial perturbations

**Figure 1:**
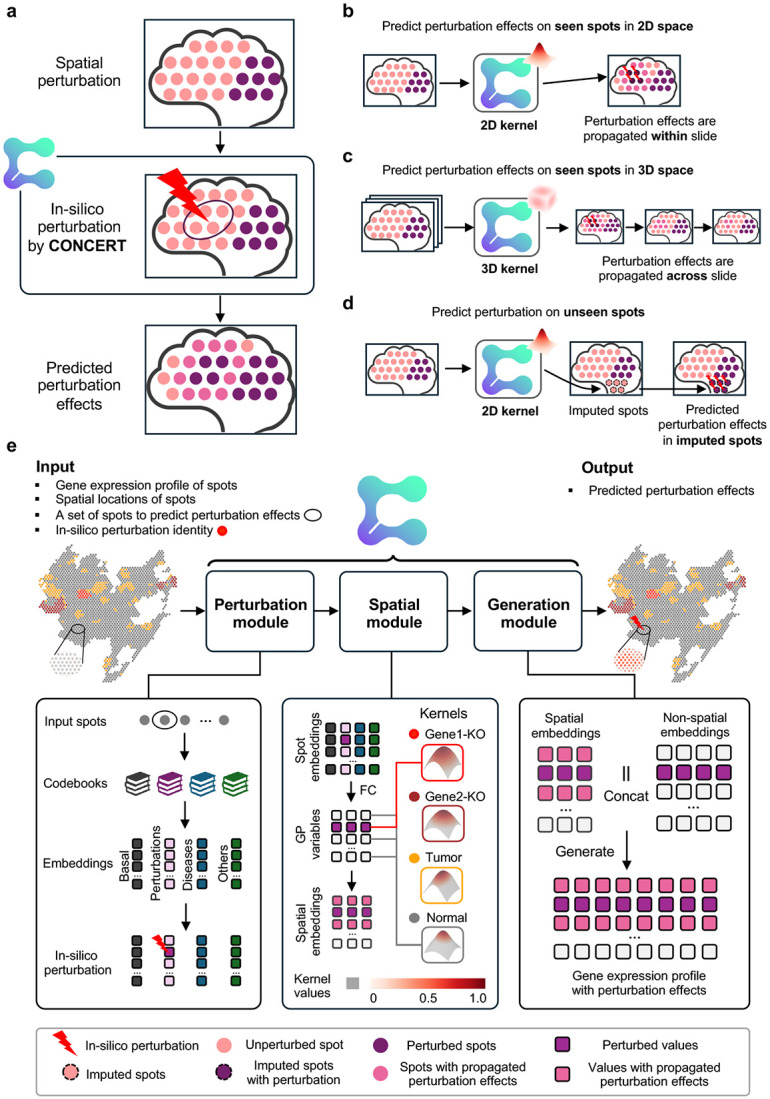
Overview of CONCERT. **a.** CONCERT is a model to predict spatial effects of perturbations. **b, c.** CONCERT addresses this gap by modeling how perturbation effects propagate in space. A 2D kernel captures dispersion within a single tissue section (b), and a 3D kernel extends predictions across consecutive sections (c). **d.** Beyond observed data, CONCERT can impute missing spots and predict how perturbations affect these reconstructed regions, enabling *in silico* experiments even when tissue is sparse or damaged. **e.** CONCERT has three modules: a perturbation module that encodes perturbation identity, a spatial module that learns Gaussian process (GP) kernels to capture how effects of perturbations propagate across tissue, and a generation module that predicts response gene expression (rGEX). Given an unperturbed cell’s gene expression profile, spatial coordinates, and perturbation identity, CONCERT predicts the cell’s rGEX, accounting for information from the cell’s surrounding microenvironment. FC stands for a fully connected neural network.

**Figure 2:**
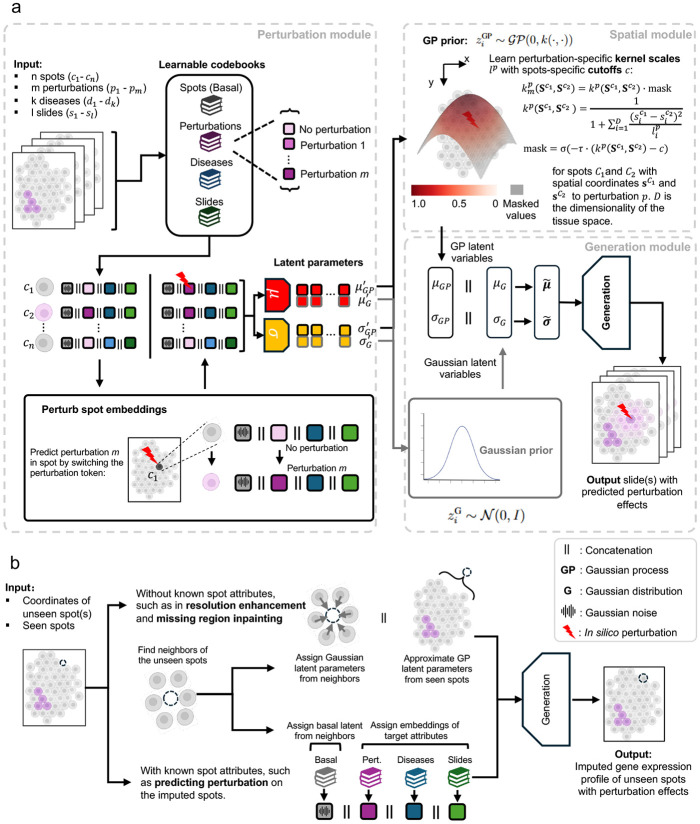
Architecture of CONCERT. CONCERT can be applied to both cell- and spot-level data; for clarity, each data point is referred to as a spot. **a.** Input slides of spatial transcriptomics data are first processed through learnable codebooks that generate embeddings disentangling perturbation state and other attributes. Perturbations are then introduced by modifying these embeddings. The perturbed embeddings are passed through a Gaussian Process (GP) latent space and a Gaussian latent space, producing posterior variables $\mu_{\text{GP}}∕\sigma_{\text{GP}}$ (with spatial dependencies) and $\mu_{G}∕\sigma_{G}$ (without spatial dependencies). These are concatenated and decoded to predict post-perturbation gene expression. **b.** CONCERT can also impute missing spots, either with or without known attributes, and then apply *in silico* perturbations. This allows predictions in regions where expression was not observed experimentally.

CONCERT predicts cellular response gene expression (rGEX) to perturbations while accounting for the surrounding microenvironment (Fig. 1a). The model has three modules: (1) a perturbation module, (2) a spatial module, and (3) a generation module (Fig. 2). It performs counterfactual prediction with both categorical and continuous attributes. Perturbations are encoded in the perturbation module, propagated through the spatial module, and used by the generation module to produce rGEX (Fig. 2a).

The perturbation module encodes perturbation identity, such as perturbagens A, B, and C or control, and their attributes such as dose or time. It generates counterfactuals in three ways: swapping perturbation identity (e.g., control to perturbagen A), relocating or resizing perturbed patches of cells (e.g., moving a tumor patch to a different niche), or adjusting perturbation attributes (e.g., extending post-perturbation time).

The spatial module propagates encoded perturbations through tissue using a kernel with learnable scales and masks. This kernel models anisotropic, non-local propagation while respecting tissue boundaries and niches, producing perturbation fields with directional effects and nonsmooth decay (Fig. 1e and Supplementary Fig. 32).

The generation module combines latent representations with and without perturbation fields to predict rGEX at both measured and unmeasured locations. CONCERT uses a variational encoder to map observed gene expression into latent states and a conditional decoder to generate rGEX conditioned on those states and perturbation fields. By varying the number of *in silico* perturbed cells, the model extrapolates perturbation effects for unmeasured lesion sizes. By modifying encoded attributes, it extrapolates effects for unseen times or doses. Additional attributes such as tissue or disease can also be incorporated. Evaluating CONCERT at new coordinates, with or without perturbations, predicts expression at higher resolution and reconstructs damaged or missing regions with *in silico* perturbations.

### A benchmark for prediction of spatial perturbation responses

We benchmarked CONCERT on Perturb-map spatial perturbation datasets that preserve spot positions while measuring gene expression after perturbation [43]. The data include four mouse lung sections with tumor spots profiled on the 10× Visium platform. Perturbation labels were obtained from the original study using imaging mass cytometry (Supplementary Fig. 4, Supplementary Fig. 5) and were performed *in vivo*, so the exact locations of perturbed spots cannot be controlled (Supplementary Fig. 1). We used CONCERT to predict perturbation effects in unperturbed spots and analyze predicted responses in the tumor microenvironment. It is noted that CONCERT can be applied to both cell- and spot-level data. Because all spatial perturbation datasets used in this study are spot-level, each data entry is referred to as a spot in this section.

We defined three tasks for evaluation (Supplementary Note 3). We use the following terms: *perturbed spots* are those that undergo *in silico* perturbation; *predicted spots* are those for which rGEX is predicted and evaluated; *source state* and *target state* are gene expression states before and after perturbation (e.g., tumor vs. Jak2-KO tumor). Based on these definitions: (i) Within- and cross-niche prediction: a patch *X* of source state *A* is perturbed to state *B* observed in nearby (same niche) or distant (different niche) spots; predictions are made for *X*. (ii) Border prediction: spots *X* of state *A* on a tissue boundary are perturbed to *B*; predictions are made for *X*. (iii) Niche prediction: a patch is divided into border spots *X* and core spots *Y ; X* is perturbed from *A* to *B*, and predictions are made for *Y*. Task difficulty increases with the distance and heterogeneity over which effects must propagate. Within-niche prediction is the easiest, border prediction is harder because of sharp tissue interfaces, and cross-niche and niche prediction are the most challenging because models must extrapolate across niches and capture non-local dispersion. Performance is reported using energy distance (E-distance), mean absolute error (MAE), Pearson correlation coefficient (PCC), and $R^{2}$.

We benchmarked CONCERT against three groups of models. (i) kNN-based methods (kNN-SP and kNN-GEX) predict from nearest neighbors in spatial or gene expression space, respectively; because evaluation also leverages similarity in observed space, kNN-SP serves as an approximate upper bound [44]. (ii) Counterfactual prediction methods trained on dissociated data: scGEN [19], BioLORD [23], and CPA [20]. (iii) Spatialized models, created by replacing the multilayer perceptrons in scGEN, BioLORD, and CPA with graph convolutional layers to incorporate adjacency information. Predictions were compared to the ground truth spots in Perturb-map using the gene expression of the spots with the target state and following the established approach in benchmarking perturbation models [19, 20, 44]. We tested robustness by repeating the analyses 1,000 times while sampling 1, 4, and 8 spots.

### Benchmarking CONCERT on within-niche perturbation tasks

**Figure 3:**
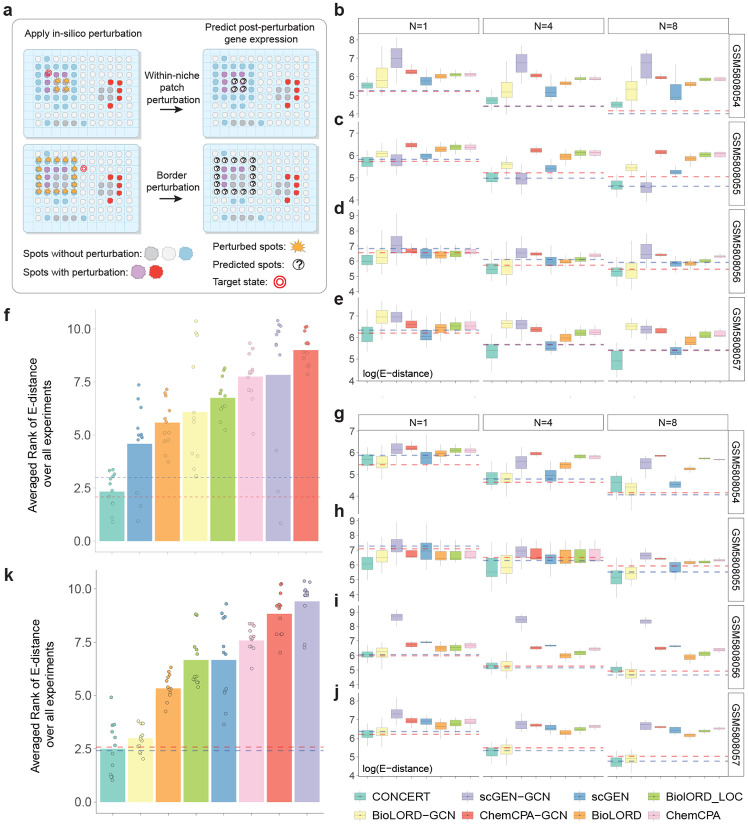
Performance of CONCERT on patch and border prediction tasks. **a, top.** Patch task schematic. A cluster of unperturbed spots (left) is perturbed into a target state carried by nearby spots in the same niche (right). This tests whether models can transfer perturbation effects within a tissue region. **b-e.** Log-transformed E-distance for sub-patches of size 1, 4, and 8 (sampled 1000 times). CONCERT achieves the lowest error in most conditions, approaching or surpassing the kNN-SP upper bound (red dashed line). **f.** Averaged rank of E-distance across four samples and three patch sizes. CONCERT achieves the top overall score, outperforming all competing models. **a, bottom.** Border task schematic. Spots located along the edge of a tissue region are perturbed into a target state, and predictions are evaluated at the boundary. This tests whether models can capture perturbation effects across sharp tissue interfaces. **g-j.** Log-transformed E-distance for border lengths of 1, 4, and 8 spots (sampled 1000 times). CONCERT consistently outperforms competing methods, and in some samples even exceeds the kNN-SP upper bound. **k.** Averaged rank of E-distance across all border conditions. CONCERT ranks best overall, confirming robust performance at tissue boundaries. All boxplots show the median (center line), first and third quartiles (box edges), and whiskers extending 1.5× the interquartile range. P-values for comparisons between CONCERT and competing methods are in Supplementary Tables 1 and 4.

We benchmarked CONCERT on two within-niche perturbation tasks: patch prediction and border prediction (Fig. 3a).

In the patch task, the goal is to predict responses in a cluster of unperturbed spots when nearby spots of the same tissue niche carry the perturbation (Fig. 3a top and Extended Data Fig. 2a). This setting tests whether a model can transfer perturbation effects from one local region to a neighboring region of the same type. Across 3 patch sizes × 4 samples (12 conditions), CONCERT achieves the best performance in 9 (Fig. 3b-f). In samples GEM5808054, GEM5808055, and GEM5808056, it attains the lowest mean E-distances of 153.78, 196.17, and 436.759 (values are log-transformed in the figures). Relative to the second-best method, CONCERT yields 19.98% (*p* = 6.97 × 10^−6^), 33.54% (*p* = 1.70 × 10^−8^), and 33.77% (*p* = 8.48 × 10^−11^) lower E-distances for patch sizes 1, 4, and 8 of sample GEM5808054; 19.94% (*p* = 1.0 × 10^−4^) and 16.5% (*p* = 2.42 × 10^−4^) lower E-distances for patch sizes 1 and 4 of sample GEM5808056; and 10.11% (*p* = 3.84 × 10^−4^) and 33.26% (*p* = 1.72 × 10^−12^) lower E-distances for patch sizes 4 and 8 of sample GEM5808057. CONCERT approaches the kNN-SP upper bound in samples GEM5808054 and GEM5808055 and outperforms it in samples GEM5808056 and GEM5808057 (Fig. 3b-e). In the average-rank analysis across four samples and three patch sizes, CONCERT achieves the top mean rank score of 2.33, outperforming kNN-GEX (3.0) and closely approaching kNN-SP (2.08) (Fig. 3f). These results are supported by additional metrics (Supplementary Fig. 6, Supplementary Fig. 7, Supplementary Fig. 8, Supplementary Fig. 9, Supplementary Fig. 10, Supplementary Fig. 11).

In the border task, the goal is to predict responses for spots at the edge of tissue regions, where perturbation effects must propagate across sharp boundaries (Fig. 3a bottom and Extended Data Fig. 2b). This setting is harder than the patch task because border spots sit at interfaces between distinct tissue compartments. Across 3 patch sizes × 4 samples (12 conditions), CONCERT achieves the best performance in 8 (Fig. 3g-k). In samples GEM5808055, GEM5808056, and GEM5808057, it attains the lowest mean E-distances of 577.34, 267.42, and 279.48, even outperforming the estimated upper bound in sample GEM5808055 (Fig. 3g-j). Relative to the second-best method, CONCERT yields 17.39% (*p* = 2.42 × 10^−4^) lower E-distance in patch size 1 of sample GEM5808056, 26.05% (*p* = 4.0 × 10^−2^) lower E-distance in patch size 8 of sample GEM5808055, and 13.67% (*p* = 5.82 × 10^−4^), 15.71% (*p* = 3.29 × 10^−5^), and 17.88% (*p* = 1.35 × 10^−4^) lower E-distances in patch sizes 1, 4, and 8 of sample GEM5808057. In the average-rank analysis (Fig. 3k), CONCERT ranks highest with a score of 2.50, closely approaching the upper bound (2.41). These results are also consistent across other evaluation metrics (Supplementary Fig. 12, Supplementary Fig. 13, Supplementary Fig. 14, Supplementary Fig. 15, Supplementary Fig. 16, Supplementary Fig. 17).

### Benchmarking CONCERT on cross-niche perturbation tasks

**Figure 4:**
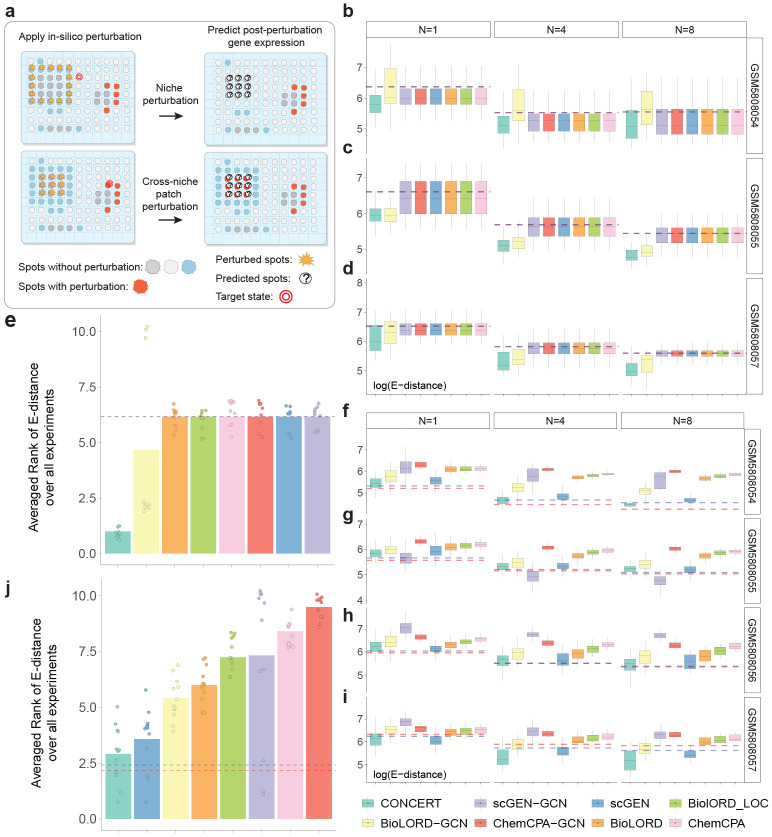
Performance of CONCERT on niche and cross-niche prediction tasks. **a, top.** Niche task schematic. Spots at the border of a patch are perturbed into a target state, and predictions are made for the unperturbed core of the patch. This tests whether models can capture how surrounding microenvironments shape responses in the center. **b-d.** Log-transformed E-distance for patches of size 1, 4, and 8 (sampled 1,000 times). Only CONCERT and bioLORD-GCN can perform this task; other methods collapse to trivial predictions. Across conditions, CONCERT achieves the lowest error. **e.** Averaged rank of E-distance across all niche tests. CONCERT consistently ranks first, showing it best captures microenvironmental influence. **a, bottom.** Cross-niche task schematic. A patch of spots in a source state is perturbed toward a target state observed only in a different niche. This requires extrapolation across regions with different cellular composition. **f-i.** Log-transformed E-distance for patch sizes of 1, 4, and 8 (sampled 1,000 times). CONCERT achieves the lowest error in most settings, substantially outperforming competing methods. **j.** Averaged rank of E-distance across all cross-niche conditions. CONCERT ranks best overall, confirming its robustness in extrapolating perturbation effects across distinct niches. Red and blue dashed lines indicate kNN baselines using spatial or gene-expression neighbors. Because evaluation also leverages similarity in observed space, kNN-SP serves as an approximate upper bound. Boxplots show the median (center line), first and third quartiles (box edges), and whiskers extending 1.5× the interquartile range. P-values for comparisons between CONCERT and competing methods are reported in Supplementary Tables 7 and 10.

We also evaluated CONCERT on two cross-niche prediction tasks. The first is a niche perturbation task, which tests whether a model can predict how perturbations applied to the surrounding microenvironment affect the spots in the center of a patch (Fig. 4a top and Extended Data Fig. 2c). This task assesses whether a cell’s response is influenced by the responses of its neighbors. Only CONCERT and bioLORD-GCN completed the task successfully; other methods simply reproduced the input GEX, yielding uninformative predictions. Across all nine conditions, CONCERT achieves the best performance. In samples GEM5808054, GEM5808055, and GEM5808057, it attains the lowest mean E-distances of 331.17, 242.18, and 302.70, respectively. Relative to the second-best method, on GEM5808055, CONCERT yields 9.56% (*p* = 2.57 × 10^−3^) and 14.34% (*p* = 1.73 × 10^−7^) lower E-distances when perturbed patches consist of 4 and 8 spots. On GEM5808057, CONCERT achieves 27.33% (*p* = 2.59 × 10^−3^), 18.53% (*p* = 1.59 × 10^−2^), and 33.74% (*p* = 1.42 × 10^−2^) lower E-distances than the second-best method for patches with 1, 4, and 8 spots (Fig. 4b-d). Averaged across samples, CONCERT attains the best average rank score of 1.0, outperforming bioLORD-GCN (Fig. 4e). Results are consistent across other performance metrics (Supplementary Fig. 18, Supplementary Fig. 19, Supplementary Fig. 20, Supplementary Fig. 21, Supplementary Fig. 22, Supplementary Fig. 23).

We also considered cross-niche prediction, the most challenging task. In this setting, spots in a source state are perturbed toward a target state that exists only in a different tissue niche (Fig. 4a bottom and Extended Data Fig. 2d). The task requires extrapolation across niches with distinct cellular compositions, which tests whether models can generalize beyond local neighborhoods. Across three patch sizes and four samples (12 conditions total), CONCERT achieves the best performance in seven conditions. For GSM5808054, GSM5808056, and GSM5808057, CONCERT attains the lowest mean E-distances of 143.85, 357.98, and 305.21, respectively. Relative to the second-best method, CONCERT yields 15.83% (*p* = 2.65 × 10^−4^), 18.53% (*p* = 1.56 × 10^−9^), and 18.63% (*p* = 3.81 × 10^−26^) lower E-distances for patches of 1, 4, and 8 spots in sample GEM5808054; 6.18% (*p* = 1.86 × 10^−2^) lower E-distance for 8-spot patches in sample GEM5808056; and 23.99% (*p* = 3.97 × 10^−4^) and 16.92% (*p* = 5.03 × 10^−4^) lower E-distances for 4- and 8-spot patches in sample GEM5808057 (Fig. 4f-i). Averaged across all conditions, CONCERT achieves the average rank score of 2.91 (Fig. 4j). Performance gains also hold across other performance metrics (Supplementary Fig. 24, Supplementary Fig. 25, Supplementary Fig. 26, Supplementary Fig. 27, Supplementary Fig. 28, Supplementary Fig. 29).

Finally, we evaluated predictive uncertainty. Coverage is well calibrated: 95% of values fall within the model’s 95% interval (Supplementary Fig. 30). Sensitivity analyses confirmed that CONCERT maintained robust performance across hyperparameters and training sample sizes (Supplementary Fig. 49 and Supplementary Fig. 50; Supplementary Note 2).

### When and why spatial kernels beat neighbor copying

kNN-based methods can fit local, low-variation settings where nearby neighbors share the same state and boundaries are weak. In tasks with distant states, niche interactions, confounding, or cross-slide transfer, their reliance on local copying limits performance. We designed four tests that expose these failure modes and show how CONCERT overcomes them (Extended Data Fig. 3). For convenience, we use NEX to denote normalized gene expression.

#### Distant source and target states.

When the source and target states are far apart, kNN assigns nearly identical rGEX to predicted spots because the same nearby neighbors dominate across the slide, so the model effectively copies a uniform signal rather than adapting to the new state (Extended Data Fig. 3a). This uniform copying blurs true spatial structure and suppresses gradients that arise from long-range differences. In a Tgfbr2-KO-to-distant-Jak2-KO task, kNN yields flat Plac8 predictions, whereas CONCERT reconstructs high Plac8 in the tumor core and low Plac8 at the surface, recovering the ground-truth spatial pattern (Extended Data Fig. 3e).

#### Niche-dependent propagation patterns.

kNN cannot capture how a change confined to one niche alters responses in adjacent, unperturbed niches because it copies only from immediate neighbors and updates only directly edited spots (Extended Data Fig. 3b). As a result, niche edits show no propagation with neighbor copying: perturbing tumor periphery spots to the normal state leaves predictions for adjacent Jak2 KO spots unchanged under kNN, even though cross-niche signaling should modulate their expression. In this setting, CONCERT registers a downstream effect, detecting a drop in Plac8 from mean NEX 1.415 to 1.358 in the adjacent Jak2 KO niche, consistent with niche-to-niche influence (Extended Data Fig. 3f). Moreover, CONCERT separates intrinsic perturbation effects from extrinsic spatial dependencies, allowing the model to attribute the observed decrease to cross-niche propagation rather than direct editing (Supplementary Fig. 3).

#### Disentangling confounded attributes.

With limited coverage, kNN conflates perturbation with disease state by copying whichever neighbors dominate locally, so confounded attributes cause leakage under neighbor copying (Extended Data Fig. 3c). For normal spots counterfactually set to Jak2 KO normal, kNN overpredicts Plac8 (mean NEX 1.466) by inheriting signal from nearby tumor neighbors (mean NEX 1.473), thereby leaking disease-specific expression into the counterfactual. CONCERT avoids this leakage by modeling condition and context jointly, predicting low Plac8 for the same counterfactuals (mean NEX 1.126) in line with normal tissue profiles (Extended Data Fig. 3g). Consistent with this mechanism, learned kernel cutoffs differ across conditions: CONCERT assigns lower cutoffs to perturbed spots, which expands effective influence fields where appropriate and prevents spurious copying in unperturbed regions (Extended Data Fig. 6).

#### Cross-slide generalization.

kNN relies on within slide neighbors and cannot integrate information across slides that contain different perturbations or sampling densities, so cross slide settings yield no transfer with neighbor copying (Extended Data Fig. 3d). In practice, when we predict a KP tumor patch on GSM5808054 from the unperturbed state to Ifngr2 KO that appears only on GSM5808056, kNN has no relevant neighbors to copy and fails to express the correct shift in rGEX. CONCERT learns perturbation effects as functions of spatial context rather than as slide specific lookups, and therefore transfers across slides: it recovers Fn1 depletion in the tumor core with enrichment at the surface, matching the expected spatial pattern (Extended Data Fig. 3h).

### Finer maps and subspot resolution enhancement

Many sequencing-based spatial transcriptomic assays, including Perturb-map, provide spot-level measurements that limit spatial resolution [45]. CONCERT increases effective resolution by placing virtual spots between observed spots and imputing their gene expression from learned spatial dependencies among nearby observations (Extended Data Fig. 4a). We evaluate resolution enhancement by comparing E-distance against random in-painted patches and report spot-level *R*^2^ to observed tissue, illustrating results with Plac8 and Tnc.

This finer sampling of spots preserves local gradients and boundaries and enables *in silico* perturbation response modeling at higher spatial resolution (Extended Data Fig. 4c). In a region of unperturbed KP tumor (top sub-panel in Extended Data Fig. 4c), we first enhanced resolution (middle sub-panel) and then counterfactually applied Jak2-KO to the densified grid (bottom sub-panel). The enhanced grid was closer to the surrounding KP tumor tissues than to randomly sampled patches (E-distance 7.112 vs. 15.757). After Jak2-KO perturbation, the enhanced grid was also closer to observed Jak2-KO tissues than to random patches (12.322 vs. 19.011). For the marker gene Plac8, imputed expression in the enhanced grid closely matched observed KP tumors (mean NEX 1.235 vs. 1.256) and shifted toward the Jak2-KO profile after perturbation (1.355 vs. 1.544; Extended Data Fig. 4d).

We then compared spot-level correlations by sampling 10 random spots from three sources: (i) the enhanced grid, (ii) corresponding observed tissues, and (iii) the whole slide. For KP tumors, the mean *R*^2^ between the enhanced grid and observed tissues was much higher than that with random spots (0.971 vs. 0.785). A similar pattern was observed for Jak2-KO tumors (0.953 vs. 0.761). These results show that CONCERT increases effective resolution and performs counterfactual prediction at sub-spot scale without additional experiments.

### Filling tissue gaps and predicting perturbation response

We applied CONCERT to impute expression in missing or damaged tissue regions (Extended Data Fig. 4b). In slide GSM5808055, a contiguous gap (red circle) was reconstructed by CONCERT (Extended Data Fig. 4e). After imputation, we applied *in silico* perturbations, converting the imputed spots to Tgfbr2-KO and Ifngr2-KO. The E-distance between predicted and observed perturbed tissue was lower than that to a randomly sampled patch (14.112 vs. 19.847 for Tgfbr2-KO; 11.738 vs. 16.406 for Ifngr2-KO). For the marker gene Tnc, imputed NEX closely matched surrounding spots, and counterfactual rGEX aligned with observed perturbed tissue (1.304 vs. 1.322 for Tgfbr2-KO; 1.252 vs. 1.289 for Ifngr2-KO; Extended Data Fig. 4f). When we randomly sampled 10 spots from (i) predicted perturbed tissues, (ii) corresponding observed tissues, and (iii) the whole slide, the mean *R*^2^ between predicted and observed was consistently higher than that between imputed and random spots (0.961 vs. 0.798 for Tgfbr2-KO; 0.953 vs. 0.811 for Ifngr2-KO). These results show that CONCERT couples imputation with counterfactual simulation to recover perturbation effects in regions not directly observed experimentally.

We then evaluated missing-data imputation by masking observed spots and asking CONCERT to recover their expression. Two sampling strategies were used: (1) fully random (stochastic) masking and (2) masking restricted to border spots (Extended Data Fig. 4g). Border-focused tests intentionally stress models because boundaries contain sharp transitions and greater heterogeneity. We sampled 10, 20, 40, and 80 spots per experiment and compared CONCERT to bioLORD-GCN, a generative baseline that supports both counterfactual prediction and imputation (Methods). With stochastic masking, CONCERT exceeded bioLORD-GCN by 0.098, 0.120, 0.094, and 0.078 in test-set *R*^2^ for 10, 20, 40, and 80 spots, respectively (Extended Data Fig. 4h). Under boundary-focused masking, where spots lie at interfaces between distinct tissue regions, the margins increased to 0.215, 0.255, 0.282, and 0.309. This highlights CONCERT’s advantage in predicting perturbation responses in regions where gene expression changes sharply across tissue boundaries.

### Perturbation responsess depend on the microenvironment

In Perturb-map, tumor-CRISPR vector infection occurs *in vivo*, so researchers cannot control which spots receive a perturbation or where a given perturbation lands [12] (Supplementary Fig. 1). CONCERT overcomes these constraints by performing *in silico* perturbation with spatially informed prediction. We present two analyses that highlight how perturbation outcomes depend on the microenvironment.

#### Transplantation across niches.

**Figure 5:**
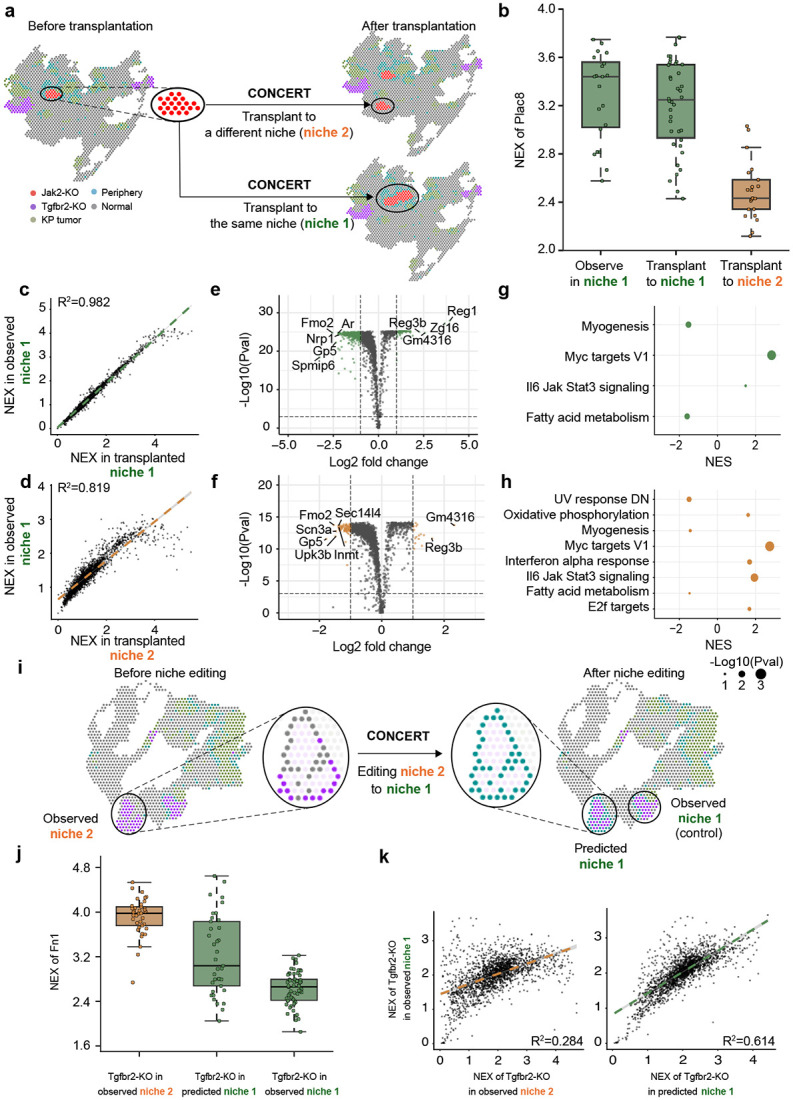
CONCERT predicts how perturbation outcomes change across microenvironments. **a** Transplantation task schematic and application to perturb-map data. Jak2-KO tumor spots from the core (niche 1) were “transplanted” into two distinct contexts: another tumor core (niche 1) and a tumor surface region surrounded by normal tissue (niche 2). **b.** Expression of Plac8 before and after transplantation. Plac8 remained high in tumor-core context (mean NEX 1.443) but decreased in the surface niche (mean NEX 1.231). **c, d.** Gene expression of spots observed in niche 1 (*R*^2^ = 0.982) shows a stronger correlation with those predicted in niche 1 than the spots predicted in niche 2 (*R*^2^ = 0.819). **e, f.** Differential expression (DE) analysis between transplanted regions and normal tissue. **g, h.** Pathway analysis of DE results using Hallmark gene sets [46] identified oxidative phosphorylation and interferon-alpha response (normalized enrichment scores 1.851 and 1.852) as uniquely upregulated at the tumor surface, consistent with prior studies. Pearson correlations show high agreement between original and transplanted spots within core (top), and divergence between original core and transplanted surface (bottom). **i.** Niche-editing task schematic and application to perturb-map data. Observed Tgfbr2-KO spots in a normal-like surface niche (niche 2) were edited by altering their surrounding context to resemble tumor core (niche 1). j. Fn1 expression shifted toward the true tumor-core profile: mean NEX decreased from 3.98 in niche 2 to 3.01 after editing, approaching the observed value in tumor core (2.68). Boxplots summarize Fn1 changes across conditions. **k.** Pearson correlations confirm that CONCERT predictions in the edited niche align more closely with true niche 1 than with the original niche 2. Boxplots show the median (center line), first and third quartiles (box edges), and whiskers extending 1.5× the interquartile range. NEX: normalized gene expression; NES: normalized enrichment score.

On slide GSM5808054, we asked whether the same perturbation produces different responses when placed into distinct microenvironments (Fig. 5a). Starting from Jak2-KO spots located in a tumor-core context, we “transplanted” the patch into another core and into a surface-like region surrounded by normal tissue. Although the perturbation state was identical, CONCERT produced distinct rGEX profiles that reflected each niche (Fig. 5b). Plac8 remained high when surrounded by tumor periphery spots (mean NEX 3.20) but decreased in a normal environment (mean NEX 2.491). The gene expression of spots observed in tumor core (niche 1) shows a stronger correlation with the spots predicted in tumor core (*R*^2^ = 0.982) than with those predicted in the surface-like region (niche 2, *R*^2^ = 0.819, Fig. 5c-d). Differential expression and hallmark enrichment [46] identified pathways selectively upregulated at the tumor surface, including oxidative phosphorylation and interferon-alpha response (normalized enrichment scores 1.627 and 1.693), consistent with prior studies [47, 48] (Fig. 5e-h; Supplementary Fig. 31).

#### Perturbing the surrounding niche.

Instead of moving the patch, we altered its environment. We selected observed Tgfbr2-KO spots in a normal-like niche and *in silico* perturbed the surrounding spots to resemble tumor periphery (Fig. 5i). CONCERT shifted the NEX profile of the central Tgfbr2-KO patch toward that of the actual tumor-periphery niche: for Fn1, mean NEX decreased from 3.917 to 3.228, approaching the observed value in tumor periphery (2.625) (Fig. 5j). Agreement with the target niche improved, with *R*^2^ increasing from 0.284 in the starting niche to 0.614 in the perturbed niche relative to the tumor-periphery reference (Fig. 5k). These results show that CONCERT captures how the same genetic perturbation can yield different outcomes across niches and how environmental changes propagate to nearby spots.

### Tracking inflammation and recovery over time in colitis

**Figure 6:**
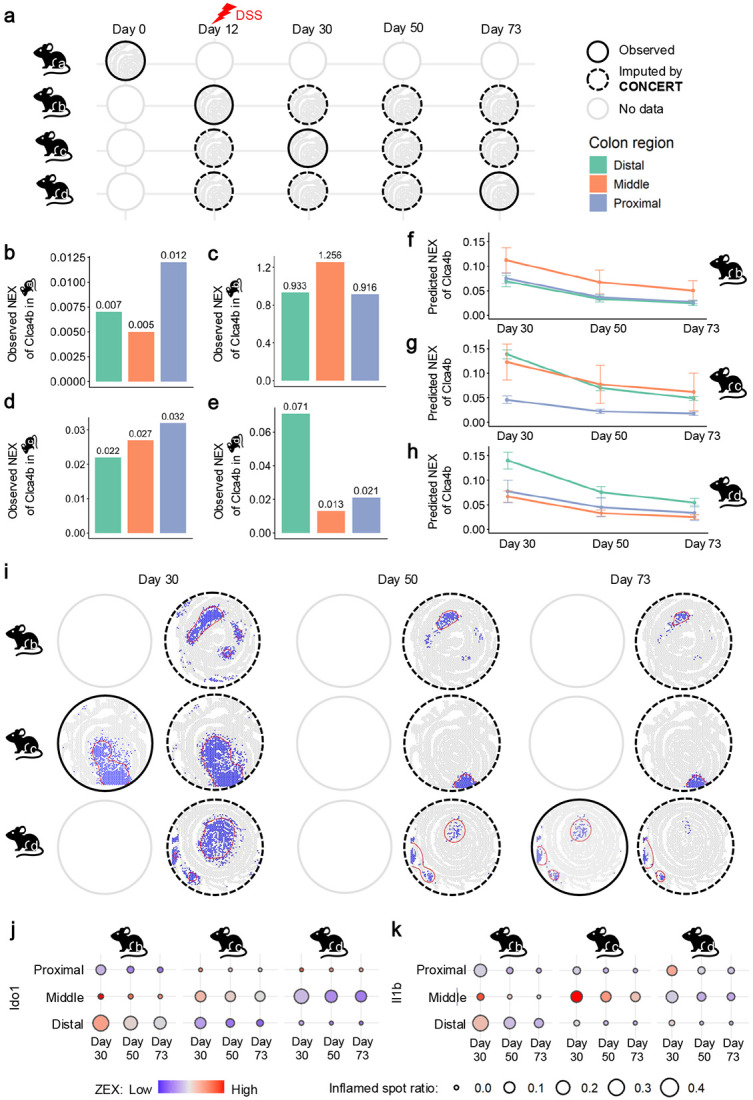
CONCERT reconstructs the dynamics of recovery from inflammation in colitis by imputing missing time points. **a.** Experimental setup. In the DSS colitis model, each mouse was sacrificed at a single time point, so direct longitudinal tracking within the same animal is not possible. CONCERT imputes missing time points for each mouse, generating complete temporal trajectories. Numbers indicate sample sizes at observed time points; day 50 is fully imputed by CONCERT. Solid lines show observed data; dotted lines show imputed values. **b-e.** Spatial distribution of Clca4b expression reveals apparent shifts in inflammation across mice at day 30 and day 73, reflecting inter-mouse variability. **f-h.** CONCERT imputations resolve this variability, showing a consistent decline in Clca4b expression across colon regions from day 30 to day 73, with day 50 predictions fitting the recovery trajectory. **i.** Predicted inflamed regions for each mouse, identified using the marker gene Clca4b with a quantile 0.85 cutoff. **j-k.** Other inflammation markers, Ido1 and Il1b, follow the same region-wise recovery trend, confirming reproducible resilience dynamics.

We applied CONCERT to spatial transcriptomics of mouse gut tissues perturbed by dextran sodium sulfate (DSS), which induces colon inflammation [41]. To study how inflammation changes over time, DSS was administered on day 12, and tissues were collected at three time points: day 0 (pretreatment), day 30, and day 73 (post-treatment). Because each mouse is sacrificed at a single time point, the same animal cannot be tracked across time (Fig. 6a), complicating temporal comparisons. In this dataset, we observed a spatial mismatch in inflammation between day 30 and day 73. The inflammation marker Clca4b showed proximal-dominant inflammation on day 30 (mean NEX for distal and proximal regions: 0.012 and 0.018) but shifted to the distal colon on day 73 (0.036 and 0.012; Fig. 6b-e). Such a relocation is unlikely to reflect true recovery dynamics and more likely arises from inter-mouse variability [49, 50]. These inconsistencies hinder meaningful region-wise comparisons.

CONCERT addresses this limitation by predicting gene expression across time. By combining spatial and temporal information, CONCERT estimates rGEX at unseen time points for each mouse. Together with observed days 0-35, the imputed day 50 provides a complete trajectory. Imputed day-50 slices facilitate consistent region-wise recovery despite cross-mouse variability. For that, we trained CONCERT on all observed time points and predicted rGEX at three recovery stages: day 30, day 50 (unseen), and day 73. To track inflammation, we used marker genes Clca4b, Ido1, and Il1b from the original study [41]. Time was encoded as a continuous attribute, enabling interpolation within the observed range. Predictions showed that inflammation markers consistently declined from day 30 to day 73 within each colon region (Fig. 6f-h for Clca4b; Supplementary Fig. 33, Supplementary Fig. 35, Supplementary Fig. 37, and Supplementary Fig. 39 for other markers), consistent with expected healing dynamics [49, 50]. Using a 0.85 quantile threshold on marker expression to define inflamed regions, we observed region-wise recovery that was consistent across mice (Fig. 6i for Clca4b; Supplementary Fig. 34, Supplementary Fig. 36, Supplementary Fig. 38, and Supplementary Fig. 40 for other markers). Ido1 and Il1b followed the same trend (Fig. 6j-k).

### Modeling stroke effects in the brain

We applied CONCERT to a spatial transcriptomics dataset of mouse brain perturbed by photothrombosis (PT), which induces focal ischemic stroke [42]. The dataset includes sections from two mice (one PT, one sham control). Spatial profiles were collected from four consecutive sections at distances of 0.4 mm, 0.8 mm, 2.0 mm, and 3.2 mm from the stroke core. By combining each slide’s 2D coordinates with inter-slide spacing along the *z*-axis, we constructed a 3D coordinate system that allows CONCERT to model stroke-induced dispersion with a 3D kernel (Extended Data Fig. 5a). All predictions were made on sham samples. CONCERT supports user specified lesion footprints and models 3D dispersion across serial sections, enabling lesion aware propagation beyond a single slice. Core regions are indicated by Gm42418, while peri-lesion responses are marked by Lnc2 and Spp1.

#### 2D cortical simulations.

Because PT provides limited control over lesion size and affects a single cortical region, we designed two 2D experiments (Extended Data Fig. 5bc). (i) To simulate multi-regional ischemia, we selected varying numbers of random cortical spots (*n* = 10, 20, 40, 80, 100, 200, Extended Data Fig. 5b). (ii) To study lesion-size effects, we sampled a cortical centroid and varied the size of the affected region (*n* = 5, 10, 20, 40, 80, 160, Extended Data Fig. 5c). The results show strong spatial dependencies: scattered ischemic spots yield weak transcriptional signals, whereas denser clusters amplify responses through overlapping influence; larger patches extend effects across broader regions. CONCERT captured gene-specific patterns, correctly predicting Gm42418 as core-maximal (Extended Data Fig. 5b) and Lnc2 as peri-lesion-enriched and suppressed in the core (Extended Data Fig. 5c), consistent with additional markers (Supplementary Fig. 44, Supplementary Fig. 45, Supplementary Fig. 46). Marker genes were chosen following the original study, including Gm42418, Supp1, and Lnc2 (Extended Data Fig. 5b; Supplementary Fig. 41, Supplementary Fig. 42, Supplementary Fig. 43).

#### 3D propagation across sections.

We next trained a 3D version of CONCERT using spatial coordinates from all four sections (Extended Data Fig. 5d). PT and sham slides were aligned separately using anatomical region labels from the original study (Supplementary Fig. 47). Cross-slide neighborhood analysis confirmed connectivity across sections: as neighborhood size increased, spots from different slides became interlinked, validating the 3D graph [51, 52] (Supplementary Fig. 48). Predictions showed clear *z*-axis propagation of stroke effects. For example, Gm42418 expression was highest in the first section (Sham 1), diminished in the second (Sham 2), and was nearly absent in the third and fourth (Sham 3-4; Extended Data Fig. 5e). This progression closely mirrored the experimentally observed spread in PT mice (Extended Data Fig. 5d).

## Discussion

**Extended Data Fig. 1:**
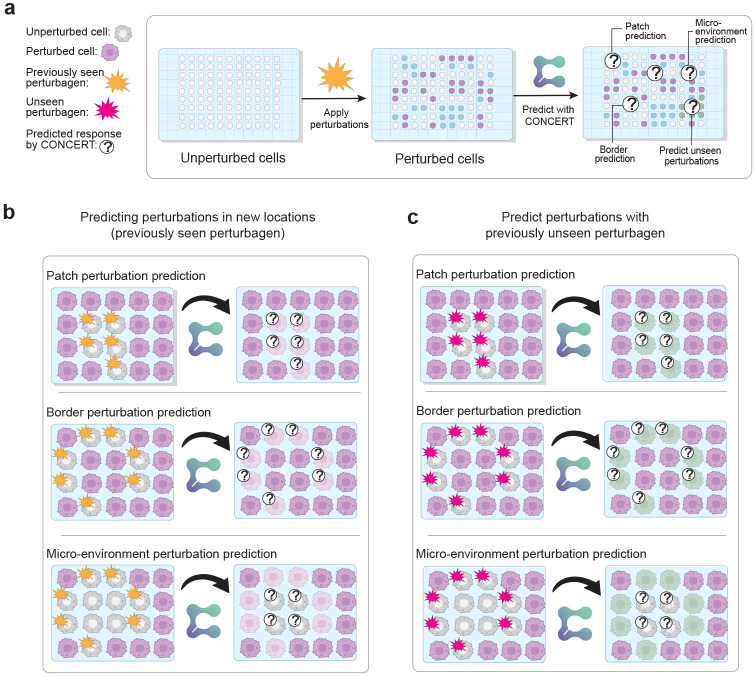
Novel tasks designed for spatial perturbation transcriptomic data. (a) An example workflow to experimentally perturb spots on a spatial transcriptomic slide and predict perturbations on the specified spots. (b) and (c) demonstrate the novel tasks designed for spatial perturbation transcriptomic data. (b) predict seen perturbations to the new locations, including patch, border, and micro-environment perturbations; (c) predict unseen perturbations from different slides to new locations. For micro-environments perturbation, we perturb the niche, the surrounding cells of the target cells, by a model, and then use the model to predict the post-perturbation gene expression of the target cells.

We present CONCERT, a spatially aware model designed to overcome limitations of traditional perturbation models by integrating spatial information into counterfactual prediction. We introduce novel tasks for spatial perturbation transcriptomic data, namely patch, border, and niche, that simulate key biological phenomena: interactions within cellular clusters, boundary dynamics between cell types, and the influence of local tissue microenvironments (Extended Data Fig. 1). Across these tasks, CONCERT consistently outperforms existing perturbation prediction models. Two case studies extended beyond benchmarking. In mouse colitis, CONCERT imputed unmeasured time points and enabled longitudinal comparisons across animals, recovering region-specific inflammation-recovery trends that are obscured by inter-mouse variability [41]. In photothrombotic stroke, CONCERT simulated location- and lesion-size specific responses in 2D and extended predictions into 3D, capturing lesion-core and peri-lesion patterns observed experimentally [42]. CONCERT employs a set of inducing points to reduce the cubic computational complexity of GP to near-linear. This design enables efficient kernel learning across thousands of spots. The GP formulation with learnable kernel scales and cutoffs in CONCERT enables the model to capture anisotropic, long-range, and non-uniform decay patterns that emerge as intrinsic properties of the tissue.

Several limitations remain. Spatial perturbation datasets are still scarce, and our evaluation used a small number of Visium slides from perturb-map lung tissues with CRISPR knockouts and two mouse case studies. Generalization to other tissues and perturbation types (for example, CRISPRi/a, pharmacologic agents, cytokines) and to other spatial platforms with different resolutions or gene panels is untested. Batch effects and inter-sample variability can persist despite normalization and may influence kernel learning. CONCERT predicts post-perturbation transcriptomes at the spot level but does not explicitly model phenotypes, so conclusions should be interpreted as transcriptomic rather than functional predictions. The spatial module learns perturbation-specific Gaussian process kernels that are stationary within a slide and do not yet encode histology-derived boundaries or compartment priors, which may smooth across sharp interfaces or underweight tissue barriers. Uncertainty calibration was assessed in-distribution and may not hold under strong domain shifts. CONCERT also does not incorporate priors such as drug chemistry or gene regulatory networks, which could support zero- or few-shot generalization. Finally, missing spots, damaged regions, and technical dropouts can bias spatial kernels. While CONCERT imputes missing data, imputations may propagate artifacts when large areas are absent.

These limitations point to clear next steps. Prior work in single-cell perturbation transcriptomics shows that incorporating biological priors improves generalization [22, 24, 53]. Although these approaches are not designed for spatial data, they demonstrate that embedding drug structures and gene-regulatory networks strengthens mechanistic interpretability. Extending these strategies to spatial perturbation transcriptomics would let CONCERT predict how perturbation effects propagate across tissue niches. Another direction is to integrate histology and anatomical priors, extending spatial kernels in CONCERT to encode tissue-aware anisotropy in 3D while modeling tissue borders. Kernel fields can encode resection margins, lesion ablation footprints, or drug penetration maps, positioning CONCERT as a hypothesis generator for tissue targeted interventions.

CONCERT performs counterfactual prediction within a spatial framework and introduces benchmarks that test how microenvironments shape responses. It provides a data-driven way to model perturbation effects in tissue and opens paths toward therapeutic modeling and tissue-targeted interventions.

## Methods

The Methods section discusses (1) Datasets used in benchmarking experiments, model validation, and case studies; (2) Details about the architecture and optimization of CONCERT; and (3) Details about model validation and applications.

## Datasets

### Perturb-Map datasets.

We tested CONCERT on the Perturb-map datasets for mouse lung adenocarcinoma (GSE193460) with four slides of samples. The spots’ annotations are provided in the original paper using imaging cytometry [12]. Briefly, the GSM5808054 and GSM5808055 samples contain 1,903 and 1,872 spots, respectively, and feature two types of genetic perturbation: Jak2 gene knockout (denoted as Jak2-KO) and Tgfbr2-KO, as well as Ifngr2-KO and Tgfbr2-KO. The GSM5808056 and GSM5808057 samples contain 1,233 and 1,355 spots, respectively, and each has one perturbation, namely Ifngr2-KO and Tgfbr2-KO. All sample slides also have annotated unperturbed tumor spots, tumor periphery spots, and normal spots on the tissue. We used Seurat [54] to preprocess the data downloaded from GEO and transformed them into H5 or H5AD files as input for the models. The count data matrix is normalized by SCANPY [55] as input. In total, this dataset contains 6,363 spots with 21 Jak2-KO spots, 224 Tgfbr2-KO spots, 105 Ifngr2-KO spots, and 6,013 unperturbed spots.

### Mouse colon datasets.

The processed mouse colon spatial transcriptomic data (ST) is provided by the original paper [41]. To investigate the resilience of the spatial landscape of the colon, the authors induced inflammation in mice using dextran sodium sulfate (DSS) and mapped the landscape throughout the post-treatment recovery courses. This dataset contains four slides of tissues sampled in different times: day 0 (before perturbation), day 12 (conducting perturbation), day 30 (after perturbation), and day 73 (after perturbation), with 2,918, 3,494, 3,497, and 3,245 spots, respectively. We combined all the samples with 13,154 spots. The count data matrix is normalized by SCANPY as the input. The colon regions, namely the proximal, middle, and distal colon, are provided in the original paper. In total, this data contains 13,154 spots with 5,540 in the proximal region, 4,908 in the middle region, and 2,706 in the distal region.

### Mouse stroke datasets.

The mouse ischemia spatial transcriptomics dataset used in this study is derived from Han et al. [42]. This dataset includes brain tissue slides from two donor mice, one subjected to photothrombosis (PT) to induce ischemic stroke in the cortex, and one sham control without PT. For each mouse, four brain slides were collected at distances of 0.4 mm, 0.8 mm, 2.0 mm, and 3.2 mm from the stroke core. The PT mouse slides contain 2,397, 2,650, 2,781, and 2,755 spots, while the sham mouse slides contain 1,752, 2,031, 2,618, and 2,793 spots, respectively. To build a 3D coordinate system, we aligned the four slides by brain regions (labels are provided in the original paper) and used the distances of the slides to the core of stroke as the third dimension. The count data matrix is normalized by SCANPY as the input. In total, this dataset contains 10,583 PT and 9,194 sham spots; in PT spots, there are 511 spots in the central ischemic region and 1303 spots in the periphery ischemic region. Other spots are almost unaffected by PT.

## CONCERT model

### Model overview.

We aim to predict cellular responses to perturbations while accounting for their surrounding microenvironment. To this end, we present CONCERT, a deep learning framework designed for the prediction of spatial perturbations. CONCERT integrates three key modules: (i) a perturbation module that disentangles spatial perturbation transcriptomics (SPT) data in latent space and enables *in silico* perturbations; (ii) a spatial module that models spatially resolved, cell-specific perturbation effects; and (iii) a generative module that reconstructs gene expression profiles corresponding to the predicted perturbation responses. CONCERT takes as input cells’ gene expression profiles, spatial coordinates, and perturbation states, and outputs predicted transcriptomic responses that incorporate each cell’s tissue context. Furthermore, CONCERT can impute missing cells on tissue slides and estimate their responses to perturbations. In this section, we first present the problem formulation of CONCERT. We then introduce its perturbation, spatial, and generation modules along with the corresponding objective functions. Finally, we describe how CONCERT is applied to address the problems and provide details on its implementation across the datasets.

### Preliminaries.

Uppercase X denotes a matrix and lowercase x denotes a vector; uppercase 𝒳 denotes a set of observations or variables. We denote p(X) as a probability distribution over a set of random variables X and p(X=x) as the probability of X that is equal to the value of x under the distribution p(X). This section uses terminology and concepts from the framework of variational inference [56].

### Problem formulation: disentangle the perturbation effects.

Given cells’ gene expression profile $X\in R^{N\times G}$ of *N* cells and *G* genes, their coordinates in tissue space $S\in R^{N\times 2}$, and categorical label of perturbation state $l_{p}\in \{1,\ldots ,k\}$ with *k* perturbations $p_{0},p_{1},\ldots ,p_{k}\in 𝒫$ ($p_{0}$ is the state without perturbation), the goal is to disentangle $X\in R^{N\times G}$ in to $E_{s}$, the embedding of cell state independent to perturbation, and $E_{p}$, the embedding of perturbation effects:

(1) $$f_{\phi }(X,S,l_{p})=(E_{s},E_{p})\in R^{N\times L},$$

$f_{\phi }\;:𝒜\to ℬ$, typically denotes mapping a set 𝒜 to a set ℬ and $X,S,l_{p}$ are not sets, but specific elements of the set.

where $f_{\phi }$ is a function with learnable parameter ϕ, and *L* is the dimension of the embeddings. Then, we use the disentangled embeddings to reconstruct the cells’ gene expression profile X by a function $f_{\theta }$ with learnable parameters θ:

(2) $$f_{\theta }(E_{s},E_{p})=\hat{X}\in R^{N\times G},$$

(3) $$\phi^{*},\theta^{*}=\underset{\phi ,\theta }{\text{argmax}}\;\log\;p(\hat{X}\;|\;X,\phi ,\theta )$$

where *p* is an appropriately chosen density function. This function ensure that the disentangled embeddings $E_{s}$ and $E_{p}$ preserve information in X necessary for reconstruction.

### Problem formulation: Spatial perturbation prediction.

Given 𝒞⊆{1,…,N}, ∣𝒞∣=M a set of *M* cells without perturbation ($I_{p}$ is $p_{0}$), we get their disentangled embeddings $E_{s}^{C}$ and $E_{p}^{C}$. With 𝒫, a set of perturbations observed in the cells in X, the goal is to counterfactually predict response gene expression of the cells in 𝒞 to a perturbation $p_{1}\in 𝒫$:

(4) $$f_{\omega }(E_{p}^{C},p_{1})=E_{p}^{C(p_{1})}\in R^{M\times L},$$

(5) $$f_{\theta }(E_{s}^{C},E_{p}^{C(p_{1})})=X_{p}^{\prime C}\in R^{M\times G},$$

where $f_{\omega }$ is a function with learnable parameters ω that *in silico* perturb the perturbation embedding of cells in 𝒞 from $E_{p}^{C}$ to $E_{p}^{C(p_{1})};X_{p}^{\prime C}$ is the predicted post-perturbation expression profile of the cells in 𝒞,

### Problem formulation: cell imputation with perturbation prediction.

Given the embedding, $E=[E_{s}∥E_{p}]$ (where ∥ stands for row-wise concatenation), for a set of observed cells, together with spatial coordinates $S^{u}\in R^{M_{u}\times 2}$ for additional $M_{u}$ unseen cells in a set, 𝒰, whose expression profiles are missing, the objective is to first use a function $f_{\pi }$ to impute the embedding of missing cells:

(6) $$f_{\pi }(E,S^{U})=(E_{s}^{U},E_{p}^{U})\in R^{M_{u}\times L},$$

where $E_{s}^{U}$ and $E_{p}^{U}$ are the imputed embeddings of the unseen cells 𝒰. Then we perform *in silico* perturbation on $E_{p}^{U}$:

(7) $$f_{\omega }(E_{p}^{U},p_{1})=E_{p}^{U(p_{1})}\in R^{M_{u}\times L},$$

with perturbation $p_{1}\in 𝒫$, and finally use the perturbed embedding $E_{p}^{U(p_{1})}$ to generate the perturbed gene expression profile $X_{p}^{\prime U}$ of the unseen cells in 𝒰 :

(8) $$f_{\theta }(E_{s}^{U},E_{p}^{U(p_{1})})=X_{p}^{\prime U}\in R^{M_{u}\times G},$$

### Perturbation module: embedding learning and disentanglement.

In CONCERT, to predict the perturbation, we disentangle the gene expression profiles of cells in the latent space following the framework of LORD, a machine learning method based on Latent Optimization for Representation Disentanglement [57]. The effectiveness of this approach for disentangling single cell attributes and perturbations has been demonstrated in bioLORD [23], a deep-generative model for single cell perturbation transcriptomic data. Building on this framework, CONCERT decomposes complex gene expression profiles (GEX) into distinct embeddings corresponding to cell attributes of interest, including categorical variables such as the state of perturbation and the state of the disease, as well as continuous variables such as the time after the perturbation and the size of the tumor. A regularized spot-specific embedding is also learned for each data point to capture individual cellular heterogeneity, serving as the basal latent space. Furthermore, if the input includes multiple spatial slides, a slide-specific embedding is incorporated to enable data integration across samples/slides. In CONCERT, perturbations are applied to the cells before modeling cell-cell dependencies. This design allows the perturbation signal to propagate to neighboring cells, facilitating the modeling of the context-aware perturbation effects. CONCERT is applicable to both cell- and spot-level data; as all datasets in this study are spot-level, we refer to each data entry as a spot in the following parts of this section.

We illustrate our algorithm using the Perturb-map dataset as an example, which includes two attributes: perturbation state and disease state. In practice, various other attributes can be incorporated depending on the specific goals of the study. To disentangle the Perturb-map data, we randomly initialize three codebooks: the spot codebook: $E_{s}\in R^{N\times m_{1}}$, where *N* is the number of spots; the disease state codebook $E_{d}\in R^{N_{d}\times m_{2}}$ for diseases $d_{0},d_{1},\ldots ,d_{N_{d}}$, where $d_{0}$ indicates the spot state without disease and $N_{d}$ is the number of diseases observed in data; and the perturbation state codebook $E_{p}\in R^{N_{p}\times m_{3}}$ for perturbations $p_{0},p_{1},\ldots ,p_{N_{p}}$, where $p_{0}$ indicates the spot state without perturbation and $N_{p}$ is the number of perturbation states. Here, *m*, *m*_2_ , and *m*_3_ are the dimensionalities of the embeddings set to 256 by default. During training, $E_{s}$ learns the intrinsic heterogeneity of gene expression independent of other spot attributes, serving as a basal latent representation, as defined in CPA [20]. The disease and perturbation states are learned in $E_{d}$ and $E_{p}$, respectively. For a continuous variable c, we apply MLP layers, $f_{p}$, to project the 1-dimensional vector to the latent dimension *m*_4_, as $E_{c}=f_{p}(c)$, where $E_{c}\in R^{N\times m_{4}}$.

For a spot *k*, it will have the embedding of disease state, $e_{d}^{k}\in E_{d}$, the embedding of perturbation states, $e_{p}^{k}\in E_{p}$, and the embedding of basal latent space, $e_{s}^{k}\in E_{s}$. To prevent spot embedding $e_{s}^{k}$ from learning the information that belongs to the embeddings of disease state $e_{d}^{k}$ and perturbation state $e_{p}^{k}$, a regularization is imposed to $e_{s}^{k}$ by adding a Gaussian noise:

(9) $$\epsilon ∼𝒩(0,\sigma^{2}),$$

(10) $$e_{s’}^{k}=e_{s}^{k}+\epsilon ,$$

In our default setting, σ is set as 0.1 for all datasets.

All learnable embeddings for spot *k* are concatenated as $e_{k}$, serving as a latent representation of it (Figure 2A). Since $e_{d}^{k}$ and $e_{p}^{k}$ are known attribute embeddings and $e_{s}^{k}$ are unknown attribution embeddings, after training, the concatenation of them is considered to contain the full information to reconstruct the complex spatial perturbation transcriptomic data. Formally, the latent representation of the spot *k* is defined as:

(11) $$e^{k}=⊕(e_{s’}^{k},e_{d}^{k},e_{p}^{k}),$$

for single slide in put, or

(12) $$e^{k}=⊕(e_{s’}^{k},e_{c}^{k},e_{p}^{k},e_{l}^{k})$$

for multi-slide input with slide embedding $e_{l}^{k}$. Here, ⊕ indicates matrices row-wise concatenation. In the following sections, we mention the embedding of a spot, $e^{k}$, as e, for convenience.

### Spatial module: capturing spatial dependencies.

Variational autoencoders (VAEs) [58] are powerful models for learning complex data distributions in an unsupervised manner, but their effectiveness is limited by the assumption that latent representations are independent and identically distributed. The GP-VAE [59-61] overcomes this limitation by integrating Gaussian process (GP) priors, enabling the modeling of correlations in latent spaces. Formally, for input data x∈X, its latent distribution z(x) follows:

(13) $$z(x)∼𝒢𝒫(m(x),k(x,x^{\prime })),$$

where $m(x)=f_{e}^{\mu }(x)$ and $k(x,x^{\prime })\;=\;f_{e}^{\sigma }(x)f_{e}^{\sigma }{\left(x^{\prime }\right)}^{T}$. $f_{e}^{\mu }$ and $f_{e}^{\sigma }$ are the encoders with neural network layers for mean and variance, and x and $x^{\prime }$ are two spots in the data matrix X.

Based on this, spaVAE [26] leverages GP-VAE to learn spatially dependent latent representations of spots in spatially resolved transcriptomics data, where kernel values between two spots are calculated based on their spatial locations. It shows superior performance in capturing spatial patterns and facilitating downstream analyses.

Inspired by these advancements, CONCERT uses GP-VAE to capture spatial cell-cell dependencies based on their locations on tissue slides, learning soft dependencies between spots without defining arbitrary K neighbors, as in kNN-based approaches. This approach allows CONCERT to effectively infer the scope and magnitude of perturbation effects on the tissue slide.

### Part 1 - prior formulation.

To capture the spatial and non-spatial effects of perturbation, CONCERT learns a GP latent space considering the spatial location of spots and a Gaussian latent space ignoring spots’ location. Based on the GP framework described above, the GP latent space of CONCERT, $z^{\text{GP}}$, is learned using a Gaussian process prior:

(14) $$z^{\text{GP}}∼𝒢𝒫(0,k^{p}(\cdot ,\cdot )),$$

where $k^{p}(\cdot ,\cdot )$ is the kernel function for perturbation *p*, defining the covariance structure based on spatial locations of spots. In the default setting, we use the anisotropic Cauchy kernel to simulate the decay of spot-spot dependencies over physical space. Given two spots, *a* and *b*, with spatial coordinates, $s^{a}\in R^{D}$ and $s^{b}\in R^{D}$, in dimensionality *D*, perturbed by perturbation *p*, the anisotropic Cauchy kernel $k^{p}(\cdot ,\cdot )$ with the learnable kernel scale per spatial dimensionality, $l^{p}\in \{l_{1}^{p},l_{2}^{p},\ldots ,l_{D}^{p}\}$, is defined as:

(15) $$k^{p}(s^{a},s^{b})=\frac{1}{1+\sum_{i=1}^{D}\frac{{\left(s_{i}^{a}-s_{i}^{b}\right)}^{2}}{l_{i}^{p}}}.$$

To learn a more flexible spatial effect of perturbation, each spot has a learnable cutoff that control the scope of its kernel. Here, to enforce a learnable cutoff for spot *a* and *b* at threshold $c_{a}$, $c_{b}\in (0,1)$, the masked kernel $k_{m}^{p}(a,b)$ is defined as:

(16) $$c=\frac{1}{2}\;(c_{a}+c_{b}),$$

(17) $$\text{mask}=\sigma (-\tau \cdot (k^{p}(s^{a},s^{b})-c)\;,$$

(18) $$k_{m}^{p}(s^{a},s^{b})=k^{p}(s^{a},s^{b})\cdot \text{mask},$$

τ is the scale parameter that controls the sensitivity of the kernel to the distance between two spots: a larger scale makes the kernel less sensitive to changes in distance, while a smaller scale increases sensitivity, emphasizing closer relationships between spots. The default value of τ is 1. No mask matrix is used for the kernel when the cutoff, *c*, is set to 0. In single-kernel mode, CONCERT learns one kernel with a single learnable scale.

On the other hand, the non-spatial component $z^{G}$ follows a standard normal distribution:

(19) $$z^{G}∼𝒩(0,I),$$

which is independent of the spatial dependencies among the spots.

### Part 2 - encoder for variational inference.

The concatenated embedding of a spot, $e\in R^{m_{1}+m_{2}+m_{3}}$, is encoded by MLP encoders, $f_{e}^{\mu }(.)$ and $f_{e}^{\sigma }(.)$, which map e to latent variables z, parameterizing an approximate variational posterior $q(z\;|\;x,s,𝒞)=q(z_{\text{GP}}\;|\;x,s,𝒞)\;q(z_{G}\;|\;x,𝒞)$. Here, s indicates the spatial location of a spot; 𝒞 indicates a set of covariates, such as diseases and perturbations; z is parameterized by mean $\mu_{q}$ and variance $\sigma_{q}^{2}$:

(20) $$m_{q},\sigma_{q}^{2}=f_{e}^{\mu }(e),f_{e}^{\sigma }(e),$$

Here, $\mu_{q}$ and $\sigma_{q}^{2}\in R^{\left(dim_{gp}+dim_{guassian}\right)}$ are split into GP and Gaussian parts:

(21) $$\mu_{q}=\left[\begin{array}{c} \mu_{\text{GP}}\\ \mu_{\text{Gaussian}} \end{array}\right],\;\sigma_{q}^{2}=\left[\begin{array}{c} \sigma_{\text{GP}}^{2}\\ \sigma_{\text{Gaussian}}^{2} \end{array}\right],$$

where

(22) $$\mu_{{\text{GP}}_{q}}=\mu_{q}[:dim_{gp}],$$

(23) $$\mu_{\text{Gaussian}}=\mu_{q}[dim_{gp}\;:],$$

(24) $$\sigma_{{\text{GP}}_{q}}^{2}=\sigma_{q}^{2}[:dim_{gp}],$$

(25) $$\sigma_{\text{Gaussian}}^{2}=\sigma_{q}^{2}[dim_{gp}\;:].$$

Here, $\mu_{{\text{GP}}_{q}}$ and $\sigma_{{\text{GP}}_{q}}^{2}$ are the prior latent variables to capture spatial dependencies and follow a Gaussian process (GP) prior; $\mu_{\text{Gaussian}}$ and $\sigma_{\text{Gaussian}}^{2}$ are the prior latent variables to captures non-spatial variations and follows a standard Gaussian prior. The $dim_{gp}$ is the dimension of GP latent space, $z^{\text{GP}}$, and is set as 2 in the default setting. Similarly, $dim_{guassian}$ is the dimension of the Gaussian latent space, $z^{G}$, and is set to 8 in the default setting. So, the default total dimension of the latent space, z, is 10. The prior latent variable matrices for all spots are denoted using capital letters: $M_{GP_{q}}$, $\Sigma_{GP_{q}}^{2}$, $M_{Gaussian}$, $\Sigma_{Gaussian}$ and Z.

### Part 3 - inducing-point approximation.

Following spaVAE [26], we use inducing points to approximate posterior variables of GP. To obtain inducing points 𝒫, we generate a uniform grid in the spatial domain:

(26) 𝒫=(([xy]∈[0,1]×[0,1]∣x,y=0,1r,2r,…,1+ϵ0)⋅j,

where j is the number of inducing point steps with default value 6, $\epsilon_{0}$ is a small buffer to ensure coverage at the boundary with default value 1e−5, and *r* is the scaling factor for the spatial coordinate range with default value 20.

For clarity, we first define the notation for the kernel matrices used in this approximation. In the following sections, we use capital *T* to indicate the training data variables and lower case *t* to indicate those for the test data. Then $S_{t}$ and $S_{T}$ stand for the coordinates of training and testing spots. We define the following kernel matrices: $K_{PP}=k(𝒫,𝒫)$ as the kernel matrix computed over the inducing points 𝒫; $K_{S_{t}S_{t}}=k(S_{t},S_{t})$ as the kernel matrix of the testing points; $K_{S_{t}P}=k(S_{t},𝒫)$ and $K_{PS_{t}}=k(𝒫,S_{t})$ as the cross-covariance matrices between the testing points and the inducing points; and $K_{S_{T}P}=k(S_{T},𝒫)$ and $K_{PS_{T}}=k(𝒫,S_{T})$ as the kernel matrices between training data and inducing points. The posterior mean for the testing points is given by:

(27) $$M_{{\text{GP}}_{p}}=\frac{N_{T}}{N_{b}}K_{S_{t}P}\Sigma_{l}^{-1}K_{PS_{T}}\frac{M_{{\text{GP}}_{q}}}{\Sigma_{{\text{GP}}_{q}}^{2}},$$

where $\Sigma_{l}$ is defined as:

(28) $$\Sigma_{l}=K_{pp}+\frac{N_{T}}{S_{b}}K_{PS_{T}}\frac{K_{S_{T}P}}{\Sigma_{{\text{GP}}_{q}}^{2}},$$

and its inverse is given by:

(29) $$\Sigma_{l}^{-1}={\left(K_{PP}+\frac{N_{T}}{S_{b}}K_{PS_{T}}\frac{K_{S_{T}P}}{\Sigma_{{\text{GP}}_{q}}^{2}}\right)}^{-1},$$

On the other hand, the covariance matrix of the testing points is computed as:

(30) $$\Sigma_{{\text{GP}}_{p}}=K_{S_{t}S_{t}}-K_{S_{t}P}K_{PP}^{-1}K_{PS_{t}}+K_{S_{t}P}\Sigma_{l}^{-1}K_{PS_{t}},$$

this term accounts for the uncertainty reduction from inducing points.

### Generation module: decoder for reconstructing post-perturbation gene expression.

The posterior GP latent variables and standard Gaussian latent variables are concatenated to form the overall posterior latent distribution:

(31) $$M_{p}=⊕(M_{{\text{GP}}_{p}},M_{\text{Gaussian}}),\;\Sigma_{p}^{2}=⊕(\Sigma_{{\text{GP}}_{p}}^{2},\Sigma_{\text{Gaussian}}^{2}),$$

where $M_{p}$ is the posterior combined mean and $\Sigma_{p}^{2}$ is the posterior combined variance. Then, the latent distribution is modeled as:

(32) $$Z∼𝒩(M_{p},\Sigma_{p}),$$

To enable backpropagation through the latent distribution, the reparameterization trick is used. Latent samples $z_{p}^{k}$ for a spot *k* is drawn as follows:

(33) $$z^{k}=\mu_{p}^{k}+\epsilon \cdot \sigma_{p}^{k},\;\epsilon ∼𝒩(0,I),$$

This process is repeated *g* times to obtain *g* latent samples. The default setting of *g* is 1 in the training stage but 20 in the inference stage for robust predictions.

Each latent sample $z_{p}^{k}$ is passed through the MLP decoder network $f_{d}$ to produce hidden representations:

(34) $$h^{k}=f_{d}(z_{p}^{k}),$$

The decoder outputs are transformed to compute the parameters of the negative binomial (NB) distribution:

(35) $$\text{Mean}\;:\mu_{d}^{k}=\text{exp}(h^{k}),$$

(36) $$\text{Dispersion}\;:\theta_{d}^{k}=\text{exp}(\text{softplus}(h^{k})),$$

In prediction stage, $\frac{1}{g}\sum \mu_{d}^{k}$ is the output of CONCERT standing for the predicted response gene expression of spot *k*.

### Reconstruction Loss.

For each sample *k*, the negative binomial loss is calculated using the raw count data $x_{0}^{k}$, the predicted mean $\mu_{d}^{k}$, the dispersion parameter $\theta_{d}^{k}$, and the size factors $l_{nb}$:

(37) $${\mathrm{NBLoss}}^{\left(k\right)}=-\log\;\mathrm{NB}(x_{0}^{k}\;|\;\mu_{d}^{k},\theta_{d}^{k},l_{nb}^{k}),$$

(38) $$l_{nb}^{k}=\frac{\sum_{g=1}^{G}x_{0g}^{k}}{{\text{median}}_{j}\left(\sum_{g=1}^{G}x_{0g}^{j}\right)},\;k=1,\ldots ,N.$$

where *G* is the total number of genes in $X_{0}$ and *j* is the index over all spots.

The reconstruction loss is averaged across all *N* spots:

(39) $${ℒ}_{\text{recon}}=-\log\;p(X|Z)=\frac{1}{N}\sum_{n=1}^{N}{\mathrm{NBLoss}}^{\left(n\right)}.$$

This reconstruction loss measures the discrepancy between the observed and reconstructed data.

### Objective Function.

The objective function of CONCERT, which is based on the evidence lower bound (ELBO), is given by:

(40) $$\mathrm{ELBO}=-E_{q(z|x,s,𝒞)}[{ℒ}_{\text{recon}}]-\beta \cdot ({\text{KL}}_{\text{GP}}+{\text{KL}}_{\text{Gaussian}})$$

${\text{KL}}_{\text{GP}}$ is the KL divergence for the GP latent variables which incorporates spatial dependencies. ${\text{KL}}_{\text{Gaussian}}$ is the KL divergence for the Gaussian latent variables which incorporates non-spatial information (See details of these KL losses in supplementary Note 1). β is a learnable parameter (initialized at 10) guided by a target value of KL loss which is set to 0.025 in default setting. We then calculate ${\text{ELBO}}_{p}$ as the penalized ELBO by

(41) $${\mathrm{ELBO}}_{p}=\mathrm{ELBO}+\lambda {\left\|w_{s}\right\|}_{2}^{2},$$

where $\lambda {\left\|w_{s}\right\|}_{2}^{2}$ is the L2 regularization for the weights of the basal latent embeddings $E_{s}$, controlled by the hyperparameter λ with default value 1, to constrain it from capturing information already provided by spot attributes, such as perturbations and disease labels.

### Spatial-aware counterfactual prediction for seen and unseen spots.

As shown in Figure 2, CONCERT enables *in silico* perturbation and propagates the perturbation effects across the tissue space. Using Perturb-map data as examples, here we elucidate how CONCERT conducts counterfactual prediction spatially.

In this dataset, an unperturbed tumor spot *k* has embedding $⊕(e_{s}^{k,}=E_{s}[k]$, $e_{d}^{k}=E_{d}[d_{0}]$, $e_{p}^{k}=E_{p}[p_{0}])$. First, if spot *k* is present in the input slide, we replace its current embedding of nonperturbed state ($p_{0}$) with the embedding for a target state caused by the perturbagen $p^{t}$. So its perturbed embedding becomes:

(42) $$e_{p}^{k^{\prime }}=E_{p}[p^{t}]$$

(43) $$⊕(e_{s’}^{k},e_{d}^{k},e_{p}^{k^{\prime }}),$$

The spot and disease embeddings remain unchanged.

Second, if spot *k* is not observed in the input slide, we estimate its spot embedding by averaging the embeddings of its spatial neighbors. If the target attributes for *k*, denoted as $p^{t}$ and $d^{t}$ for perturbation and disease state, respectively, are known, we assign the appropriate embeddings from the relevant codebooks. The perturbed embedding of *k* becomes:

(44) $$e_{d}^{k}=E_{d}[d^{t}],e_{p}^{k}=E_{p}[p^{t}],$$

(45) $$⊕(e_{s’}^{k’},e_{d}^{k},e_{p}^{k}),$$

where $e_{s’}^{k’}$ is the averaged spot embeddings of the spatial neighbors of *k* denoted as 𝒩(k):

(46) $$e_{s’}^{k’}=\frac{1}{|𝒩(k)|}\sum_{j\in 𝒩(k)}e_{s}^{j},$$

Third, if we only want to impute the gene expression of the unseen spot *k* without predicting perturbation, which means we don’t know any attributes of *k*. In this case, for the Gaussian latent variables, we take the average of those from the neighboring spots:

(47) $$\mu_{Gaussian}^{k}=\frac{1}{|𝒩(k)|}\sum_{j\in 𝒩(k)}\mu_{Gaussian}^{j},\;\sigma_{Gaussian}^{k}=\frac{1}{|𝒩(k)|}\sum_{j\in 𝒩(k)}\sigma_{Gaussian}^{j}.$$

For the GP latent variables, we use the prior variables of the observed (seen) spots and their coordinates ($S_{\text{seen}}$), along with the coordinate $S_{k}$ of spot *k*, to approximate the posterior GP latent variables of *k* [26]:

(48) $$\mu_{{\text{GP}}_{p}}^{k},\sigma_{{\text{GP}}_{p}}^{k}=p(\mu_{{\text{GP}}_{q}}^{\text{seen}},\sigma_{{\text{GP}}_{q}}^{\text{seen}},S_{\text{seen}},S_{k}).$$

We then concatenate the posterior GP and Gaussian latent variables as the final latent variables for spot *k*, which is then used for generating the imputed gene expression of it. The kernel cutoff c for the unseen spot is also from its nearest seen neighbors.

### Multi-batch counterfactual prediction.

To account for batch effects in multi-batch (or multislides) input data, we implement three key adjustments in CONCERT. First, we introduce an additional code book during disentanglement to explicitly model batch effects. Second, during reconstruction loss computation, we incorporate batch-specific dispersion vectors to refine the estimation of the Negative Binomial loss. Last, the spot coordinates for each slide is rescaled into the target range separately and then concatenated with the one-hot encoded batch ID vector, enabling us to only consider the spot-spot dependencies within each slide. Notably, in CONCERT, we assume that the propagation pattern of each perturbation remains consistent across slides after correcting the batch effects, so CONCERT learns perturbagen specific kernels, but not batch specific kernels.

### Parameterization and training.

The default dimensionality of the embeddings for the spot attributes is set to 256. The mini-batch size is also set to 256. We employ AdamW [62] as the optimizer, with a learning rate of 1 × 10^−3^ and a weight decay of 1 × 10^−6^. The MLP encoder and decoder architectures are designed with layer configurations of [128, 64] and [128], respectively. The default dimensionalities of the Gaussian Process (GP) latent space and the Gaussian latent space are set to 2 and 8, respectively. For the Gaussian Process module, when multiple perturbagens are present, we use Cauchy kernels with learnable scales for each dimension and each perturbagen. When only a single perturbagen is present, we use a Cauchy kernel with dimension-specific learnable scales that are shared across all spots. The scale values are initialized to 10. The weight of the KL divergence loss (β) is learnable and follows the implementation in spaVAE, targeting a KL loss of 0.025. Five percent of the training set is allocated as the validation set, which is used to monitor the decay of the loss for early stopping. Early stopping is configured with a patience of 200 epochs and a delta threshold of 0. All experiments are conducted on NVIDIA A100 GPUs.

### Implementation on Perturb-Map data.

We conducted patch, border, and niche on Perturb-Map data slides. We sampled 1, 4, and 8 spots in specific patterns that meet the scenario for each task. To do this, we built a Shiny App in R to select spots for CP. To test the robustness of the model performance, we sampled at least 1 pattern per slide per task if possible. Due to the limited number of spots and perturbations on slide GSM5808056, we cannot sample the pattern for Niche CP on it. For the single slide Perturb-map dataset, the perturbation (Jak2-KO, Tgfbr2-KO, Ifgbr2-KO, or control) and spot type (tumor, normal, or periphery) are encoded in the perturbation module. For the multi-slide Perturb-map dataset, an additional variable, slide ID, is also encoded. The single-kernel mode is used in the benchmarking experiments. The top 2,000 highly variable genes are selected for model training.

### Implementation on mouse colon DSS data.

In the case study of mouse colon data, we train CONCERT on all slides collected at different time points before and after perturbation. The attributes include perturbation identity (categorical: DSS or control) and days (continuous: 0, 12, 30, and 73). Top 2,000 highly variable genes are selected for model training. The task is to predict gene expression of a mouse’s colon at missing time points after perturbation.

### Implementation on mouse stroke PT data.

In the case study of mouse stroke data, we train CONCERT on all eight slides, explicitly disentangling the attribute ’stroke’ to capture stroke-specific transcriptomic signatures spatially. The attributes include perturbation identity (PT or control) and slide ID (PT-1 to PT-4 and Sham-1 to Sham-4), which are encoded. The trained model is evaluated by predicting outcomes specifically on the sham samples, ensuring that it effectively distinguishes stroke-associated features while being tested on a non-stroke condition. To investigate the spatial impact of stroke on surrounding cortical regions, we control two experimental variables: 1) Patch size of stroke lesion: this parameter examines how the extent of the ischemic lesion affects adjacent cortical regions and how this effect changes with the size of the lesion. 2) Number of points associated with stroke in the cortex: This variable explores the systematic effects of multi-region stroke, where ischemic damage is dispersed across multiple cortical areas. Adjusting the number of stroke-affected points, we evaluated how the spatial distribution of stroke lesions influences transcriptomic patterns. We conducted these tests with 2D locations of spots. For the data in 3D space, we adjust CONCERT for using 3D Cauchy kernels to learn the dispersion of perturbation effects on 3D space (Supplementary Note 5). Then we train CONCERT using the PT slides and predict perturbation on the sham slides. The top 2,000 highly variable genes are selected for model training. The details of the implementation of CONCERT in the three-dimensional tissue space are described in the Supplementary Note 5.

## Methods used in benchmarking analyses

We consider three types of competing methods to benchmark CONCERT:

1. **kNN-based methods**: This includes kNN approaches that find neighbors based on spatial distance (kNN-SP) or transcriptomic distance (kNN-GEX) among spots. Notably, when perturbing a spot to a target perturbation state, neighbors are selected specifically from spots within the target state rather than the closest spots on the tissue. In many scenarios, kNN is considered the upper-bound performance, as its methodology aligns closely with the approach used to generate the ground truth.
2. **Existing counterfactual prediction (CP) methods**: These include Biolord [23], scGEN [19], and CPA [20]. All parameters are kept at their default values for these methods. Biolord is run under two settings:
  - Using only gene expression (GEX) as input, without considering spatial information.
  - Dividing the tissue into *L* = 5 × 5 patches and using the patch ID as input to account for spatial location (denoted as Biolord-SP).
3. **Modified existing/non-spatial CP methods by incorporating spatial-aware module**: Based on approaches commonly used for spatial transcriptomics (ST) data [25, 28], we employ graph neural networks (GNNs) to capture dependencies among spots based on their spatial locations. A kNN graph (*k* = 6) is used to construct edges for the graph. The multilayer perceptron (MLP) encoder in scGEN, chemCPA, and Biolord is replaced with a three-layer graph convolution network. The first layer employs a Graph Convolutional Network (GCN) for initial spatial feature extraction, followed by two GCN layers for spatial representation processing. This structure allows the model to capture both local and broader spatial patterns in the tissue. These updated models are referred to as scGEN-GCN, CPA-GCN, and Biolord-GCN, respectively. We did not include Celcomen as a competing method due to the inability to reproduce its counterfactual prediction on spatial perturbation datasets based on the provided code.

These methods establish a strong benchmarking framework, allowing a robust comparison of CONCERT against different methodological approaches.

## Evaluation

The selection of spots with the target state for evaluation the ”ground truth” spot, varies by task. Our evaluation criterion requires that these spots belong to the same niche as the perturbed spots (source spots). For each task, we sample the spots for prediction where the ”ground truth” spots in the target state are present. For within-niche tasks, we sample spots for prediction where the ”ground truth” spots are present in the same local niche. For cross-niche tasks, we select spots where the ”ground truth” spots are not in proximity but are in a similar niche, defined by comparable compositions of surrounding spot types. For border tasks, we sample spots located on the boundary of a patch, ensuring that the ”ground truth” spots also lie along the same boundary. For niche tasks, we select a patch in state *a* within niche *A* and perform an *in-silico* perturbation of its surrounding niche from *A* to *B*. For evaluation, we ensure that another patch in state *a* naturally exists within niche *B* in the original data.

We calculate *R*^2^, Pearson correlation coefficient (PCC), mean absolute error (MAE), and E-distance between the ’ground truth’ and predicted expression of genes for evaluation. These metrics are defined as:

(49) $$R^{2}=1-\frac{\sum_{i=1}^{g}{\left(y_{i}-x_{i}\right)}^{2}}{\sum_{i=1}^{g}{\left(y_{i}-\bar{y}\right)}^{2}}$$

(50) $$\text{PCC}=\frac{\sum_{i=1}^{g}(x_{i}-\bar{x})(y_{i}-\bar{y})}{\sqrt{\sum_{i=1}^{g}{\left(x_{i}-\bar{x}\right)}^{2}}\sqrt{\sum_{i=1}^{g}{\left(y_{i}-\bar{y}\right)}^{2}}}$$

(51) $$\text{MAE}=\frac{1}{g}\sum_{i=1}^{g}|y_{i}-x_{i}|$$

(52) $$\text{Edist}(X,Y)=\frac{2}{NM}\sum_{i=1}^{N}\sum_{j=1}^{M}\|x_{i}-y_{j}\|-\frac{1}{N^{2}}\sum_{i=1}^{N}\sum_{j=1}^{N}\|x_{i}-x_{j}\|-\frac{1}{M^{2}}\sum_{i=1}^{M}\sum_{j=1}^{M}\|y_{i}-y_{j}\|$$

where $x_{i}$ denotes the expression of the predicted response gene for the spot $i;y_{i}$ is the true expression of a randomly paired target spot corresponding to $i;\bar{x}$ and $\bar{y}$ represents the mean value of $y_{i}$ and $x_{i};g$ is the number of predicted gene; X and Y are the patches of the predicted and target spots; *N* and *M* are the sizes of X and Y. Note that when the patch size is greater than 1, the metrics *R*^2^, PCC, and MAE are computed using the averaged predictions of spots, whereas E-distance is computed using the patch-level predictions. For better visualization, the calculated E-distance values were log-transformed. To integrate the performance across multiple tests, we used an average rank score calculated as follows: 1) rank the methods in each test by the metrics mentioned above from worst to best; 2) calculate the mean value of the ranks for each method; 3) use the reciprocal of the averaged ranks as the final score.

To evaluate the performance of spot imputation, we randomly masked *k* spots (*k* = 10, 20, 40 and 80) using two sampling strategies: (1) completely random selection of *k* spots throughout the tissue and (2) random selection of *k* spots specifically from boundary areas of patches (such as a tumor patch). A spot is classified as being in a boundary area if the most dominant spot type among its 6 neighbors constitutes less than 50%. For benchmarking, BioLORD-GCN was used since it can also do data imputation. All experiments were performed on the Perturb-Map data GSM5808054.

## Sensitivity analyses

We tuned seven hyper-parameters of CONCERT to test the robustness of it: 1) encoders with 1~4 layers; 2) encoders with 1~4 layers; 3) dimensionality of GP latent layer {2, 4, 6, 8, 10}; 4) dimensionality of Gaussian latent layer {2, 4, 6, 8, 10}; 5) kernel scale of GP {5, 10, 20, 30, 40}; 6) target KL loss value {0.01, 0.025, 0.05, 0.1, 0.25, 0.5, 1.0}; 7) range of rescaled spot coordinates {10, 20, 30, 40}. We tested CONCERT for a within-niche and a Border CP task with various testing patch sizes (1, 4, and 8 spots). For each configuration, we train and test CONCERT five times to get robust results. Performance is also evaluated by *R*^2^ between the predicted GEX of the perturbed spots and the true GEX of the spots in the target perturbation state (e.g., Jak2-KO).

We then tested the sensitivity of CONCERT’s performance on the training sample size. We randomly sampled 0.1, 0.2, and 0.3 ratios of spots in each spot type (the annotation provided in the original paper [12]) and removed them from the training set. We repeated this approach three time with different random seeds. This experiment is conducted on sample GSM5808054, GSM5808055, GSM5808056, and GSM5808057 for the within-niche patch task with various testing patch sizes (1, 4, and 8 spots). *R*^2^ is used to evaluated the performance.

## Statistical analyses

In benchmarking experiments, one-sided T-tests were performed between CONCERT and each competing method, using a significance threshold of *p* < 0.05. Based on the outputs of CONCERT, we performed a series of downstream bioinformatics analyses. For differential expression (DE) analysis, we applied two-tailed Wilcoxon rank-sum tests to compare normalized predicted gene expression counts between perturbed and control spots. The control group consisted of normal spots within the tissue. For gene set enrichment analysis (GSEA), we used the R package fgsea [63] with Hallmark gene sets from the MSigDB database [64]. Log-fold changes derived from the DE analysis were used to rank genes for enrichment testing. Single-sample GSEA (ssGSEA) was performed using the R package GSVA [65], with the same collection of gene sets, to calculate enrichment scores at the individual spot level.

## Extended Data

**Extended Data Fig. 2:**
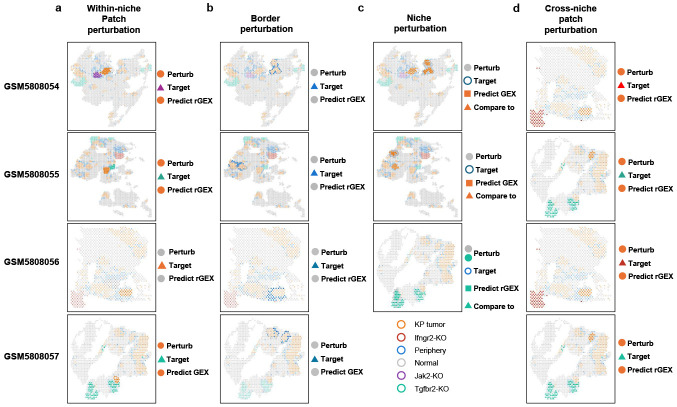
Spots sampled for benchmarking spatial perturbation experiments. We sampled the spots for patch (within-niche, a), border (b), niche (c), and patch (cross-niche, d) tasks on Perturb-map slides GSM580805, GSM5808055, GSM5808056, and GSM5808057. We didn’t sample spots for niche perturbation on GSM5808056 since the lack of target patches for evaluation. Perturbing spots (Perturb) refer to spots subjected to counterfactual prediction (CP) experiments; Target spots (Target) are the spots carrying the target cell state (e.g. gene expression post perturbation). rGEX indicates the response gene expression (post-perturbation gene expression).

**Extended Data Fig. 3:**
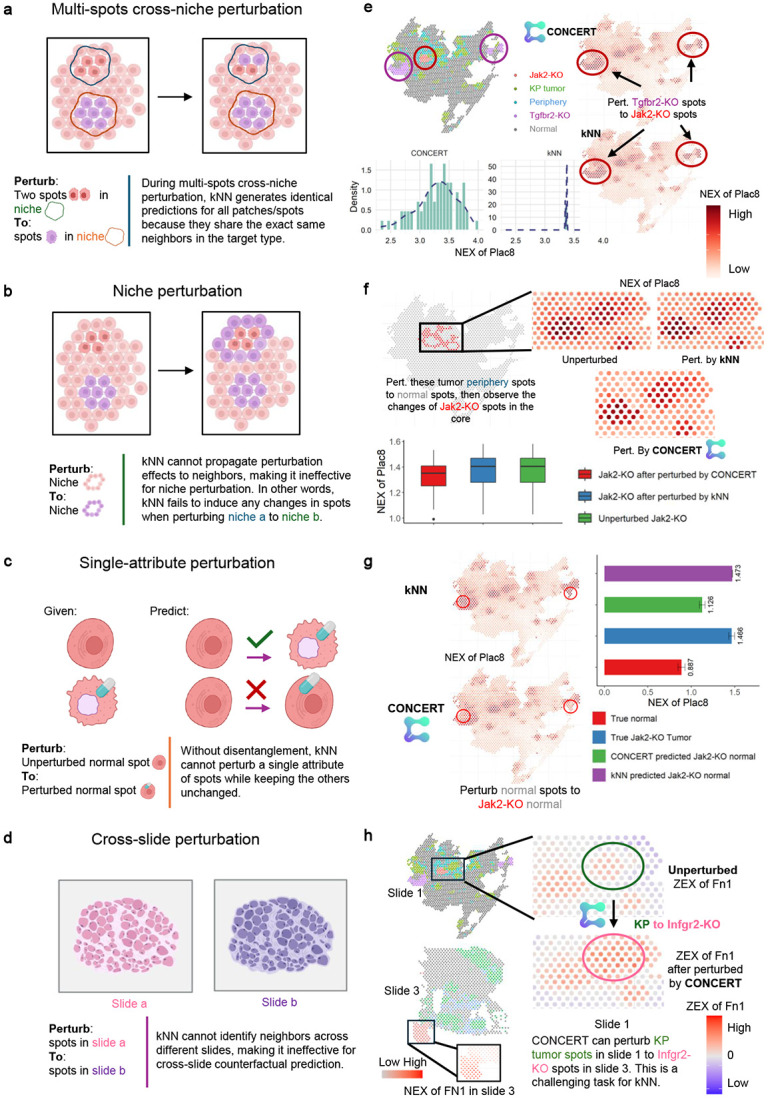
Comparison of CONCERT and kNN across four challenging CP Scenarios. (a-d) Limitations of kNN methods: kNN relies on nearest neighbors for predictions, leading to uniform outputs with distant target spots (a), inability to model spatial decay effects (b), failure to disentangle multiple attributes (c), and inability to integrate data across samples (d). (e-h) Superior performance of CONCERT: In scenario a, CONCERT captures spatial patterns, accurately predicting Plac8 expression in tumor cores and surfaces (e). In scenarios b, CONCERT models spatial decay, reflecting biological changes when tumor periphery spots are perturbed to normal spots (f). In scenarios c, CONCERT disentangles attributes, predicting low Plac8 expression in Jak2-KO normal spots (g). In scenarios d, CONCERT predict perturbation across samples (h). NEX: Normalized gene expression; ZEX: Z-score of normalized expression.

**Extended Data Fig. 4:**
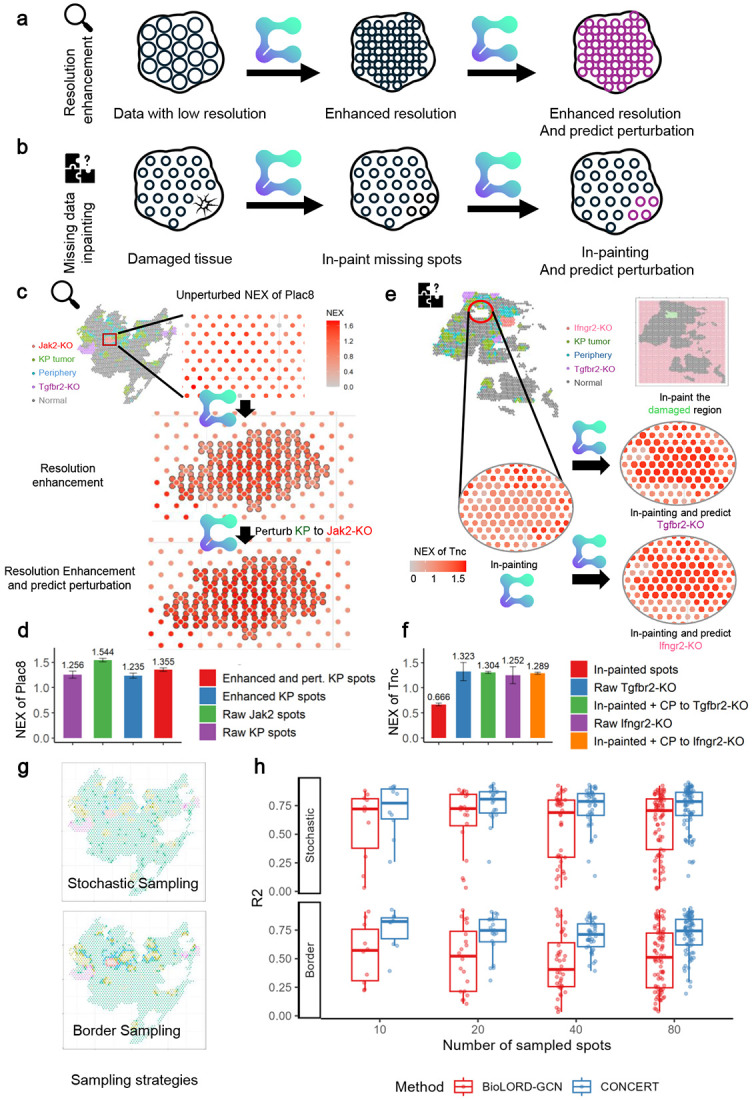
CONCERT enables resolution enhancement and missing-data imputation through counterfactual prediction (CP). (a-b) CONCERT enhances resolution and imputes missing data by adding extra spots in blank regions of slides and estimating their gene expression based on spatial dependencies with neighboring spots. It can then perturb the attributes of these imputed cells/spots and predict their post-perturbation gene expression. (c) Example of resolution enhancement: CONCERT imputes gene expression for KP tumor spots and then perturbs them into Jak2-KO spots. (d) The imputed KP tumor spots and their perturbed Jak2-KO counterparts exhibit gene expression profiles closely matching those of true KP and Jak2-KO spots. (e) Example of missing-data imputation: CONCERT imputes missing spots and then perturbs them into Tgfbr2-KO and Ifngr2-KO spots. (f) The imputed Tgfbr2-KO and Ifngr2-KO spots display gene expression patterns similar to their true counterparts. (g) Experimental setup for evaluating imputation performance using two sampling strategies: completely stochastic sampling and border spot sampling. Border spots represent more heterogeneous environments, making them particularly challenging to impute. Additionally, spots on borders often form patches, aligning with typical missing-data imputation scenarios.(h) Comparison of *R*^2^ performance between CONCERT and bioLORD-GCN, a generative model capable of CP and imputation. CONCERT consistently outperforms bioLORD-GCN across both sampling strategies and varying numbers of masked spots. While bioLORD-GCN’s performance drops significantly for border spots, CONCERT remains robust and demonstrates superior overall performance. NEX: Normalized gene expression.

**Extended Data Fig. 5:**
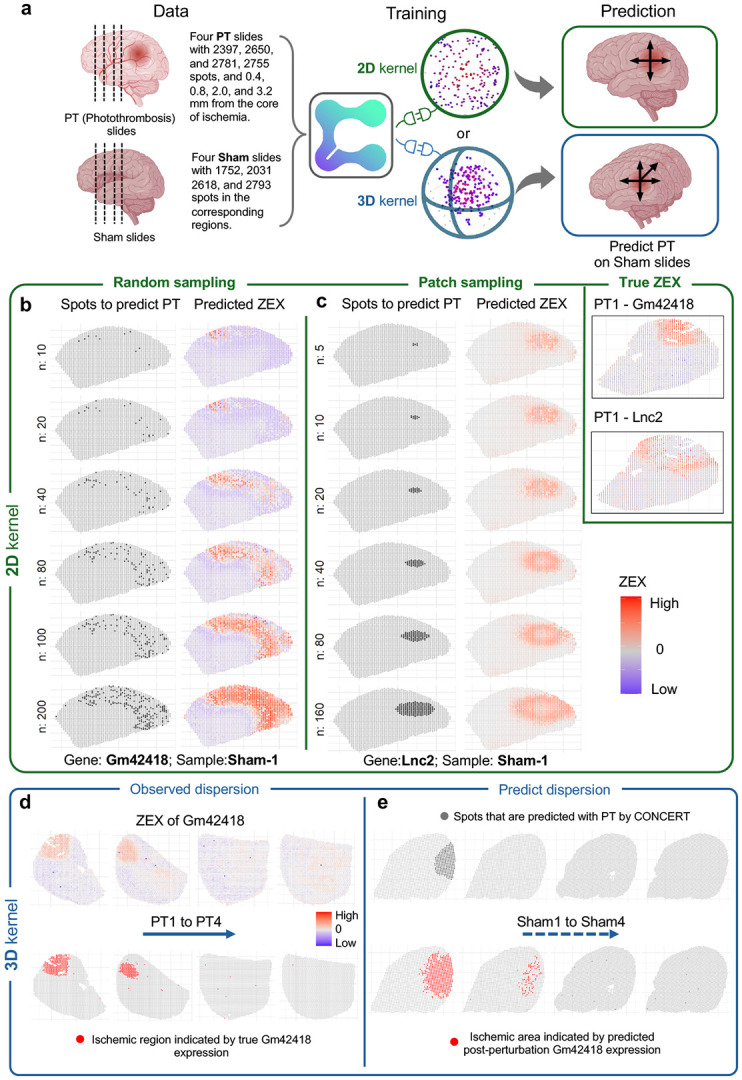
CONCERT enables *in-silico* photothrombosis (PT) surgery on healthy (sham) brain slides, allowing precise control over ischemic region size and location while propagating perturbation effects in 2D and 3D tissue spaces. (a) Given brain PT and sham slides, CONCERT can simulate PT perturbation on sham slides and propagate its effects across 2D and/or 3D space using corresponding kernels. (b-c) Experimental setup using a 2D kernel: Two sampling strategies were applied: (b) random sampling and (c) patch sampling, with controlled spot numbers (patch sizes). The right columns in panels B and C show the predicted post-perturbation expression of two ischemia marker genes identified in the original dataset: Gm42418 (a marker of the core ischemic region) and Lcn2 (a marker of the peripheral ischemic region). (d-e) Predicted dispersion of PT effects in 3D tissue space: (d) Observed spread of PT effects from slide PT1 to PT4. (e) *In-silico* perturbation of a spot patch on Sham 1 (top), with the predicted dispersion of PT effects across PT1 to PT2-4 (bottom). The ischemic areas are identified by the upregulation of Gm42418, determined by comparing its expression before and after perturbation on each sham slide. ZEX indicates the Z-score of normalized gene expression.

**Extended Data Fig. 6:**
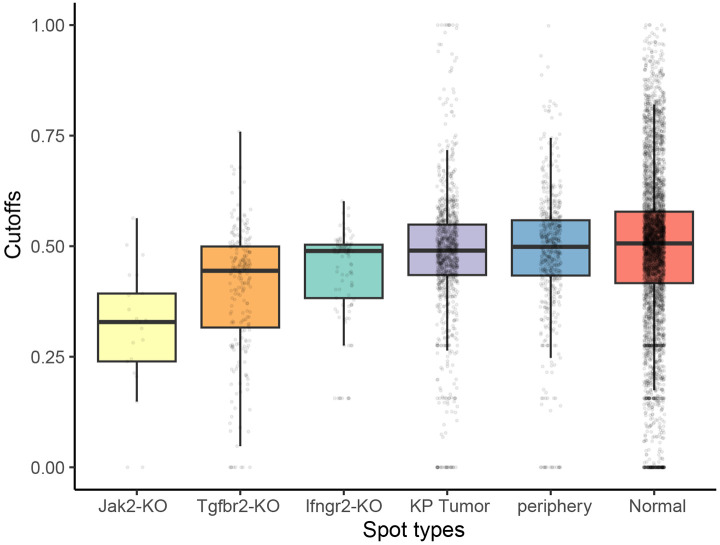
The learned spot-specific kernel cutoffs by CONCERT for all Perturb-Map datasets, GSM5808054, GSM5808055, GSM5808056, and GSM5808057, categorized by the spot type.

## Supplementary Material

Supplement 1

## Data Availability

The raw Perturb-map dataset is available via GEO under accession number GSE193460. The processed data is available via Figshare at https://figshare.com/articles/dataset/Datasets_-_Perturb-Map/29198468. The raw mouse gut DSS dataset used in the case study is available through the Broad Institute Single Cell Portal under accession number SCP2771. The processed data is available via Figshare at https://figshare.com/articles/dataset/Mouse_gut_inflammation_dataset/29882873. The raw mouse brain stroke dataset is available from the authors of the original paper [42] upon request. The processed data is available via Figshare at https://figshare.com/articles/dataset/Mouse_brain_stroke_dataset/29882900.

## Code availability.

Project website is at http://zitniklab.hms.harvard.edu/projects/CONCERT. Python implementation of CONCERT is at https://github.com/mims-harvard/CONCERT.
