# Supplementary material for: CONCERT predicts niche-aware perturbation responses in spatial transcriptomics
Source: bioRxiv. 2025 Nov 20:2025.11.08.686890. Preprint. [Version 2] doi: 10.1101/2025.11.08.686890 (PMC12642540; doi:10.1101/2025.11.08.686890)
Supplement: Supplement 1 [file media-1.pdf]

Supplementary Information for

CONCERT predicts niche-aware perturbation responses in spatial transcriptomics

Xiang Lin[1], Zhenglun Kong[1], Soumya Ghosh[2], Manolis Kellis[4,6], Marinka Zitnik[1,3,4,5,‡]

[1]Department of Biomedical Informatics, Harvard Medical School, Boston, MA, USA

[2]Merck & Co., Inc., Cambridge, MA, USA

[3]Kempner Institute for the Study of Natural and Artificial Intelligence, Harvard University, Cambridge, MA, USA

[4]Broad Institute of MIT and Harvard, Cambridge, MA, USA

[5]Harvard Data Science Initiative, Cambridge, MA, USA

[6]Computer Science and Artificial Intelligence Laboratory, MIT, Cambridge, MA, USA

[‡]Correspondence: marinka@hms.harvard.edu

Supplementary Notes

- **Supplementary Note 1**. Details of loss functions.
- **Supplementary Note 2**. Sensitivity analyses of CONCERT.
- **Supplementary Note 3**. Novel tasks designed for spatial perturbation data.
- **Supplementary Note 4**. CONCERT with three dimensional kernel.

Supplementary Tables (in separated Excel files)

- **Supplementary Table 1**. P-values of the comparisons between CONCERT and all competing methods in benchmarking experiments of patch perturbation task on Perturb-map datasets evaluated by E-distance.
- **Supplementary Table 2**. P-values of the comparisons between CONCERT and all competing methods in benchmarking experiments of patch perturbation task on Perturb-map datasets evaluated by mean absolute error.
- **Supplementary Table 3**. P-values of the comparisons between CONCERT and all competing methods in benchmarking experiments of patch perturbation task on Perturb-map datasets evaluated by Pearson correlation coefficient.
- **Supplementary Table 4**. P-values of the comparisons between CONCERT and all competing methods in benchmarking experiments of patch perturbation task on Perturb-map datasets evaluated by R^2.

- **Supplementary Table 5**. P-values of the comparisons between CONCERT and all competing methods in benchmarking experiments of border perturbation task on Perturb-map datasets evaluated by E-distance.
- **Supplementary Table 6**. P-values of the comparisons between CONCERT and all competing methods in benchmarking experiments of border perturbation task on Perturb-map datasets evaluated by mean absolute error.
- **Supplementary Table 7**. P-values of the comparisons between CONCERT and all competing methods in benchmarking experiments of border perturbation task on Perturb-map datasets evaluated by Pearson correlation coefficient.
- **Supplementary Table 8**. P-values of the comparisons between CONCERT and all competing methods in benchmarking experiments of border perturbation task on Perturb-map datasets evaluated by R^2.
- **Supplementary Table 9**. P-values of the comparisons between CONCERT and all competing methods in benchmarking experiments of niche perturbation task on Perturb-map datasets evaluated by E-distance.
- **Supplementary Table 10**. P-values of the comparisons between CONCERT and all competing methods in benchmarking experiments of niche perturbation task on Perturb-map datasets evaluated by mean absolute error.
- **Supplementary Table 11**. P-values of the comparisons between CONCERT and all competing methods in benchmarking experiments of niche perturbation task on Perturb-map datasets evaluated by Pearson correlation coefficient.
- **Supplementary Table 12**. P-values of the comparisons between CONCERT and all competing methods in benchmarking experiments of niche perturbation task on Perturb-map datasets evaluated by R^2.
- **Supplementary Table 13**. P-values of the comparisons between CONCERT and all competing methods in benchmarking experiments of cross-niche patch perturbation task on Perturb-map datasets evaluated by E-distance.
- **Supplementary Table 14**. P-values of the comparisons between CONCERT and all competing methods in benchmarking experiments of cross-niche patch perturbation task on Perturb-map datasets evaluated by mean absolute error.
- **Supplementary Table 15**. P-values of the comparisons between CONCERT and all competing methods in benchmarking experiments of cross-niche patch perturbation task on Perturb-map datasets evaluated by Pearson correlation coefficient.
- **Supplementary Table 16**. P-values of the comparisons between CONCERT and all competing methods in benchmarking experiments of cross-niche patch perturbation task on Perturb-map datasets evaluated by R^2.

Supplementary Figures

- **Supplementary Figure 1**. Problem formation of CONCERT.
- **Supplementary Figure 2**. Mechanisms of the dispersion of perturbation effects.
- **Supplementary Figure 3**. Disentanglement of the latent space of CONCERT.

- **Supplementary Figure 4**. Annotated slide GSM5808054 and GSM5808055.
- **Supplementary Figure 5**. Annotated slide GSM5808056 and GSM5808057.
- **Supplementary Figure 6**. Benchmarking experiments of patch perturbation task on Perturb-map datasets evaluated by mean absolute error.
- **Supplementary Figure 7**. Average ranks of patch perturbation task on Perturb-map datasets evaluated by mean absolute error.
- **Supplementary Figure 8**. Benchmarking experiments of patch perturbation task on Perturb-map datasets evaluated by Pearson correlation coefficient.
- **Supplementary Figure 9**. Average ranks of patch perturbation task on Perturb-map datasets evaluated by Pearson correlation coefficient.
- **Supplementary Figure 10**. Benchmarking experiments of patch perturbation task on Perturb-map datasets evaluated by R^2.
- **Supplementary Figure 11**. Average ranks of patch perturbation task on Perturb-map datasets evaluated by R^2.
- **Supplementary Figure 12**. Benchmarking experiments of border perturbation task on Perturb-map datasets evaluated by mean absolute error.
- **Supplementary Figure 13**. Average ranks of border perturbation task on Perturb-map datasets evaluated by mean absolute error.
- **Supplementary Figure 14**. Benchmarking experiments of border perturbation task on Perturb-map datasets evaluated by Pearson correlation coefficient.
- **Supplementary Figure 15**. Average ranks of border perturbation task on Perturb-map datasets evaluated by Pearson correlation coefficient.
- **Supplementary Figure 16**. Benchmarking experiments of border perturbation task on Perturb-map datasets evaluated by R^2.
- **Supplementary Figure 17**. Average ranks of border perturbation task on Perturb-map datasets evaluated by R^2.
- **Supplementary Figure 18**. Benchmarking experiments of niche perturbation task on Perturb-map datasets evaluated by mean absolute error.
- **Supplementary Figure 19**. Average ranks of niche perturbation task on Perturb-map datasets evaluated by mean absolute error.
- **Supplementary Figure 20**. Benchmarking experiments of niche perturbation task on Perturb-map datasets evaluated by Pearson correlation coefficient.
- **Supplementary Figure 21**. Average ranks of niche perturbation task on Perturb-map datasets evaluated by Pearson correlation coefficient.
- **Supplementary Figure 22**. Benchmarking experiments of niche perturbation task on Perturb-map datasets evaluated by R^2.

- **Supplementary Figure 23**. Average ranks of niche perturbation task on Perturb-map datasets evaluated by R^2.
- **Supplementary Figure 24**. Benchmarking experiments of cross-niche patch perturbation task on Perturb-map datasets evaluated by mean absolute error.
- **Supplementary Figure 25**. Average ranks of cross-niche patch perturbation task on Perturb-map datasets evaluated by mean absolute error.
- **Supplementary Figure 26**. Benchmarking experiments of cross-niche patch perturbation task on Perturb-map datasets evaluated by Pearson correlation coefficient.
- **Supplementary Figure 27**. Average ranks of cross-niche patch perturbation task on Perturb-map datasets evaluated by Pearson correlation coefficient.
- **Supplementary Figure 28**. Benchmarking experiments of cross-niche patch perturbation task on Perturb-map datasets evaluated by R^2.
- **Supplementary Figure 29**. Average ranks of cross-niche patch perturbation task on Perturb-map datasets evaluated by R^2.
- **Supplementary Figure 30**. Uncertainty of prediction on within niche CP task.
- **Supplementary Figure 31**. Results of GESA and ssGESA for case stduy 1.
- **Supplementary Figure 32**. Learned kernel values in CONCERT for four example spots from Perturb-map data slide GSM5808054.
- **Supplementary Figure 33**. Imputed data of mouse gut for the missing time-points visualized by gene Clca4b.
- **Supplementary Figure 34**. Positive and negative inflamed spots identified by using cutoff quantile 0.85 of marker gene Clca4b.
- **Supplementary Figure 35**. Imputed data of mouse gut for the missing time-points visualized by gene Ido1.
- **Supplementary Figure 36**. Positive and negative inflamed spots identified by using cutoff quantile 0.85 of marker gene Ido1.
- **Supplementary Figure 37**. Imputed data of mouse gut for the missing time-points visualized by gene Il1b.
- **Supplementary Figure 38**. Positive and negative inflamed spots identified by using cutoff quantile 0.85 of marker gene Il1b.
- **Supplementary Figure 39**. Imputed data of mouse gut for the missing time-points visualized by gene Il11.
- **Supplementary Figure 40**. Positive and negative inflamed spots identified by using cutoff quantile 0.85 of marker gene Il11.
- **Supplementary Figure 41**. Normalized expression of the marker gene Gm42418 indicating the core ischemic region.

- **Supplementary Figure 42**. Normalized expression of the marker gene Supp1 indicating the periphery ischemic region
- **Supplementary Figure 43**. Normalized expression of the marker gene Lnc2 indicating the periphery ischemic region.
- **Supplementary Figure 44**. Predicted response expression of marker gene Gm42418 to in-silico PT perturbation on stochastically sample spots.
- **Supplementary Figure 45**. Predicted response expression of marker gene Supp to in-silico PT perturbation on stochastically sample spots.
- **Supplementary Figure 46**. Predicted response expression of marker gene Lnc2 to in-silico PT perturbation on stochastically sample spots.
- **Supplementary Figure 47**. Aligned PT and Sham slides in mouse stroke dataset for build 3D coordinate system.
- **Supplementary Figure 48**. Neighbors within and cross samples when using the 3D coordinates for mouse stroke dataset.
- **Supplementary Figure 49**. Sensitivity analyses of CONCERT.
- **Supplementary Figure 50**. Sensitivity analyses to test how CONCERT's performance depends on training dataset size.

Supplementary Notes

Note 1: Details on loss functions in CONCERT

The KL divergence terms for the Gaussian Process (GP) and Gaussian embeddings are key components of ELBO (Evidence Lower Bound) in CONCERT.

KL divergence loss for Gaussian process. The KL loss for the GP embeddings is:

$$\mathrm{KL}_{\mathrm{GP}} = \mathrm{CE}\big(\mathcal{N}(\mathbf{M}, \mathbf{B}) \,\|\, \mathcal{N}(\tilde{\boldsymbol{W}}_{1:\mathrm{L}}, \tilde{\boldsymbol{\Phi}}^2_{1:\mathrm{L}})\big) + \frac{\mathrm{b}}{\mathrm{N}}\mathrm{L}_H,$$

where:

- $\mathrm{CE}(\cdot \,\|\, \cdot)$: Cross-entropy between the Gaussian posterior and prior,
- $\mathbf{M}, \mathbf{B}$: Mean and covariance of the posterior
- $\tilde{\mathbf{W}}_{1:\mathrm{L}}$, $\tilde{\boldsymbol{\Phi}}^2_{1:\mathrm{L}}$: Parameters of the GP prior, where $\tilde{\mathbf{W}}_{1:\mathrm{L}} = 0$ and $\tilde{\boldsymbol{\Phi}}^2_{1:\mathrm{L}} = k(\cdot, \cdot)$ - a kernel function.
- L: Dimensionality of GP latent space, which is set to 2 as default.
- L_H: ELBO of the GP regression marginal likelihood
- b: Mini-batch size.
- N: Total training points.

KL divergence loss for Gaussian embeddings. The KL loss for the Gaussian embeddings is:

$$\mathrm{KL}_{\mathrm{Gaussian}} = \frac{1}{2}\sum_{l=\mathrm{L}+1}^{\mathrm{D}} \left[\log \tilde{\boldsymbol{\phi}}^2_l - \tilde{\boldsymbol{\phi}}^2_l - \tilde{\mathbf{w}}^2_l + 1\right],$$

where:

- $\tilde{\boldsymbol{\phi}}^2_l$: Variance of the posterior for latent Gaussian dimension l
- $\tilde{\mathbf{w}}_l$: Mean of the posterior for latent Gaussain dimension l
- l: Dimension index for Gaussian embedding.
- D: total dimensionality of the embedding, which is set to 10 as default.

Gaussian process posterior. Using $\mathbf{S}$ and $\mathbf{X}$ indicate the location and gene expression of spots, respectively, the posterior distribution f_* for a new input with $\mathbf{S}_*$ given training data $(\mathbf{S}, \mathbf{X})$ and a kernel $k(\cdot, \cdot)$ is:

$$p(f_* \mid \mathbf{S}_*, \mathbf{S}, \mathbf{X}) = \mathcal{N}(\mathbf{M}_{\mathrm{posterior}}, \boldsymbol{\Sigma}^2_{\mathrm{posterior}}),$$

where:

$$\mathbf{M}_{\mathrm{posterior}} = k(\mathbf{S}_*, \mathbf{S})k^{-1}_{\mathrm{noisy}}\mathbf{X},$$

$$\boldsymbol{\Sigma}^2_{\mathrm{posterior}} = k(\mathbf{S}_*, \mathbf{S}_*) - k(\mathbf{S}_*, \mathbf{S})k^{-1}_{\mathrm{noisy}}k(\mathbf{S}_*, \mathbf{S})^\top,$$

and the noisy covariance matrix is:

$$k_{\mathrm{noisy}} = k(\mathbf{S}, \mathbf{S}) + \boldsymbol{\Sigma}^2_n\mathbf{I},$$

where $\mathbf{\Sigma}_n^2$ is the added noise variance. It is noted that $\mathbf{S}$ and $\mathbf{S}_*$ are the same in training stage but different when we want to impute any unseen spots.

The Gaussian posterior of a new input with $\mathbf{S}_*$ is from the $\mathbf{X}$ of its existing neighbors with $\mathbf{S}$. The encoder maps input $\mathbf{X}$ to the parameters of the posterior distribution:

$$q(z \mid \mathbf{X}) = \mathcal{N}(\boldsymbol{W}, \mathbf{\Phi}^2),$$

where $\boldsymbol{W}$ and $\mathbf{\Phi}^2$ are outputs of the encoder from dimension L $+\ 1$ to D. The L and D are the dimensionalities of GP and total (GP + Gaussian) latent space, respectively.

Gaussian process regression. The marginal likelihood of a Gaussian Process (GP) regression model quantifies the probability of observing data given the model parameters. It is expressed as:

$$p(\mathbf{X} \mid \mathbf{S}, \theta) = \frac{1}{(2\pi)^{N/2}|k_{\text{noisy}}|^{1/2}} \exp\left(-\frac{1}{2}\mathbf{X}^\top k_{\text{noisy}}^{-1}\mathbf{X}\right),$$

where θ are the kernel hyperparameters, e.g. the scale per dimensionality for each perturbation. So, the log marginal likelihood is:

$$\log p(\mathbf{X} \mid \mathbf{S}, \theta) = -\frac{1}{2}\mathbf{X}^\top k_{\text{noisy}}^{-1}\mathbf{X} - \frac{1}{2}\log|k_{\text{noisy}}| - \frac{N}{2}\log 2\pi.$$

Note 2: Sensitivity analyses of CONCERT

To evaluate the robustness of CONCERT, we conducted a comprehensive sensitivity analysis by tuning seven key hyperparameters: (1) the number of encoder layers (1–4), (2) the number of decoder layers (1–4), (3) the dimensionality of the GP latent layer (2, 4, 6, 8, 10), (4) the dimensionality of the Gaussian latent layer (2, 4, 6, 8, 10), (5) the kernel scale of the GP (5, 10, 20, 30, 40), (6) the target KL divergence value (0.01, 0.025, 0.05, 0.1, 0.25, 0.5, 1.0), and (7) the rescaled coordinate range of spot locations (10, 20, 30, 40) (Supplementary Fig. 49). We tested CONCERT on two novel tasks defined above, within-niche patch CP and Border CP, with evaluation performed at varying numbers of spots (1, 4, and 8). For each configuration, we trained and tested the model five times to ensure consistent and reliable predictions. Model accuracy was assessed using the R^2 score between the predicted gene expression (GEX) of perturbed spots and the true GEX under the target state (e.g., Jak2-KO).

The results showed that CONCERT maintains stable performance across all tested hyperparameter settings. In the within-niche patch CP task, R^2 values remained consistent across different configurations. Similarly, in the more challenging Border CP task, CONCERT consistently achieved high R^2 scores regardless of hyperparameter choices. Specifically, variations in encoder/decoder depth (1–4 layers) and latent dimensionality (2–10) had minimal effect on performance, indicating that CONCERT balances model complexity and predictive power effectively.

Changes in the initial GP kernel scale and KL loss target also had negligible influence on prediction accuracy, suggesting the model's flexibility in adapting to varying starting points of scales and regularization levels. Likewise, the rescaled spot coordinate range did not significantly affect outcomes, confirming that CONCERT is robust to changes in spatial resolution. Across all settings and testing patch sizes (1, 4, and 8 spots), CONCERT demonstrated consistent performance, highlighting its ability to capture spatial dependencies at different spatial scales.

We also evaluated the sensitivity of CONCERT 's performance to training set size using the within-niche patch CP task on samples GSM5808054, GSM5808055, GSM5808056, and GSM5808057. Performance is evaluated by E-distance (lower value shows better performance). Specifically, 10%, 20%, and 30% of spots were randomly removed from the training sets. Across all settings and testing patch sizes (1, 4, and 8 spots), CONCERT consistently demonstrated robust performance in three (GSM5808055, GSM5808056, and GSM5808057) out of four samples, highlighting its low dependency on training sample sizes (Supplementary Fig. 50).

In summary, this sensitivity analysis confirms that CONCERT is highly robust to hyperparameter variation, maintaining reliable performance across diverse configurations. It also maintain a stable performance over different training sample sizes. This stability ensures practical utility, especially in real-world scenarios where datasets are with various types of perturbations, and from different tissues and technologies with various resolution.

Note 3: Novel tasks designed for spatial perturbation data

For task definition, we refer to each data entry as a cell, which may correspond to a spot in the real data. With spatial information, the traditional counterfactual prediction of response gene expression (rGEX) of each cell can be extended to several novel tasks (Extended Data Fig, E1). First, with knowing the location of one or multiple cells in tissue, we can predict its rGEX to perturbation observed in the same slide - defined as seen perturbagens, or unseen in that slide but observed in different slides - defined as unseen perturbagen. This task facilitates the exploration of cellular responses to a broader range of perturbations by aggregating perturbagens observed across multiple tissue slides. Besides, beyond predicting rGEX of a single cell, we can also do it simultaneously for several spatially related cells, making the experiments more biological meaningful. Here, we also defined three multi-cell tasks. The first task is Patch CP, where we perturb a group of spatially close cells (a patch) instead of individual cells. This task is crucial because many cell types, such as cancer cells, tend to cluster in tissue patches, and the cells within a patch are highly interdependent. Predicting the rGEX of an entire patch while accounting for cell-cell dependencies provides a more realistic and biologically meaningful analysis compared to predicting rGEX for individual cells in isolation. This task has two sub-tasks where the cells with target state are located in the same or different niches with the cells to perturb (source cells), defined as within-niche Patch CP and cross-niche Patch CP. The second task is Border CP, which involves predicting rGEX of cells located on the boundary between two patches of different cell types. Border CP is essential because border cells, such as tumor periphery cells, often exhibit distinct functions compared to cells in the core of a patch. However, this task is challenging due to the narrowness of border regions and the complex influence of neighboring cells on both sides. The third task is Niche CP, which focuses on *in-silico* perturbing the surrounding cells (niche) of a specific cell or cell group to predict their rGEX. This task is particularly significant in tumor research, where the niche can profoundly affect tumor growth, immune evasion, angiogenesis, and response to therapies. For example, perturbing the tumor microenvironment (TME) can help uncover mechanisms of tumor-immune interactions and identify vulnerabilities that could inform therapeutic strategies. Despite the importance of these tasks, current CP methods are limited to predicting GEX responses for individual cells independently and fail to address these spatially complex scenarios.

Note 4: CONCERT with three dimensional kernel

When the inputs are aligned slide deck, CONCERT uses three dimensional (3D) Cauchy kernel to capture the spot-spot dependencies in a 3D tissue space. This kernel function computes similarity between spots in 3D space using learnable scales per dimensionality. The kernel is defined as follows.

Given the coordinates of two sets of points, $a \in \mathbb{R}^{N_x \times 3}$ and $b \in \mathbb{R}^{N_y \times 3}$, where N_x and N_y are the number of spots in each set, we compute the squared Euclidean distance between them:

$$D_{ij} = |\mathbf{a}_i - \mathbf{b}_j|^2 = \mathbf{a}_i^T \mathbf{a}_i + \mathbf{b}_j^T \mathbf{b}_j - 2\mathbf{a}_i^T \mathbf{b}_j. \tag{1}$$

To ensure numerical stability, the distance is clamped as:

$$D_{ij} = \max(D_{ij}, \epsilon_1), \quad \epsilon_1 = 10^{-10}. \tag{2}$$

The scales $\mathbf{s} \in s_1^p, s_2^p, s_3^p$ for perturbagen p per dimensionality in 3D kernel are learnable. Different from those in 2D kernel, to ensure stability with 3D kernel, $\mathbf{s}$ are also clamped:

$$\mathbf{s} = \max(\mathbf{s}, \epsilon_2), \quad \epsilon_2 = 10^{-6}. \tag{3}$$

For self-similarity, when comparing each point with itself (diagonal elements), the squared Euclidean distance simplifies to:

$$D_{ii} = \sum_{d=1}^{3} (a_{id} - b_{id})^2. \tag{4}$$

This formulation ensures smooth and numerically stable computation of the Cauchy kernel in three-dimensional space. Since the numerical instability arises due to sparsity in the third dimension, when approximating posterior parameters of GP latent spaces, we employ the Cholesky decomposition instead of direct matrix inversion to compute the inverse:

$$\mathbf{L} = \text{Cholesky}(\mathbf{A} + \epsilon_3 \mathbf{I}) \quad \text{where } \epsilon_3 = 10^{-6} \tag{5}$$

where A is an intermediate matrix of learning approach (such as K_{pp}) that needs to calculate inversion. The ϵI indicates the added diagonal jitter. Then, the inverse of A is computed in a more stable way using:

$$\mathbf{A}^{-1} = \mathbf{L}^{-T} \mathbf{L}^{-1} \tag{6}$$

The Cholesky decomposition ensures that the intermediate matrices remain symmetric and positive definite, reducing the risk of numerical errors. To achieve these, for the matrix A, we enforce symmetry correction by:

$$\mathbf{A} = (\mathbf{A} + \mathbf{A}^T)/2 \tag{7}$$

And we enforce positive definiteness by computing the eigendecomposition of A:

$$\mathbf{A} = \mathbf{Q} \mathbf{\Lambda} \mathbf{Q}^T \tag{8}$$

where $\mathbf{Q}$ is the matrix of eigenvectors, $\mathbf{\Lambda} = \text{diag}(\lambda_1, \lambda_2, \ldots, \lambda_n)$ is the diagonal matrix of eigenvalues. If the smallest eigenvalue satisfies:

$$\min(\lambda_i) < \epsilon_4, \quad \text{where } \epsilon_4 = 10^{-6} \tag{9}$$

we clamp the eigenvalues to ensure positive definiteness:

$$\lambda_i' = \max(\lambda_i, \epsilon_4) \tag{10}$$

With the modified diagonal matrix $\mathbf{\Lambda}' = \text{diag}(\lambda_1', \lambda_2', \ldots, \lambda_n')$, the corrected matrix is then reconstructed as:

$$\mathbf{A} = \mathbf{Q}\mathbf{\Lambda}'\mathbf{Q}^T \tag{11}$$

This method guarantees that $\mathbf{A}$ remains positive definite, preventing numerical issues in Cholesky decomposition. Notably, to further enhance stability and prevent singularity issues, we introduce larger general jitter terms in the 3D model ($\epsilon_2, \epsilon_3, \epsilon_4$), which mitigate the effects of ill-conditioning and avoid computationally undefined operations.

Supplementary Tables

All supplementary tables (1-16) are provided in a separate file:

`CONCERT_Supplementary_tables.xlsx`

Supplementary Table 1. P-values of the comparisons between CONCERT and all competing methods in benchmarking experiments of patch perturbation task on Perturb-map datasets evaluated by E-distance.

Supplementary Table 2. P-values of the comparisons between CONCERT and all competing methods in benchmarking experiments of patch perturbation task on Perturb-map datasets evaluated by mean absolute error.

Supplementary Table 3. P-values of the comparisons between CONCERT and all competing methods in benchmarking experiments of patch perturbation task on Perturb-map datasets evaluated by Pearson correlation coefficient.

Supplementary Table 4. P-values of the comparisons between CONCERT and all competing methods in benchmarking experiments of patch perturbation task on Perturb-map datasets evaluated by R^2.

Supplementary Table 5. P-values of the comparisons between CONCERT and all competing methods in benchmarking experiments of border perturbation task on Perturb-map datasets evaluated by E-distance.

Supplementary Table 6. P-values of the comparisons between CONCERT and all competing methods in benchmarking experiments of border perturbation task on Perturb-map datasets evaluated by mean absolute error.

Supplementary Table 7. P-values of the comparisons between CONCERT and all competing methods in benchmarking experiments of border perturbation task on Perturb-map datasets evaluated by Pearson correlation coefficient.

Supplementary Table 8. P-values of the comparisons between CONCERT and all competing methods in benchmarking experiments of border perturbation task on Perturb-map datasets evaluated by R^2.

Supplementary Table 9. P-values of the comparisons between CONCERT and all competing methods in benchmarking experiments of niche perturbation task on Perturb-map datasets evaluated by E-distance.

Supplementary Table 10. P-values of the comparisons between CONCERT and all competing methods in benchmarking experiments of niche perturbation task on Perturb-map datasets evaluated by mean absolute error.

Supplementary Table 11. P-values of the comparisons between CONCERT and all competing methods in benchmarking experiments of niche perturbation task on Perturb-map datasets evaluated by Pearson correlation coefficient.

Supplementary Table 12. P-values of the comparisons between CONCERT and all competing methods in benchmarking experiments of niche perturbation task on Perturb-map datasets evaluated by R^2.

Supplementary Table 13. P-values of the comparisons between CONCERT and all competing methods in benchmarking experiments of cross-niche patch perturbation task on Perturb-map datasets evaluated by E-distance.

Supplementary Table 14. P-values of the comparisons between CONCERT and all competing methods in benchmarking experiments of cross-niche patch perturbation task on Perturb-map datasets evaluated by mean absolute error.

Supplementary Table 15. P-values of the comparisons between CONCERT and all competing meth-

ods in benchmarking experiments of cross-niche patch perturbation task on Perturb-map datasets evaluated by Pearson correlation coefficient.

Supplementary Table 16. P-values of the comparisons between CONCERT and all competing methods in benchmarking experiments of cross-niche patch perturbation task on Perturb-map datasets evaluated by R^2.

Supplementary Figures

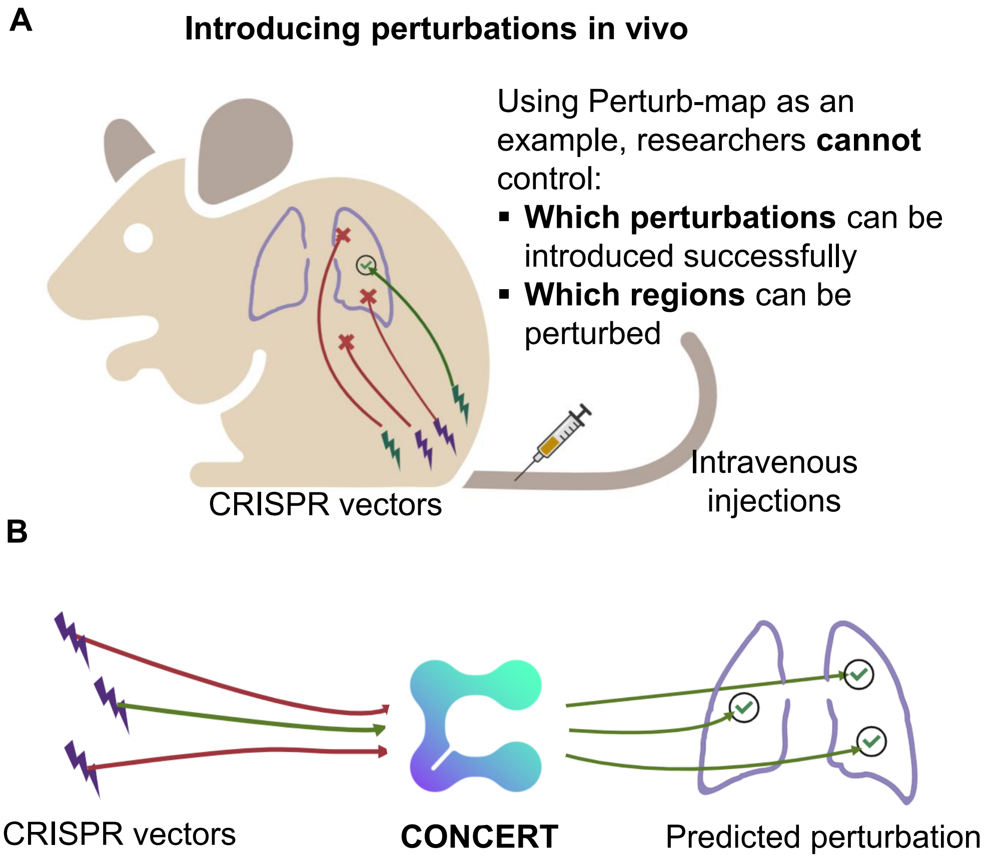

Supplementary Fig. 1: Problems in spatial perturbation technologies. Potential issues in current spatial perturbation sequencing technologies (a) and the motivation to develop CONCERT (b).

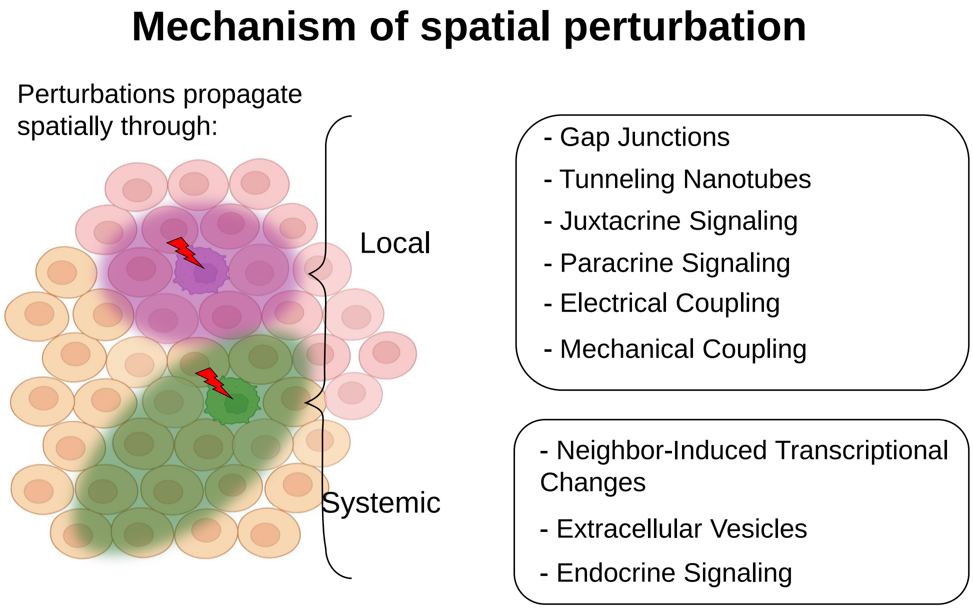

Supplementary Fig. 2: Mechanisms of the dispersion of perturbation effects in tissue.

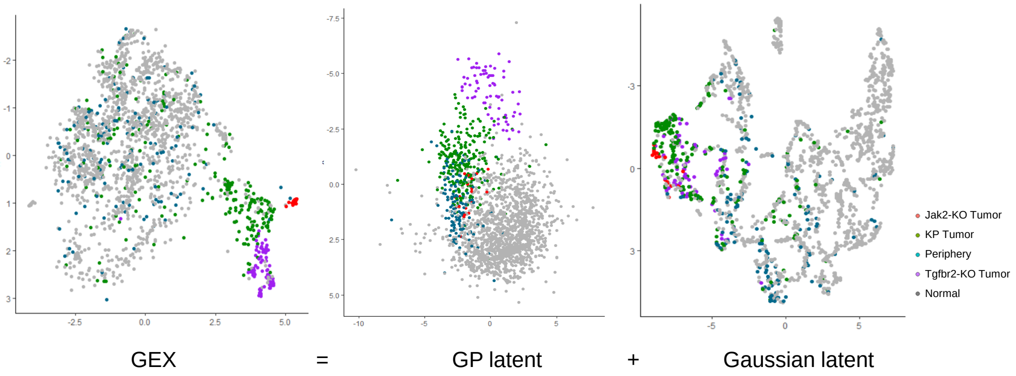

Supplementary Fig. 3: Disentanglement of the latent space of CONCERT. CONCERT can learn a Gaussian and a GP latent space, capturing the perturbation effects on each cell and on cells' interactions, respectively. U-map is used for visualization if the dimension of the embedding is larger than 2. GEX indicates gene expression.

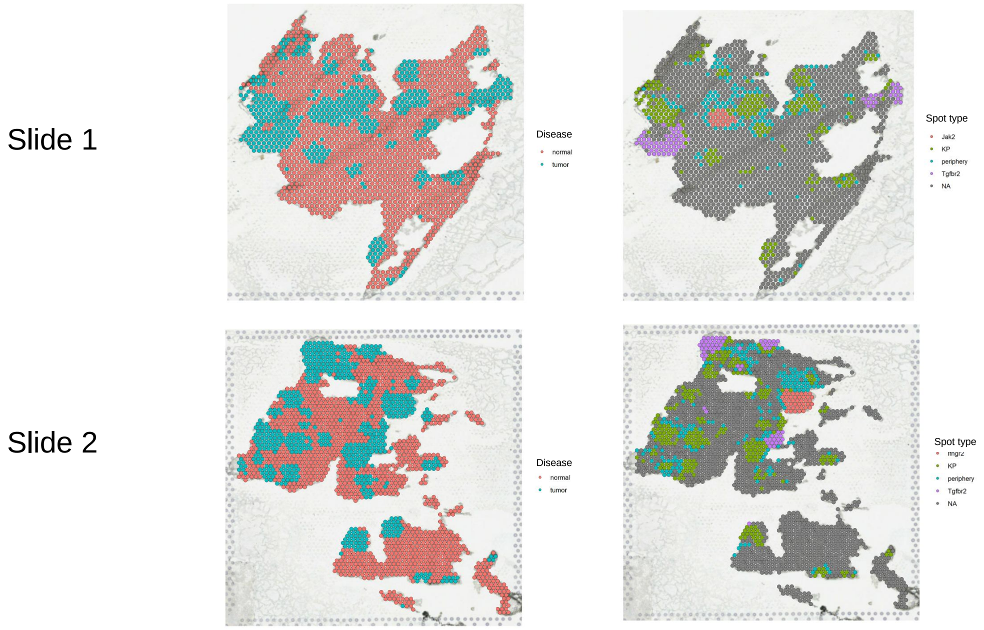

Supplementary Fig. 4: Annotated slide GSM5808054 and GSM5808055. Labels of disease and spot state provided in the original paper.

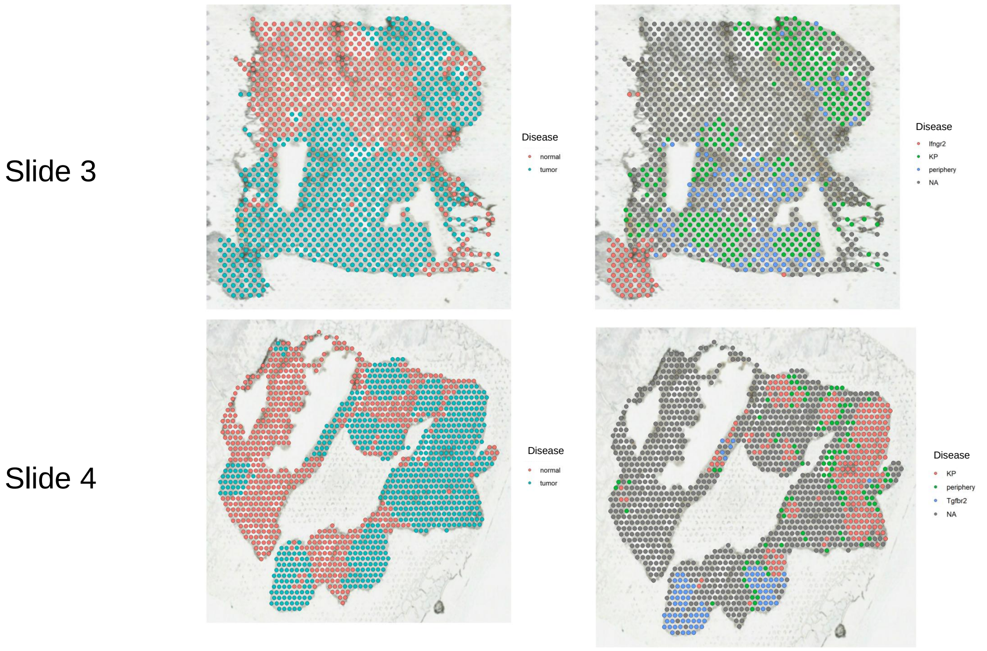

Supplementary Fig. 5: Annotated slide GSM5808056 and GSM5808057. Labels of disease and spot state provided in the original paper.

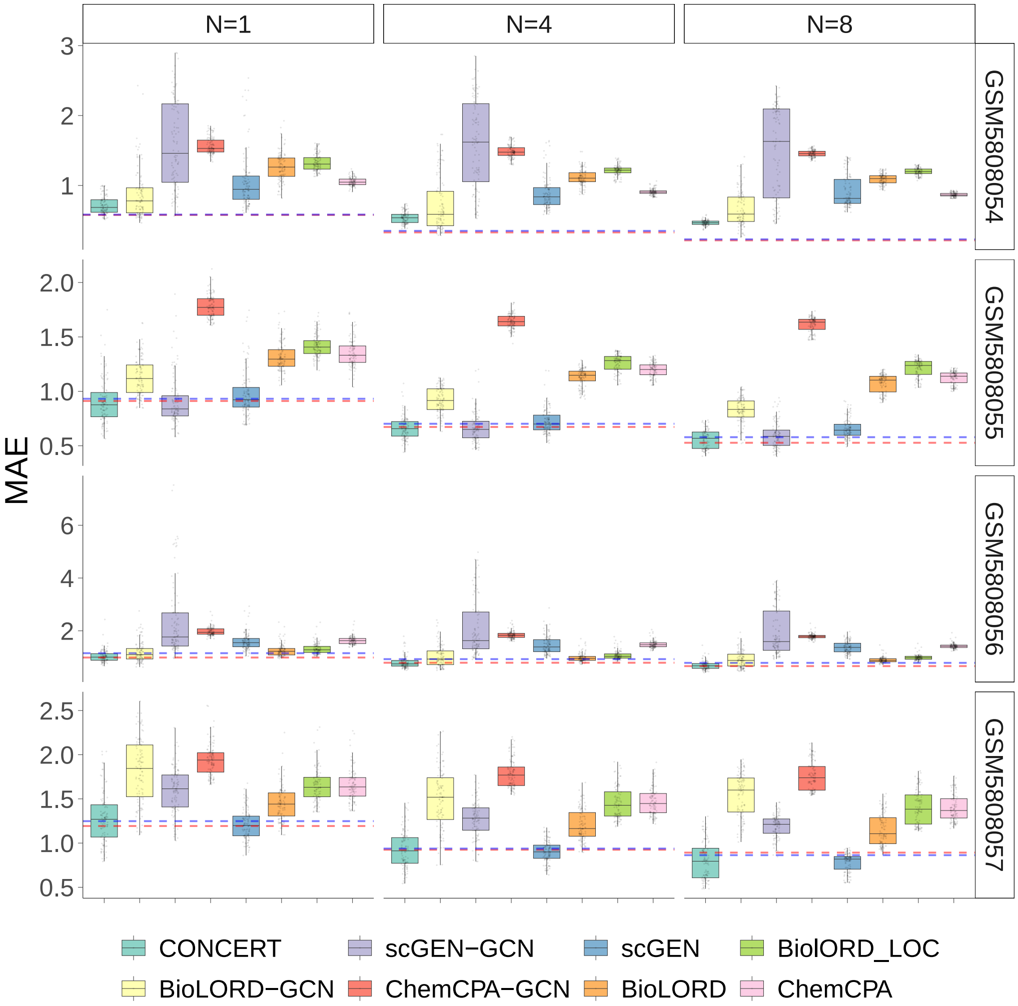

Supplementary Fig. 6: Benchmarking experiments of patch perturbation task on Perturb-map datasets evaluated by mean absolute error. KNN-SP and KNN-GEX are shown by red and blue dotted line, respectively. Lower value indicates better performance. The central line inside the box represents the median, while the top and bottom edges correspond to the first (Q1) and third (Q3) quartiles. The whiskers extend to the smallest and largest values within 1.5 times the interquartile range (IQR) from the quartiles. P-values from the comparisons between CONCERT and competing methods are provided in supplementary table 2.

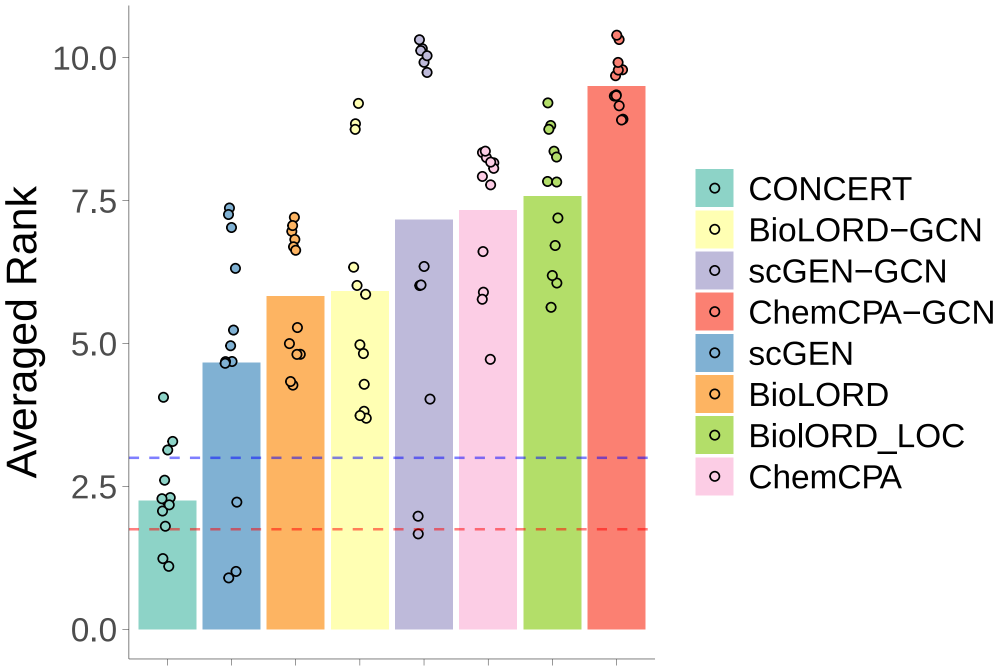

Supplementary Fig. 7: Average ranks of the patch perturbation task on Perturb-map datasets evaluated by mean absolute error. KNN-SP and KNN-GEX are shown by red and blue dotted line, respectively. Lower value indicates better performance.

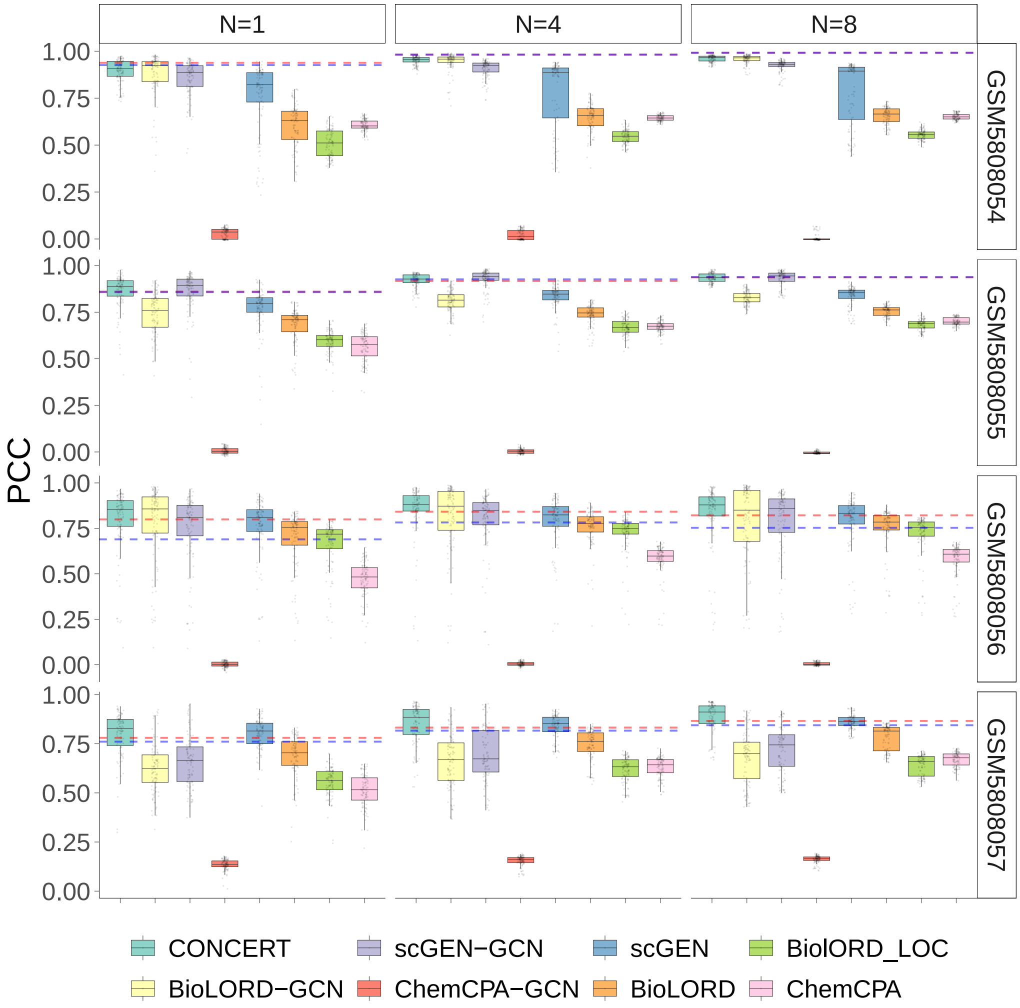

Supplementary Fig. 8: Benchmarking experiments of patch perturbation task on Perturb-map datasets evaluated by Pearson correlation coefficient. KNN-SP and KNN-GEX are shown by red and blue dotted line, respectively. Higher value indicates better performance. The central line inside the box represents the median, while the top and bottom edges correspond to the first (Q1) and third (Q3) quartiles. The whiskers extend to the smallest and largest values within 1.5 times the interquartile range (IQR) from the quartiles. P-values from the comparisons between CONCERT and competing methods are provided in supplementary table 3.

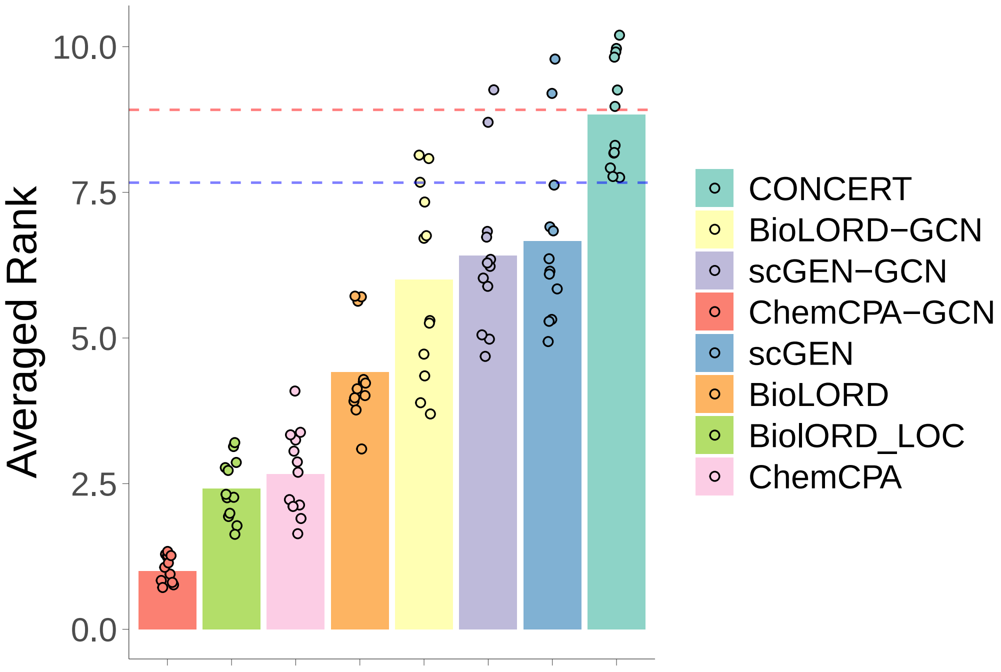

Supplementary Fig. 9: Average ranks of the patch perturbation task on Perturb-map datasets evaluated by Pearson correlation coefficient. KNN-SP and KNN-GEX are shown by red and blue dotted line, respectively. Lower value indicates better performance.

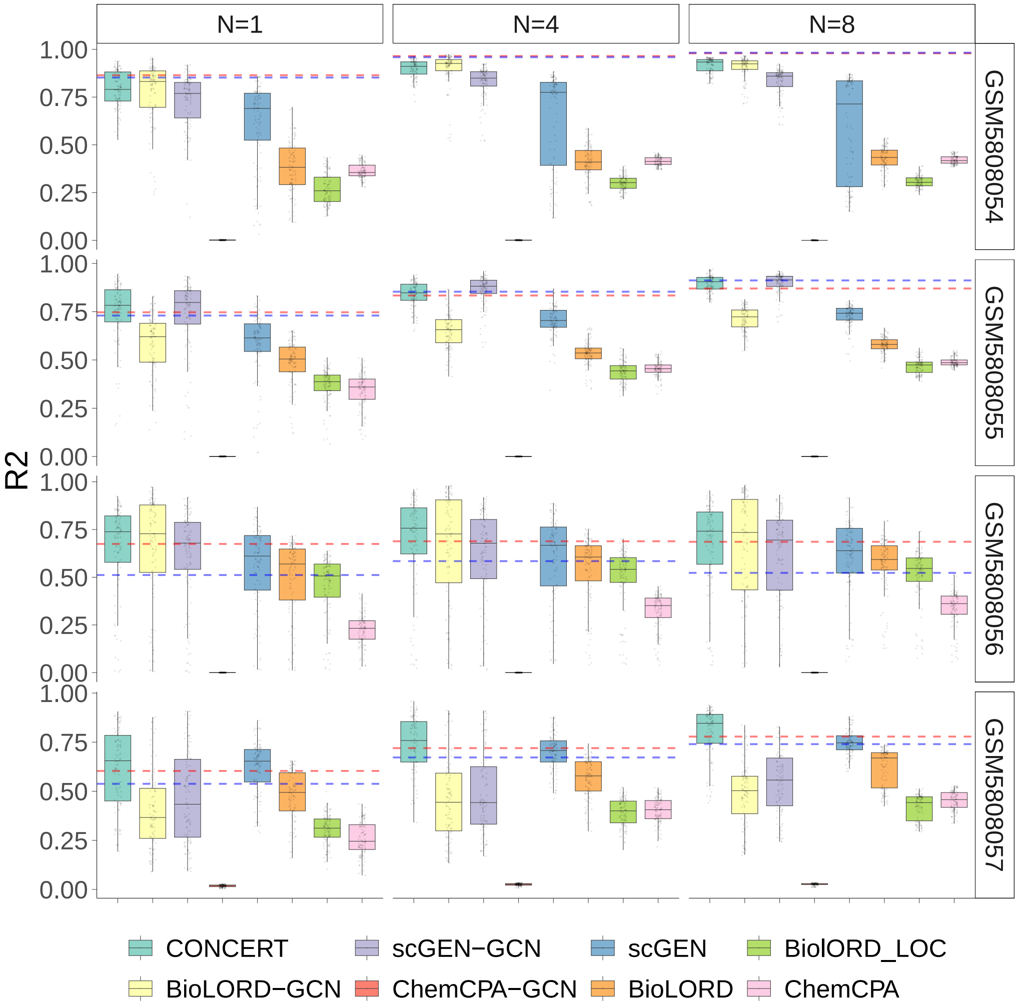

Supplementary Fig. 10: Benchmarking experiments of patch perturbation task on Perturb-map datasets evaluated by R^2. KNN-SP and KNN-GEX are shown by red and blue dotted line, respectively. Higher value indicates better performance. The central line inside the box represents the median, while the top and bottom edges correspond to the first (Q1) and third (Q3) quartiles. The whiskers extend to the smallest and largest values within 1.5 times the interquartile range (IQR) from the quartiles. P-values from the comparisons between CONCERT and competing methods are provided in supplementary table 4.

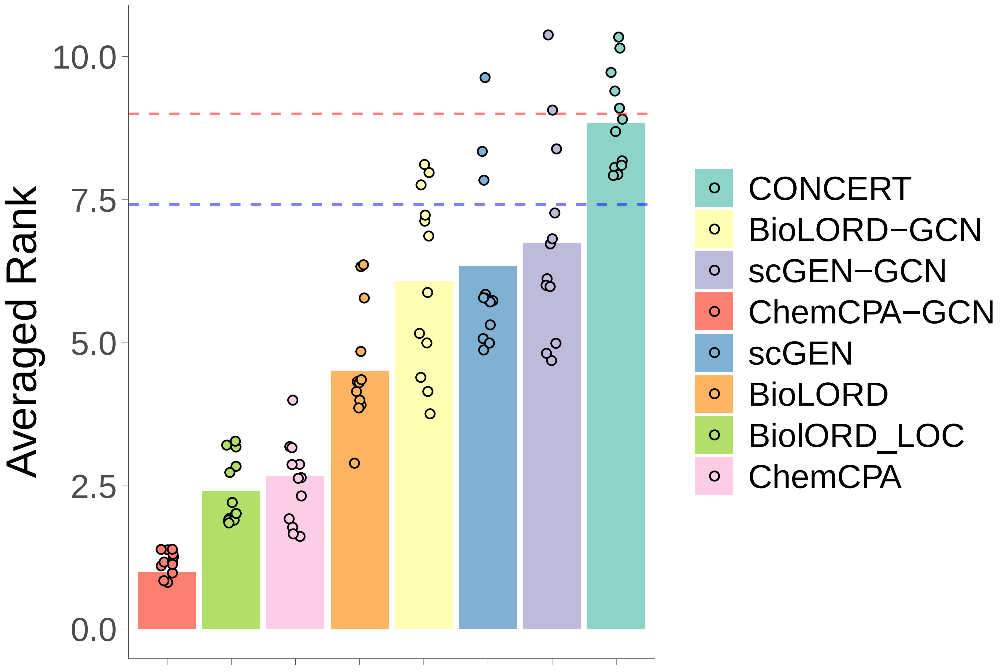

Supplementary Fig. 11: Average ranks of the patch perturbation task on Perturb-map datasets evaluated by R^2. KNN-SP and KNN-GEX are shown by red and blue dotted line, respectively.

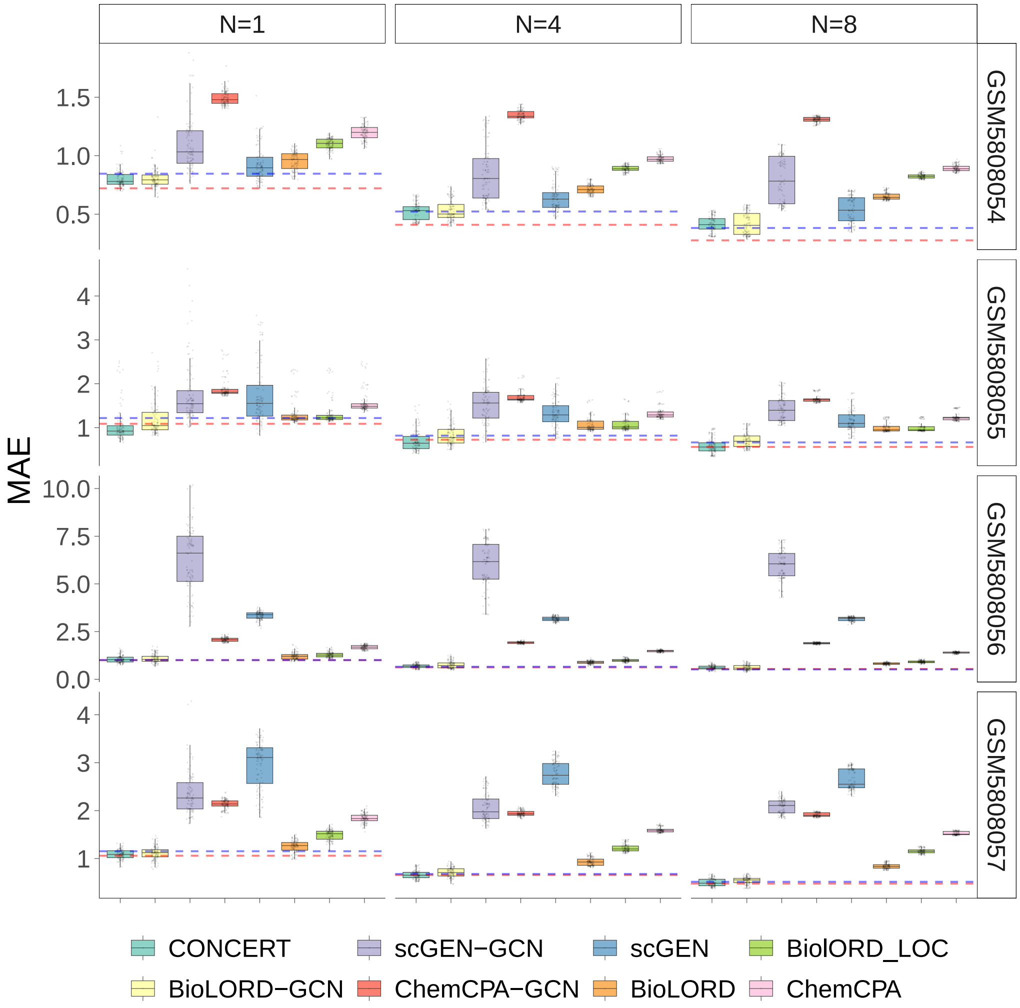

Supplementary Fig. 12: Benchmarking experiments of border perturbation task on Perturb-map datasets evaluated by mean absolute error. KNN-SP and KNN-GEX are shown by red and blue dotted line, respectively. Lower value indicates better performance. The central line inside the box represents the median, while the top and bottom edges correspond to the first (Q1) and third (Q3) quartiles. The whiskers extend to the smallest and largest values within 1.5 times the interquartile range (IQR) from the quartiles. P-values from the comparisons between CONCERT and competing methods are provided in supplementary table 6.

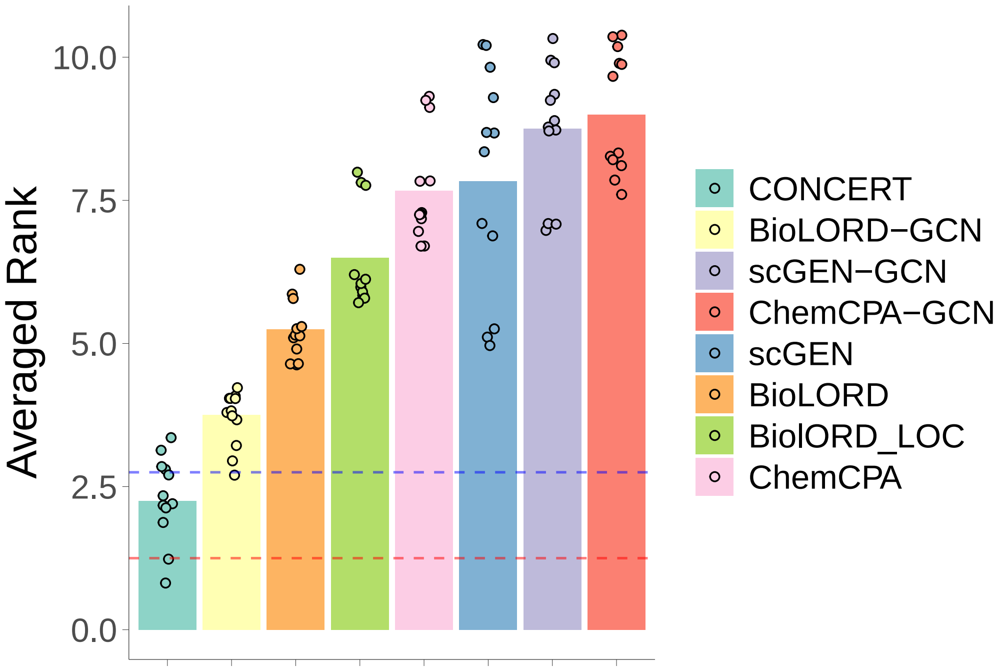

Supplementary Fig. 13: Average ranks of the border perturbation task on Perturb-map datasets evaluated by mean absolute error. KNN-SP and KNN-GEX are shown by red and blue dotted line, respectively. Lower value indicates better performance.

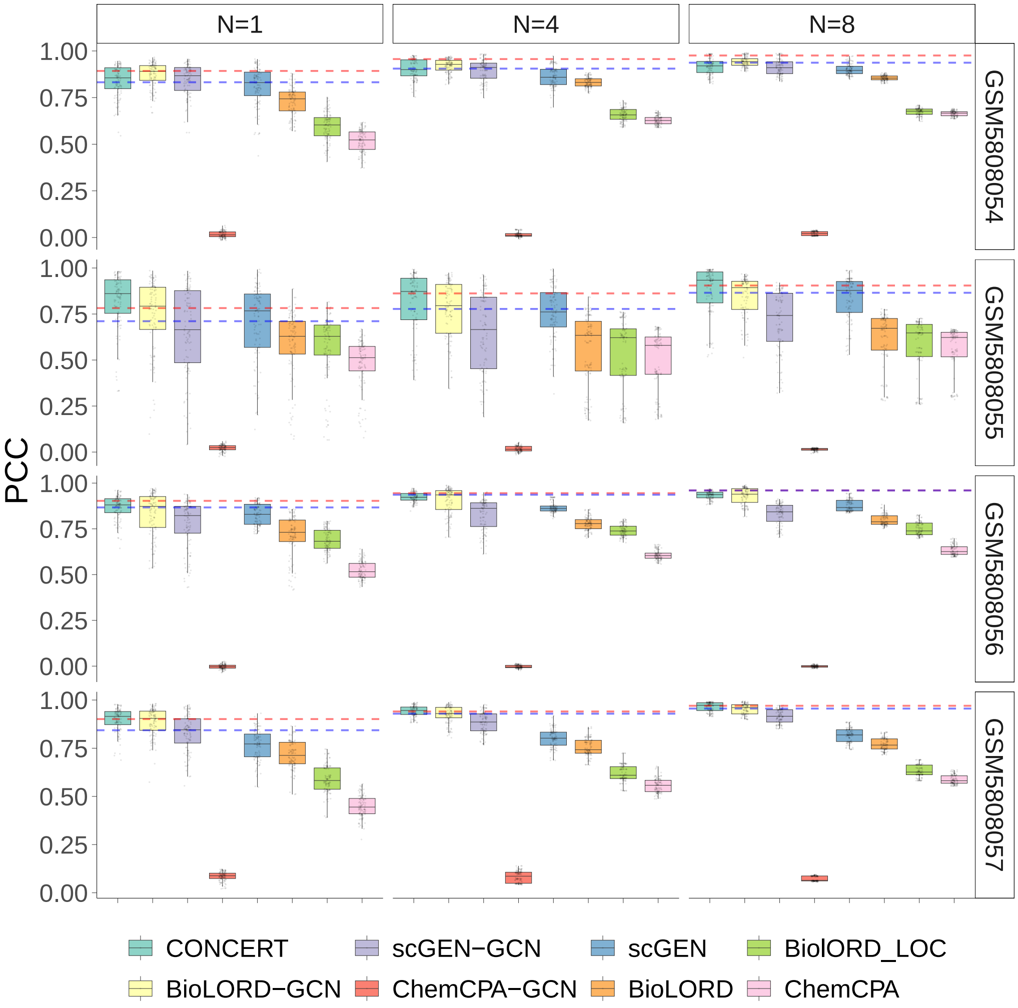

Supplementary Fig. 14: Benchmarking experiments of border perturbation task on Perturb-map datasets evaluated by Pearson correlation coefficient. KNN-SP and KNN-GEX are shown by red and blue dotted line, respectively. Higher value indicates better performance. The central line inside the box represents the median, while the top and bottom edges correspond to the first (Q1) and third (Q3) quartiles. The whiskers extend to the smallest and largest values within 1.5 times the interquartile range (IQR) from the quartiles. P-values from the comparisons between CONCERT and competing methods are provided in supplementary table 7.

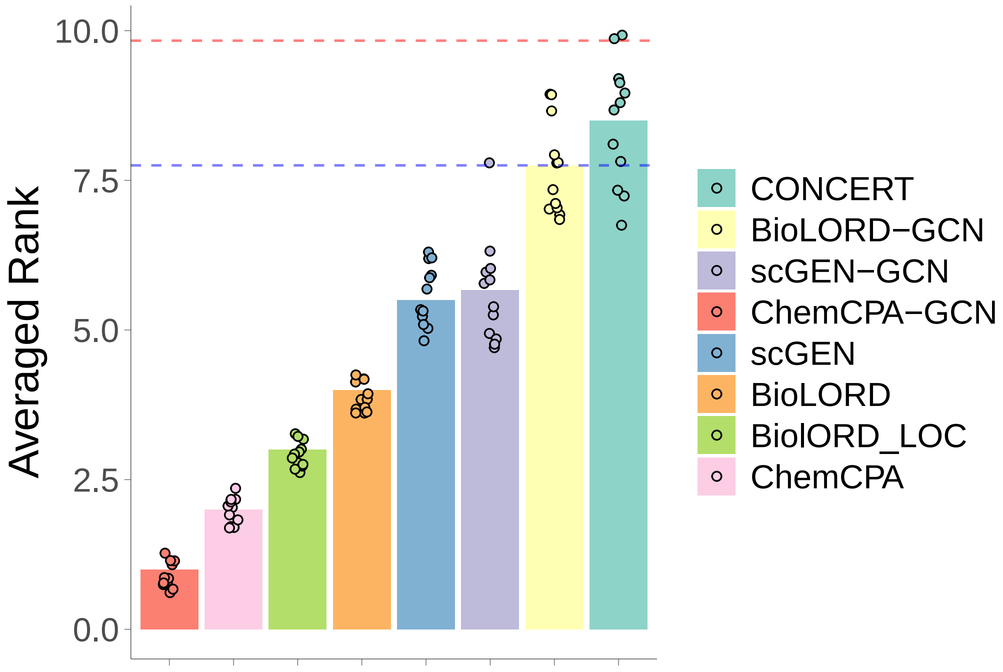

Supplementary Fig. 15: Average ranks of the border perturbation task on Perturb-map datasets evaluated by Pearson correlation coefficient. KNN-SP and KNN-GEX are shown by red and blue dotted line, respectively. Lower value indicates better performance.

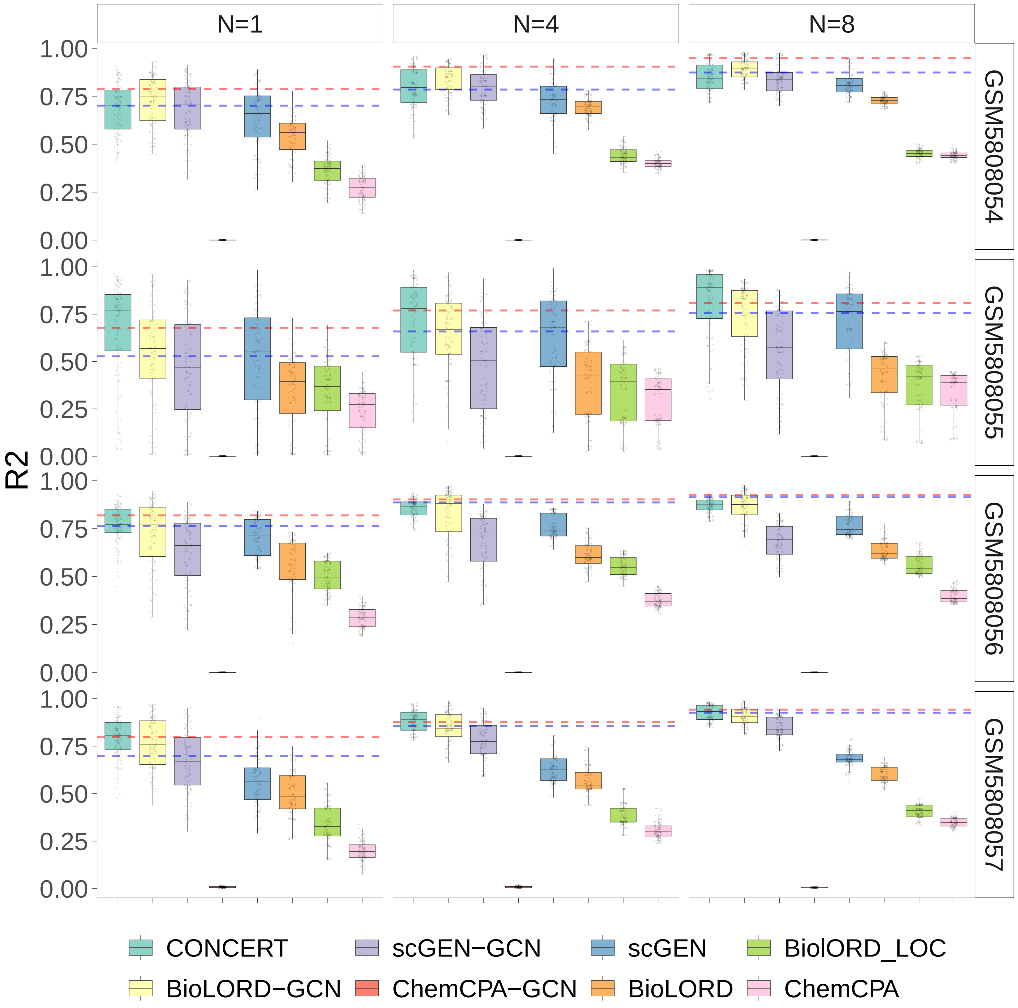

Supplementary Fig. 16: Benchmarking experiments of border perturbation task on Perturb-map datasets evaluated by R^2. KNN-SP and KNN-GEX are shown by red and blue dotted line, respectively. Higher value indicates better performance. The central line inside the box represents the median, while the top and bottom edges correspond to the first (Q1) and third (Q3) quartiles. The whiskers extend to the smallest and largest values within 1.5 times the interquartile range (IQR) from the quartiles. P-values from the comparisons between CONCERT and competing methods are provided in supplementary table 8.

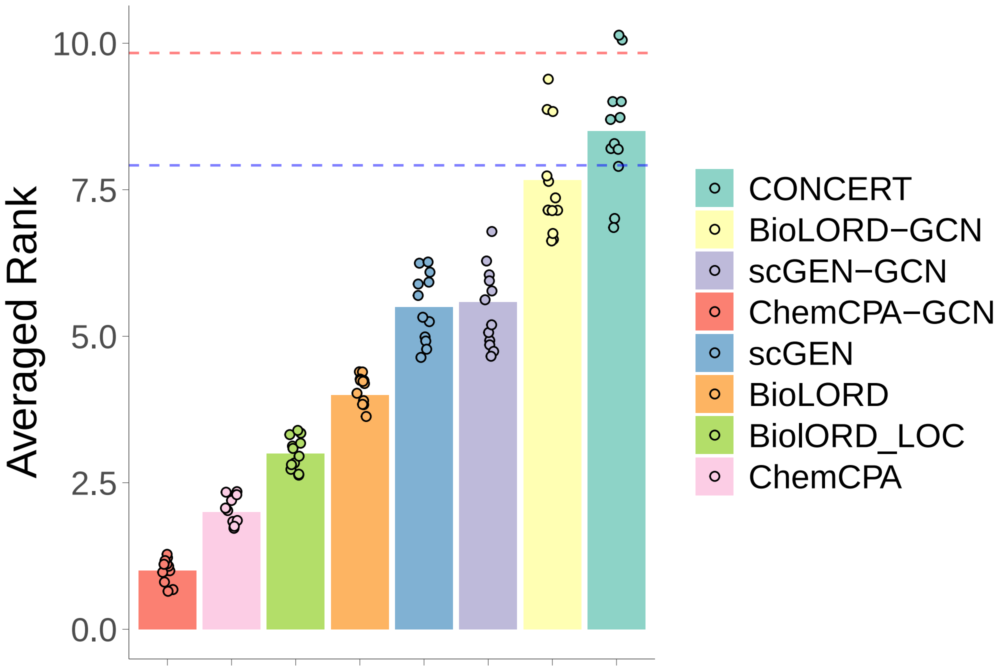

Supplementary Fig. 17: Average ranks of the border perturbation task on Perturb-map datasets evaluated by R^2. KNN-SP and KNN-GEX are shown by red and blue dotted line, respectively. Lower value indicates better performance.

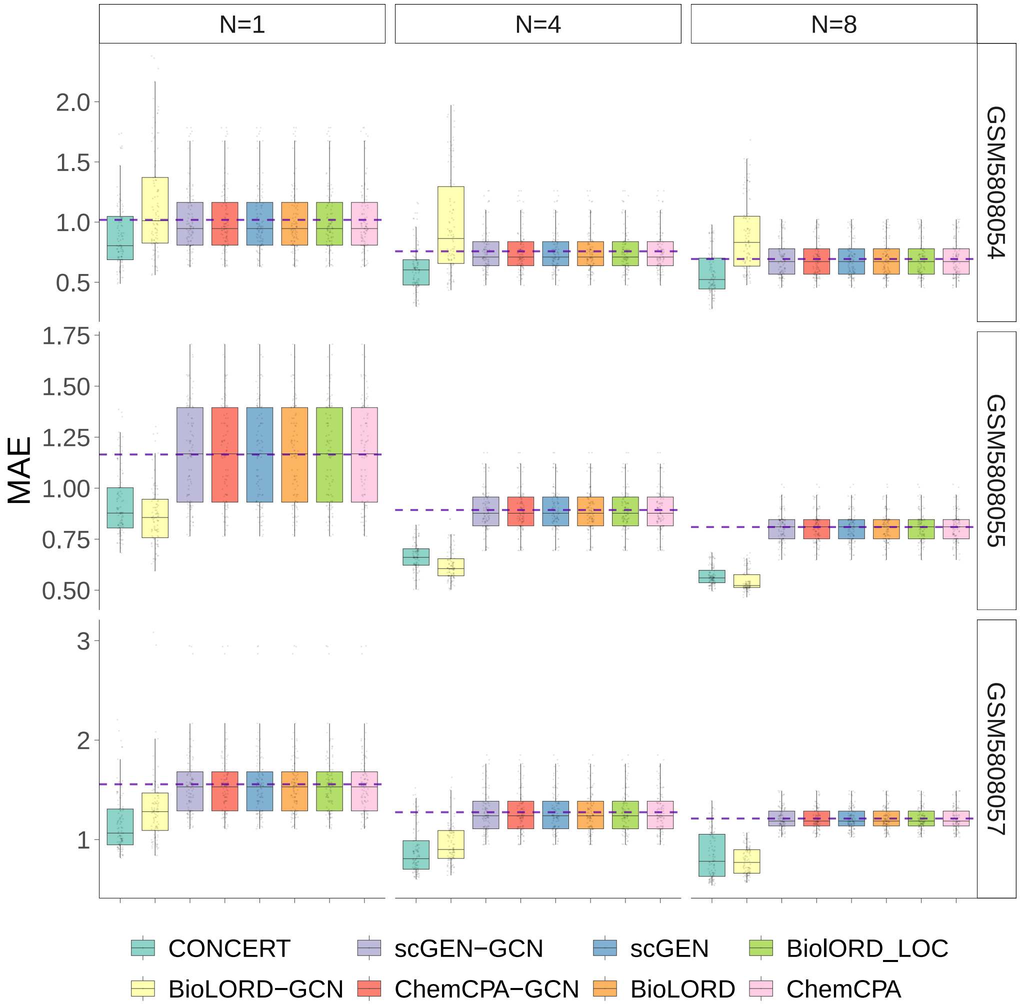

Supplementary Fig. 18: Benchmarking experiments of niche perturbation task on Perturb-map datasets evaluated by mean absolute error. KNN-SP and KNN-GEX are shown by red and blue dotted line, respectively. Lower value indicates better performance. The central line inside the box represents the median, while the top and bottom edges correspond to the first (Q1) and third (Q3) quartiles. The whiskers extend to the smallest and largest values within 1.5 times the interquartile range (IQR) from the quartiles. P-values from the comparisons between CONCERT and competing methods are provided in supplementary table 10.

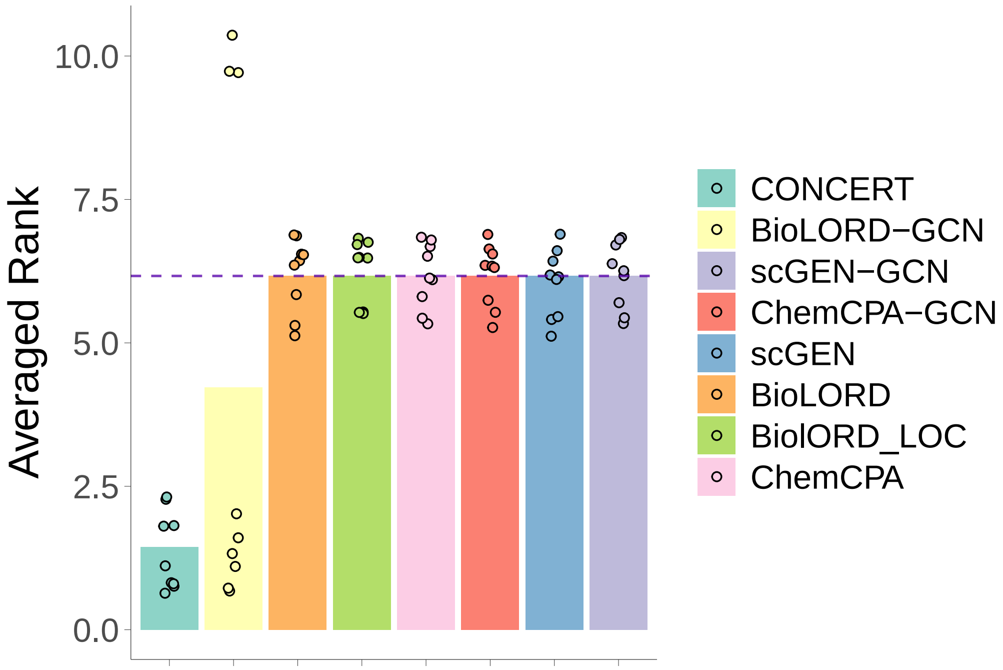

Supplementary Fig. 19: Average ranks of the niche perturbation task on Perturb-map datasets evaluated by mean absolute error. KNN-SP and KNN-GEX are shown by red and blue dotted line, respectively. Lower value indicates better performance.

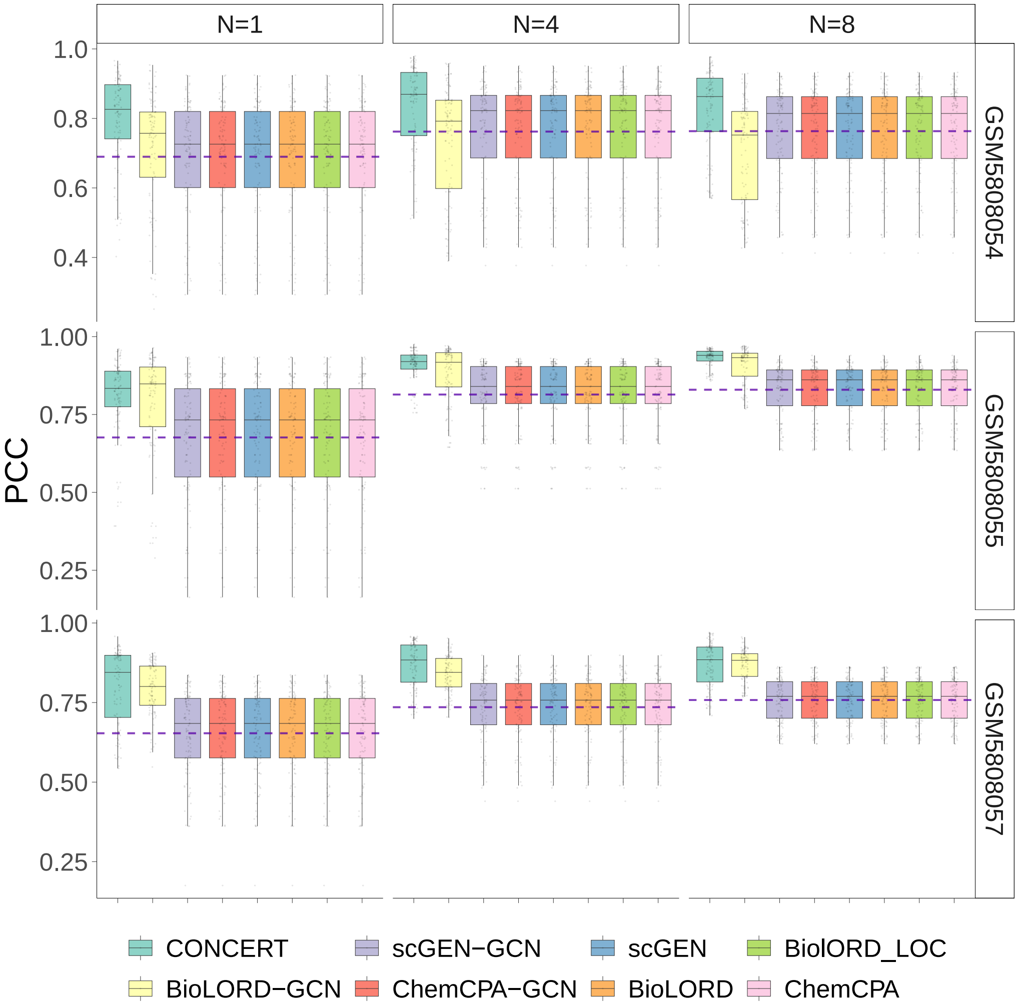

Supplementary Fig. 20: Benchmarking experiments of niche perturbation task on Perturb-map datasets evaluated by Pearson correlation coefficient. KNN-SP and KNN-GEX are shown by red and blue dotted line, respectively. Higher value indicates better performance. The central line inside the box represents the median, while the top and bottom edges correspond to the first (Q1) and third (Q3) quartiles. The whiskers extend to the smallest and largest values within 1.5 times the interquartile range (IQR) from the quartiles. P-values from the comparisons between CONCERT and competing methods are provided in supplementary table 11.

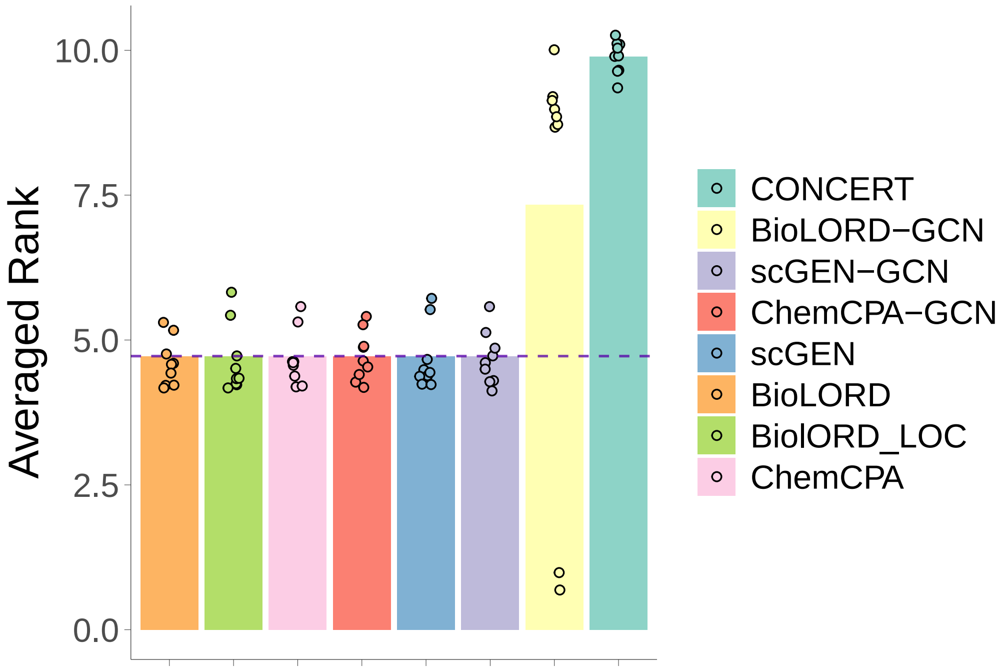

Supplementary Fig. 21: Average ranks of the niche perturbation task on Perturb-map datasets evaluated by Pearson correlation coefficient. KNN-SP and KNN-GEX are shown by red and blue dotted line, respectively. Higher value indicates better performance.

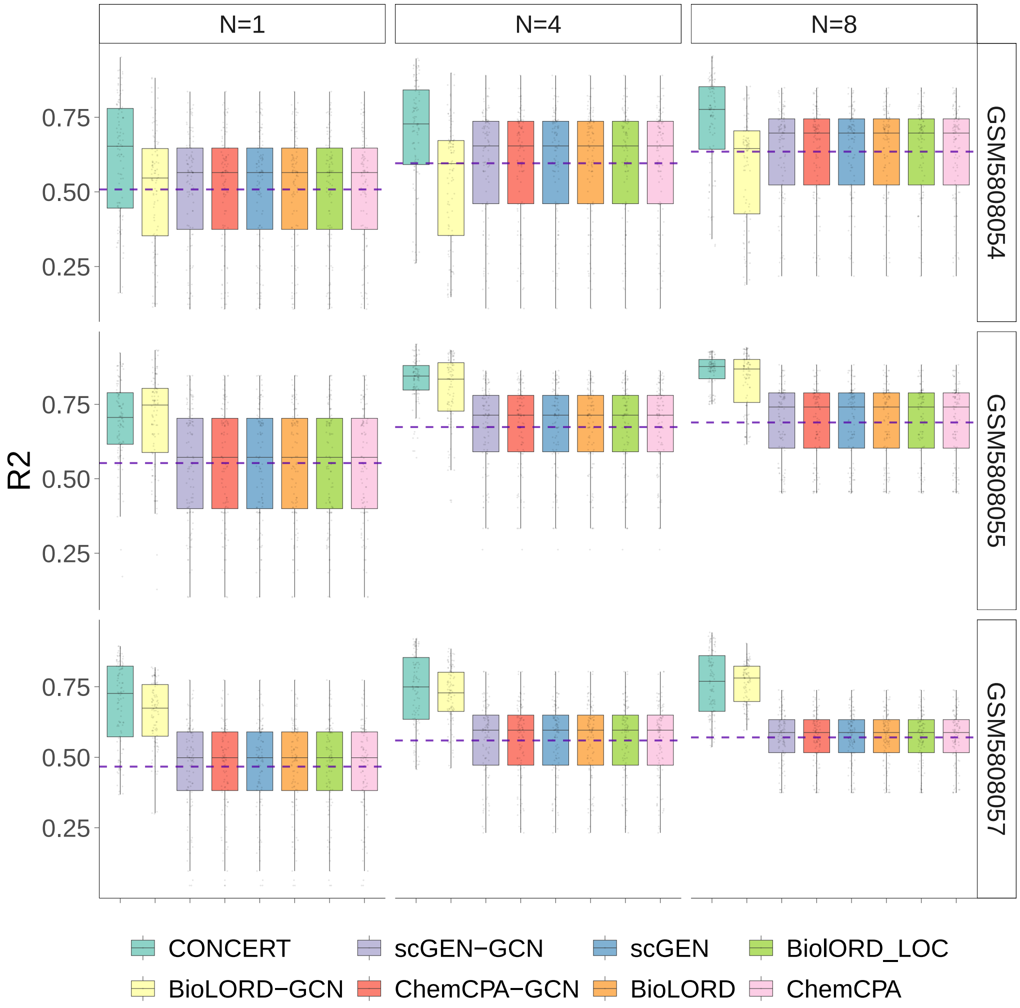

Supplementary Fig. 22: Benchmarking experiments of niche perturbation task on Perturb-map datasets evaluated by R^2. KNN-SP and KNN-GEX are shown by red and blue dotted line, respectively. Higher value indicates better performance. The central line inside the box represents the median, while the top and bottom edges correspond to the first (Q1) and third (Q3) quartiles. The whiskers extend to the smallest and largest values within 1.5 times the interquartile range (IQR) from the quartiles. P-values from the comparisons between CONCERT and competing methods are provided in supplementary table 12.

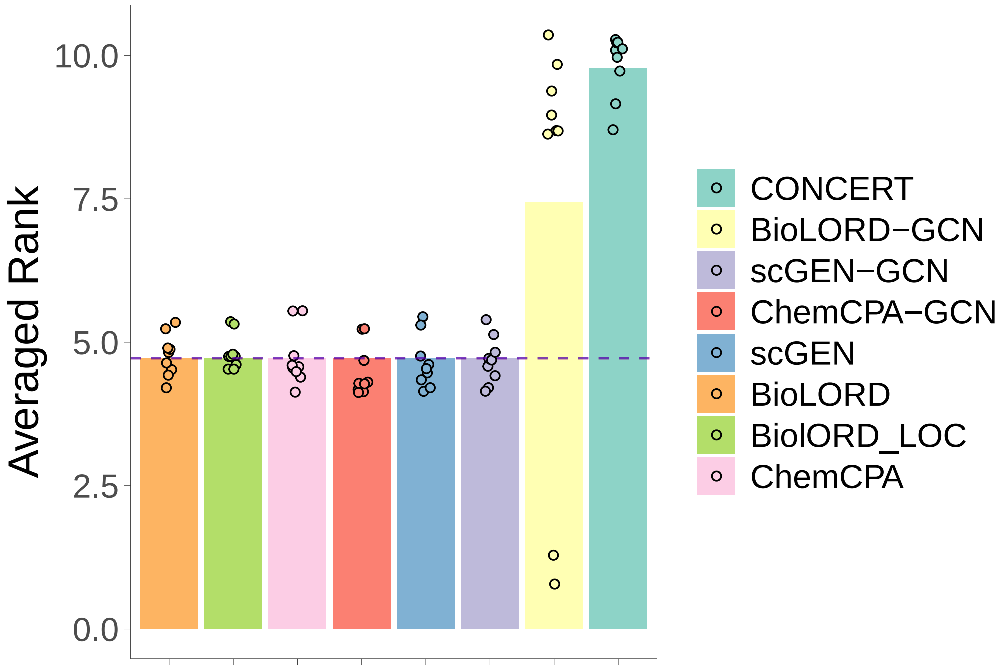

Supplementary Fig. 23: Average ranks of the niche perturbation task on Perturb-map datasets evaluated by R^2. KNN-SP and KNN-GEX are shown by red and blue dotted line, respectively. Higher value indicates better performance.

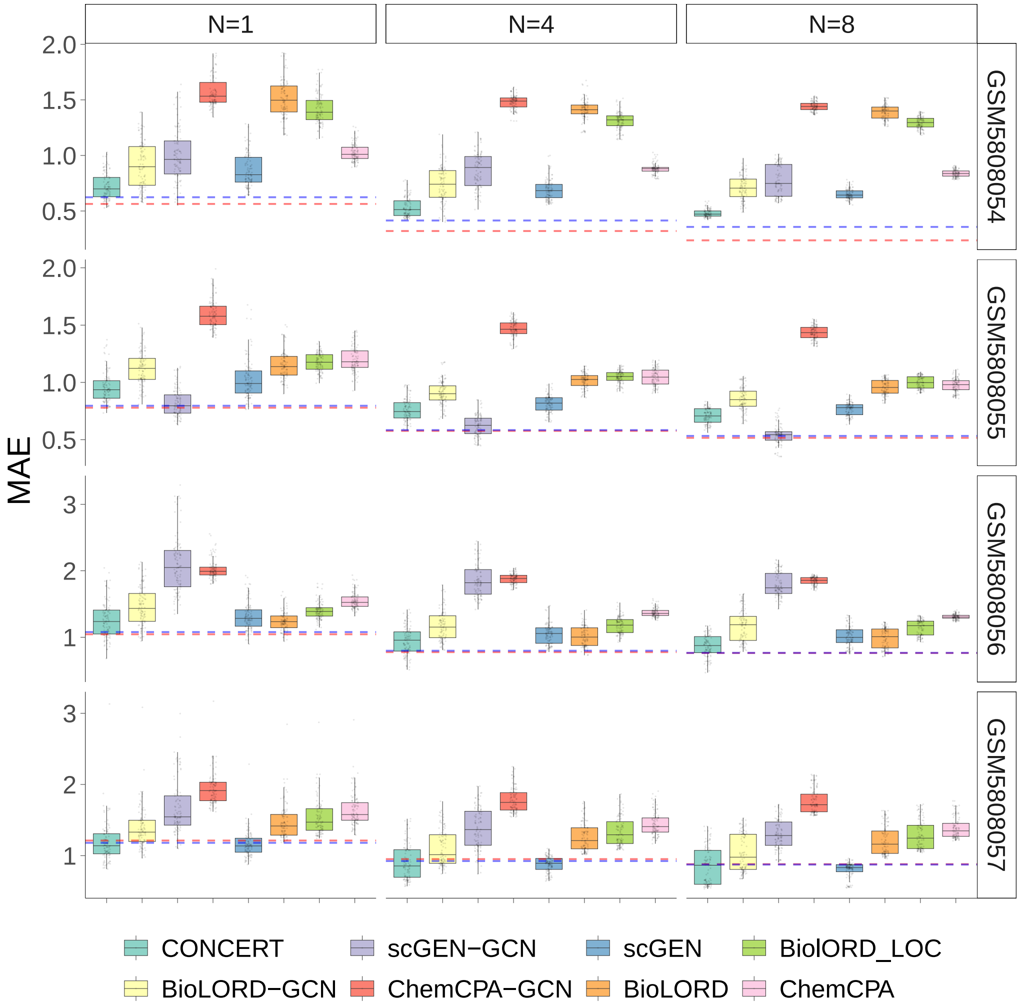

Supplementary Fig. 24: Benchmarking experiments of cross-niche patch perturbation task on Perturb-map datasets evaluated by mean absolute error. KNN-SP and KNN-GEX are shown by red and blue dotted line, respectively. Lower value indicates better performance. The central line inside the box represents the median, while the top and bottom edges correspond to the first (Q1) and third (Q3) quartiles. The whiskers extend to the smallest and largest values within 1.5 times the interquartile range (IQR) from the quartiles. P-values from the comparisons between CONCERT and competing methods are provided in supplementary table 14.

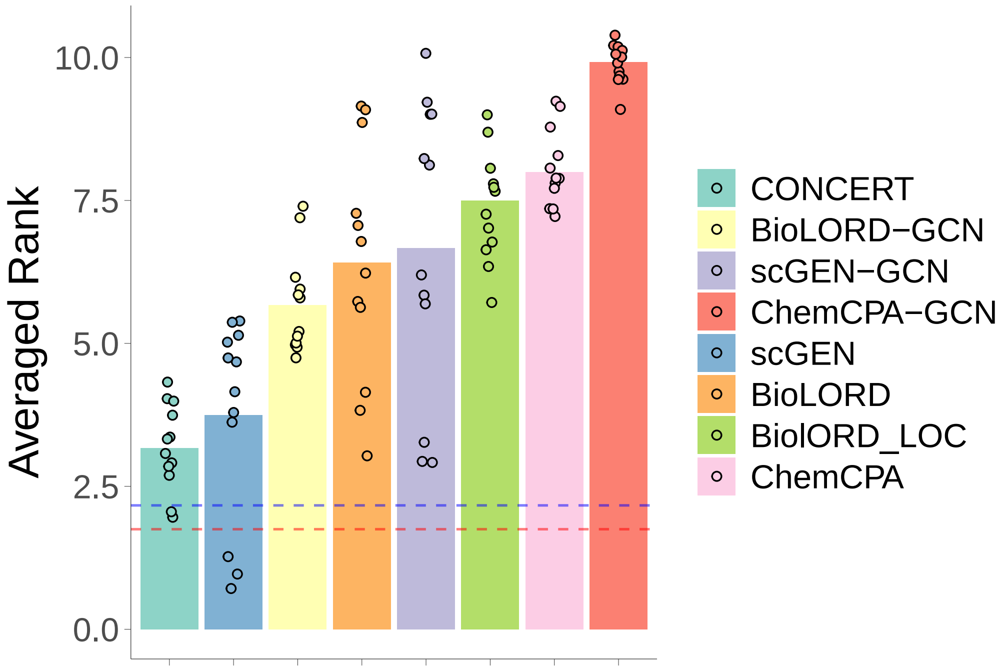

Supplementary Fig. 25: Average ranks of the cross-niche patch perturbation task on Perturb-map datasets evaluated by mean absolute error. KNN-SP and KNN-GEX are shown by red and blue dotted line, respectively. Lower value indicates better performance.

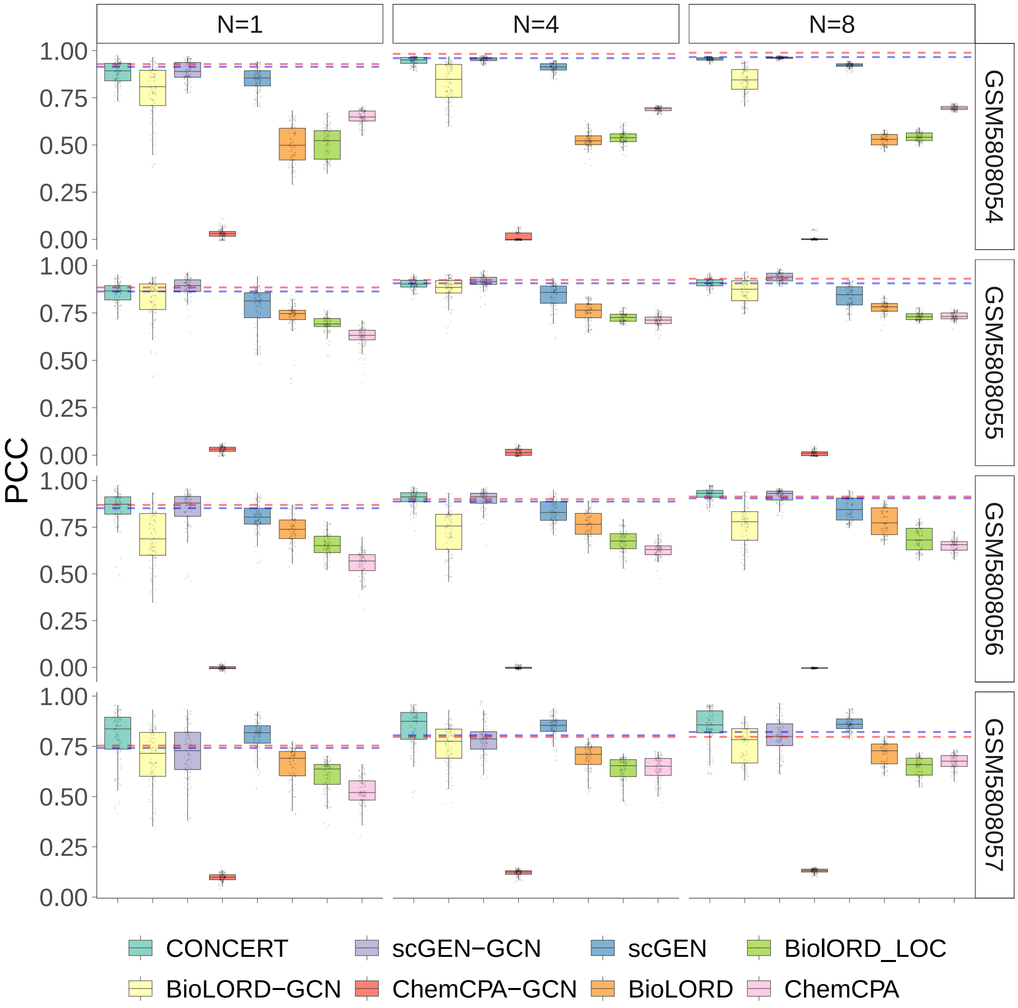

Supplementary Fig. 26: Benchmarking experiments of cross-niche patch perturbation task on Perturb-map datasets evaluated by Pearson correlation coefficient. KNN-SP and KNN-GEX are shown by red and blue dotted line, respectively. Higher value indicates better performance. The central line inside the box represents the median, while the top and bottom edges correspond to the first (Q1) and third (Q3) quartiles. The whiskers extend to the smallest and largest values within 1.5 times the interquartile range (IQR) from the quartiles. P-values from the comparisons between CONCERT and competing methods are provided in supplementary table 15.

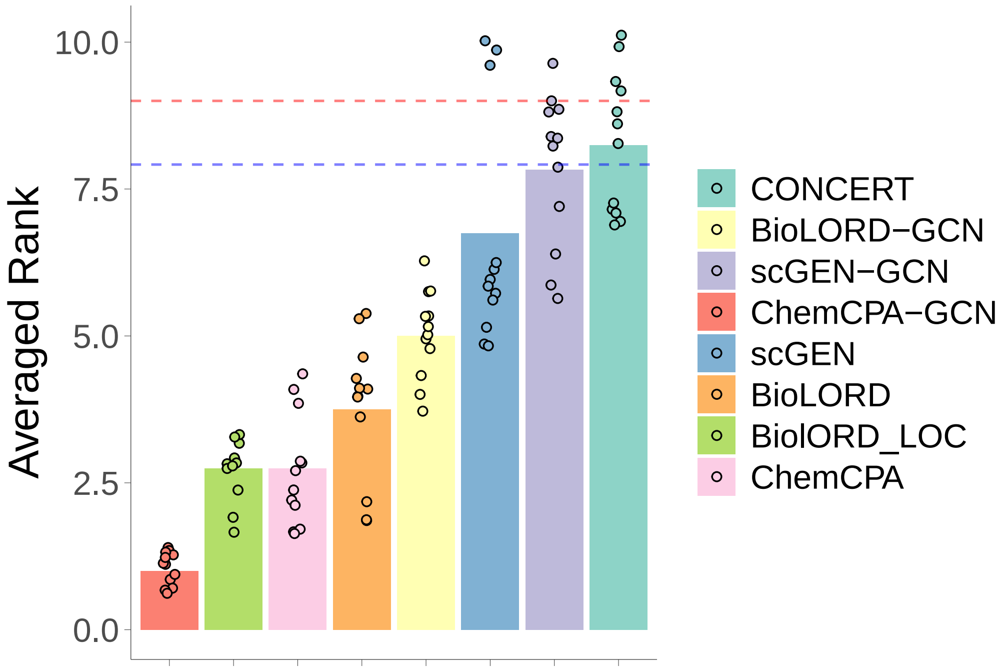

Supplementary Fig. 27: Average ranks of the cross-niche patch perturbation task on Perturb-map datasets evaluated by Pearson correlation coefficient. KNN-SP and KNN-GEX are shown by red and blue dotted line, respectively. Higher value indicates better performance.

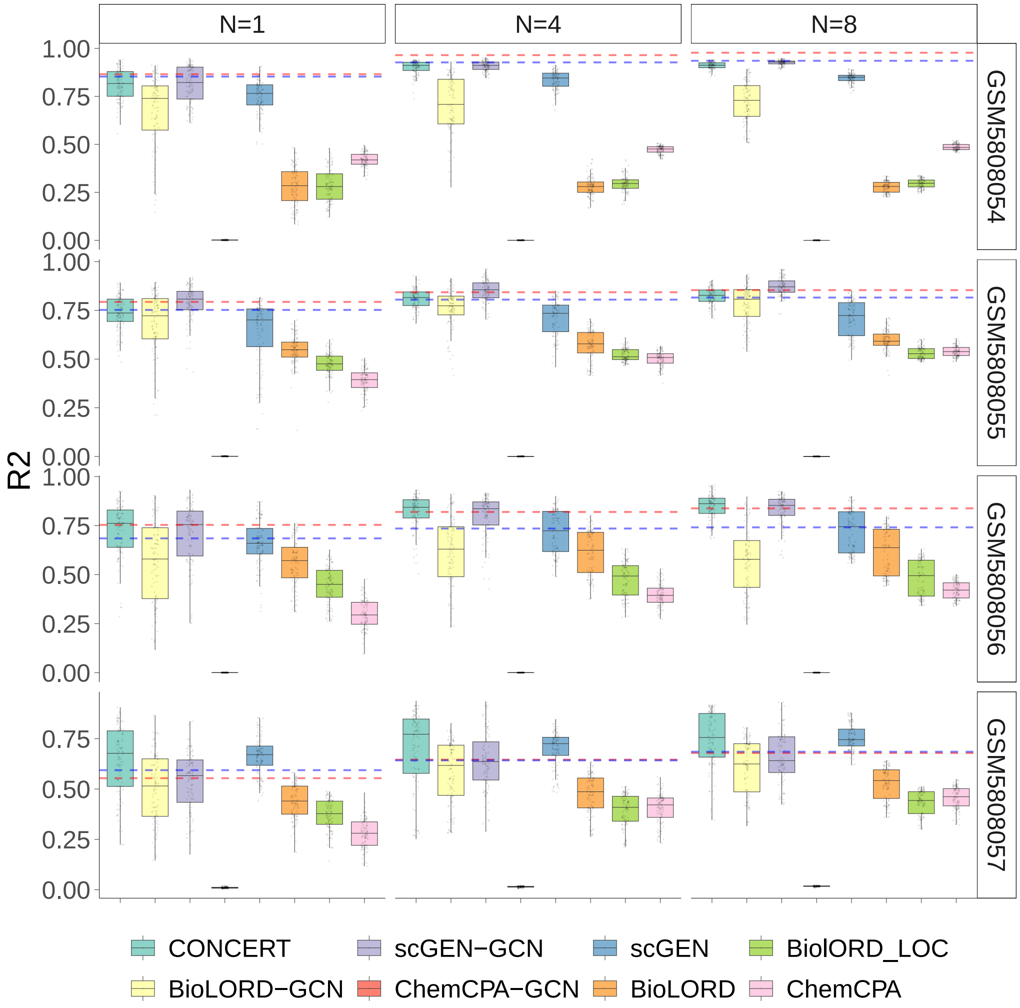

Supplementary Fig. 28: Benchmarking experiments of cross-niche patch perturbation task on Perturb-map datasets evaluated by R^2. KNN-SP and KNN-GEX are shown by red and blue dotted line, respectively. Higher value indicates better performance. The central line inside the box represents the median, while the top and bottom edges correspond to the first (Q1) and third (Q3) quartiles. The whiskers extend to the smallest and largest values within 1.5 times the interquartile range (IQR) from the quartiles. P-values from the comparisons between CONCERT and competing methods are provided in supplementary table 16.

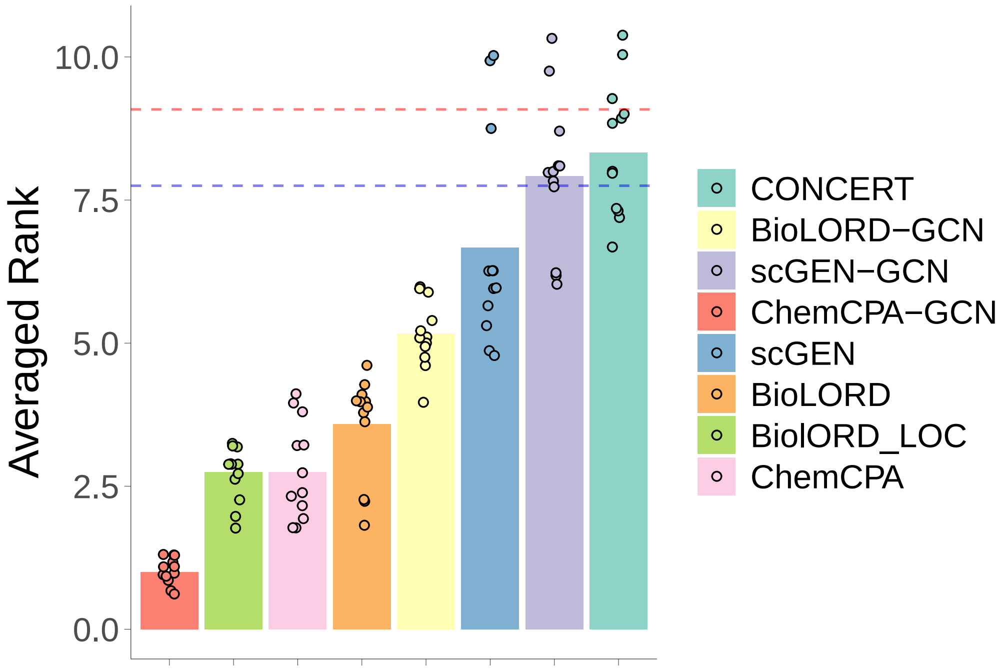

Supplementary Fig. 29: Average ranks of the cross-niche patch perturbation task on Perturb-map datasets evaluated by R^2. KNN-SP and KNN-GEX are shown by red and blue dotted line, respectively. Higher value indicates better performance.

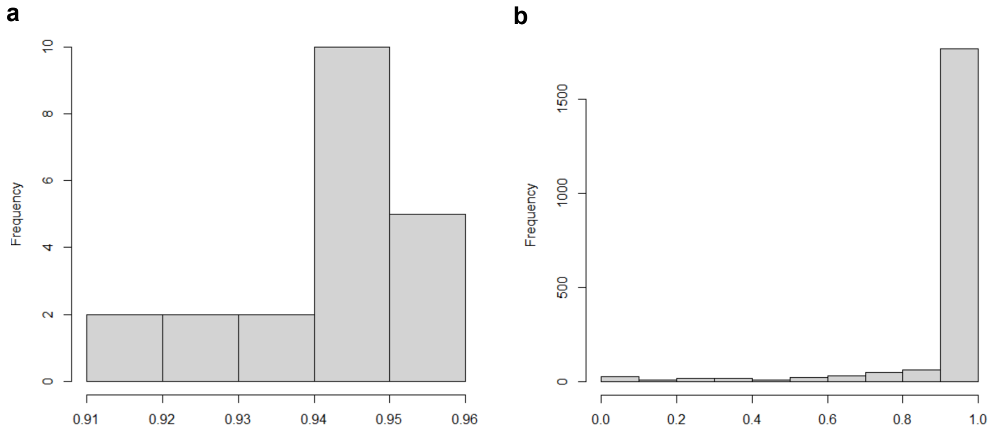

Supplementary Fig. 30: Uncertainty of prediction on within niche task. (a) Distribution of the ratios of genes that fall within the 95% confidence interval (CI) of predictions in the unpaired ground truth spots. (b) Distribution of the ratios of cells that fall within the 95% CI of predictions for the top 2000 highly variable genes.

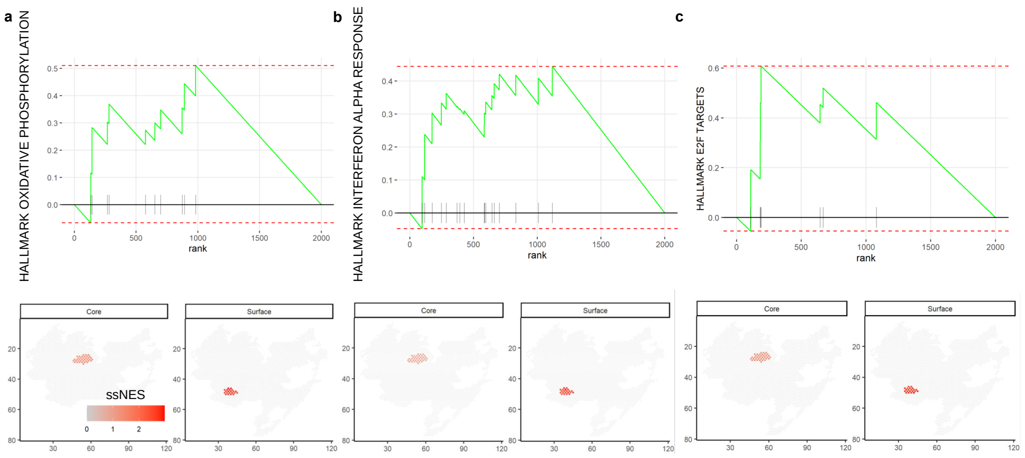

Supplementary Fig. 31: Geneset enrichment plots and single-sample GSEA plots for the results from the case study based on the Perturb-map data. We plot the pathways that are enriched in tumor surface: (a) OXIDATIVE PHOSPHORYLATION; (b) INTERFERON ALPHA RESPONSE; and (c) E2F targets.

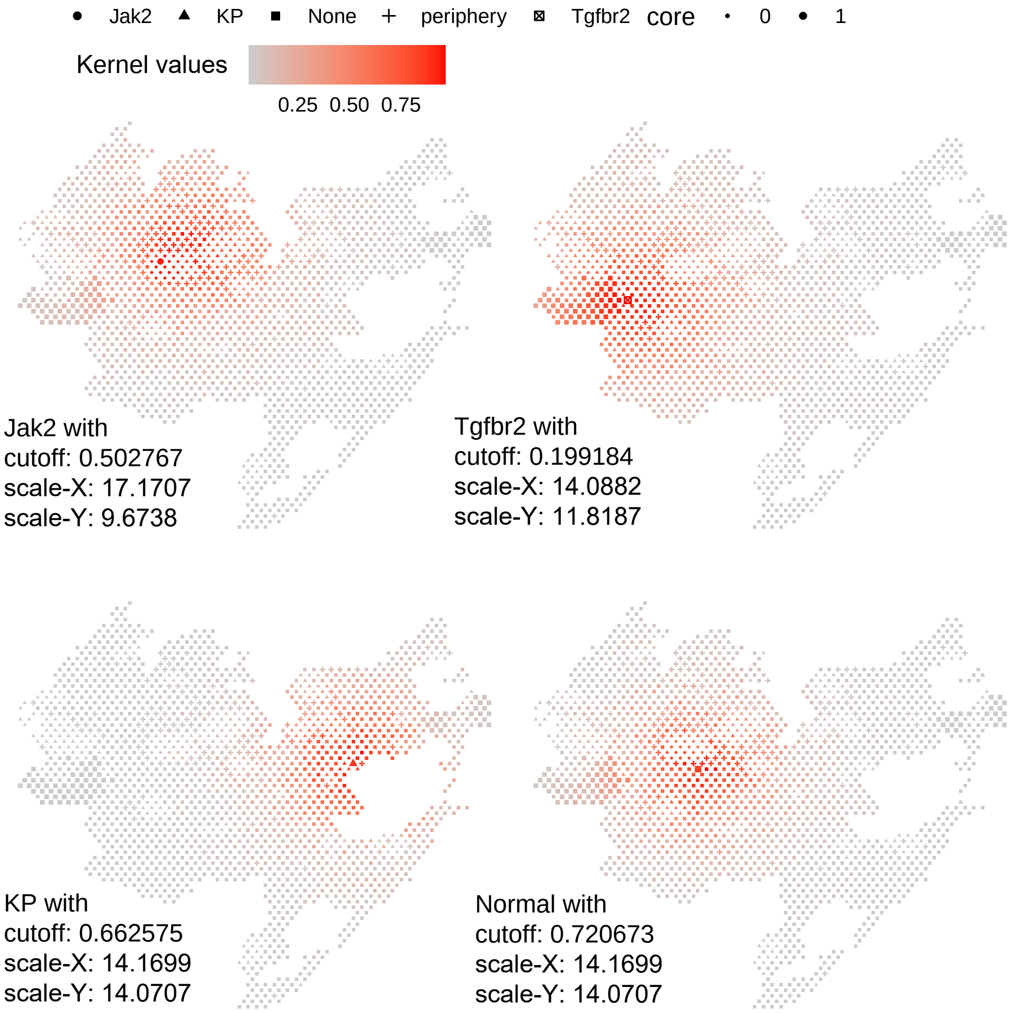

Supplementary Fig. 32: Learned kernel values in CONCERT for four example spots in different types from Perturb-map data slide GSM5808054.

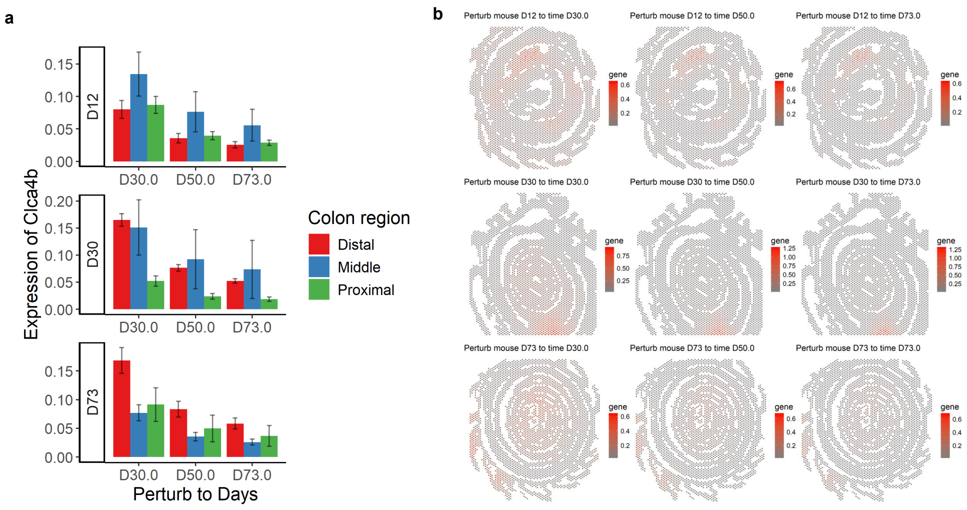

Supplementary Fig. 33: Imputed data of mouse gut for the missing time-points visualized by gene Clca4b. Normalized gene expression (NEX) values are shown in both bar (a) and dot plots (b). CONCERT can counterfactually predict the post-perturbation Clca4b expression of each mouse over the entire timeline, and in-paint the NEX in the missing time points (day 50 here). We observed that the NEX of maker gene Clca4b are consistently deceased in each colon region for each mouse over time

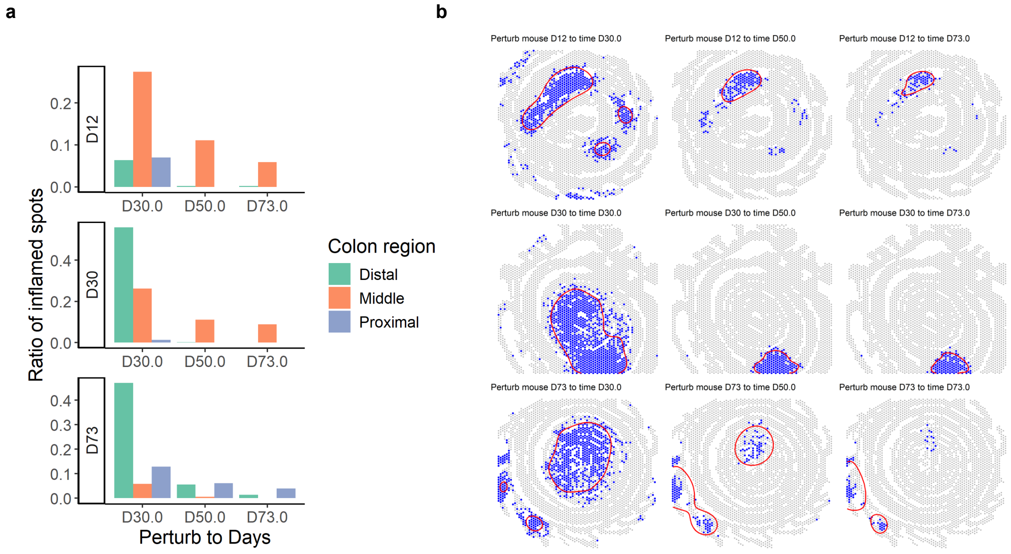

Supplementary Fig. 34: Positive and negative inflamed spots identified by using cutoff quantile 0.85 of marker gene Clca4b. We illustrate that the number of positive cells for the markers in various region are consistently deceased for each mouse over time. These trends are clearly shown in bar (a) and dot plots (b).

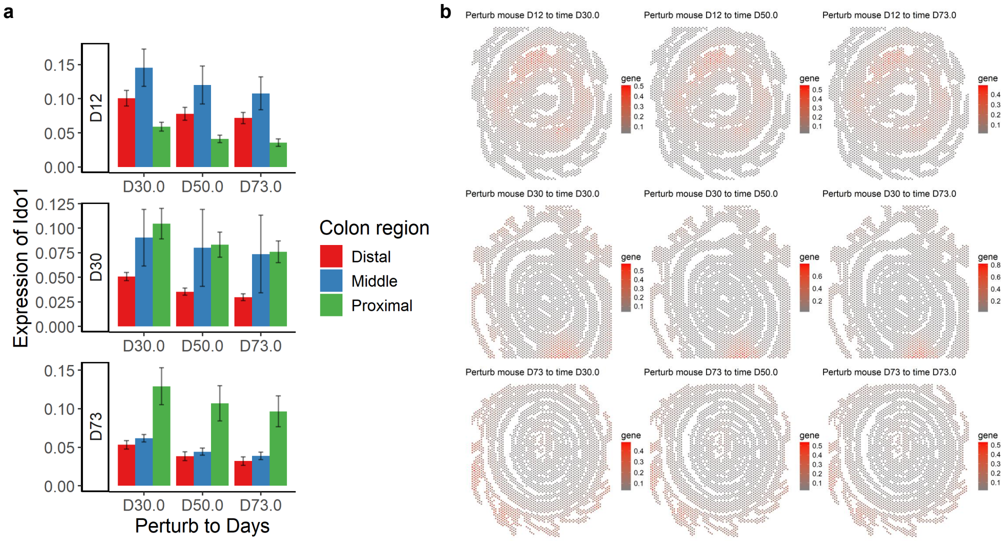

Supplementary Fig. 35: Imputed data of mouse gut for the missing time-points visualized by gene Ido1. Normalized gene expression (NEX) values are shown in both bar (a) and dot plots (b). CONCERT can counterfactually predict the post-perturbation Ido1 expression of each mouse over the entire timeline, and in-paint the NEX in the missing time points (day 50 here). We observed that the NEX of maker gene Ido1 are consistently deceased in each colon region for each mouse over time

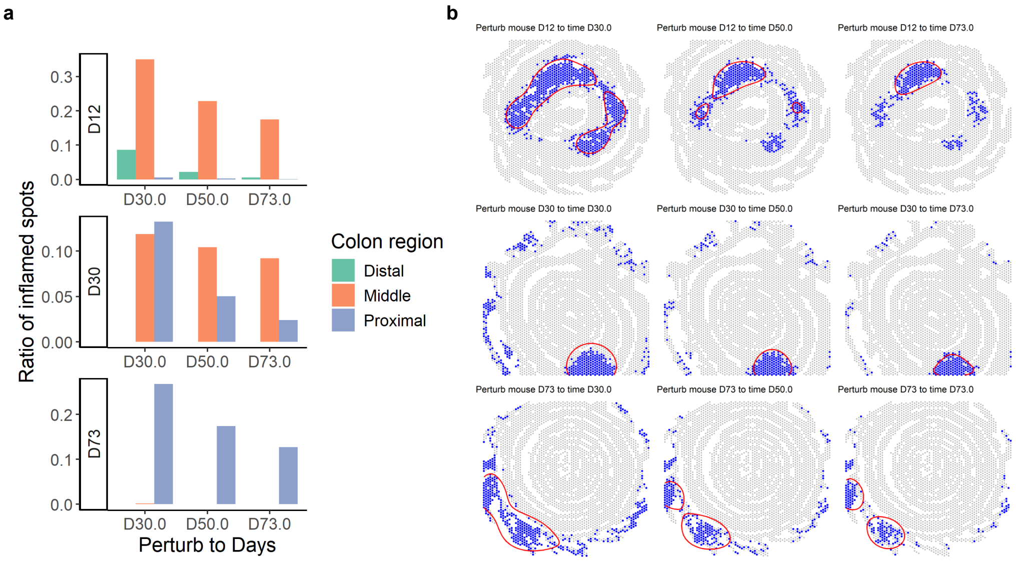

Supplementary Fig. 36: Positive and negative inflamed spots identified by using cutoff quantile 0.85 of marker gene Ido1. We illustrate that the number of positive cells for the markers in various region are consistently deceased for each mouse over time. These trends are clearly shown in bar (a) and dot plots (b).

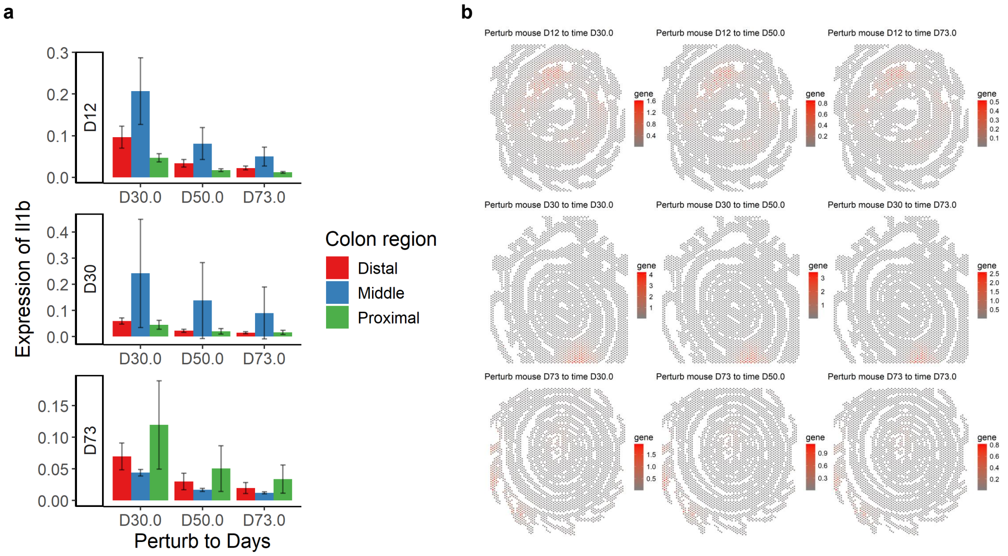

Supplementary Fig. 37: Imputed data of mouse gut for the missing time-points visualized by gene Il1b. Normalized gene expression (NEX) values are shown in both bar (a) and dot plots (b). CONCERT can counterfactually predict the post-perturbation Il1b expression of each mouse over the entire timeline, and in-paint the NEX in the missing time points (day 50 here). We observed that the NEX of maker gene Il1b are consistently deceased in each colon region for each mouse over time

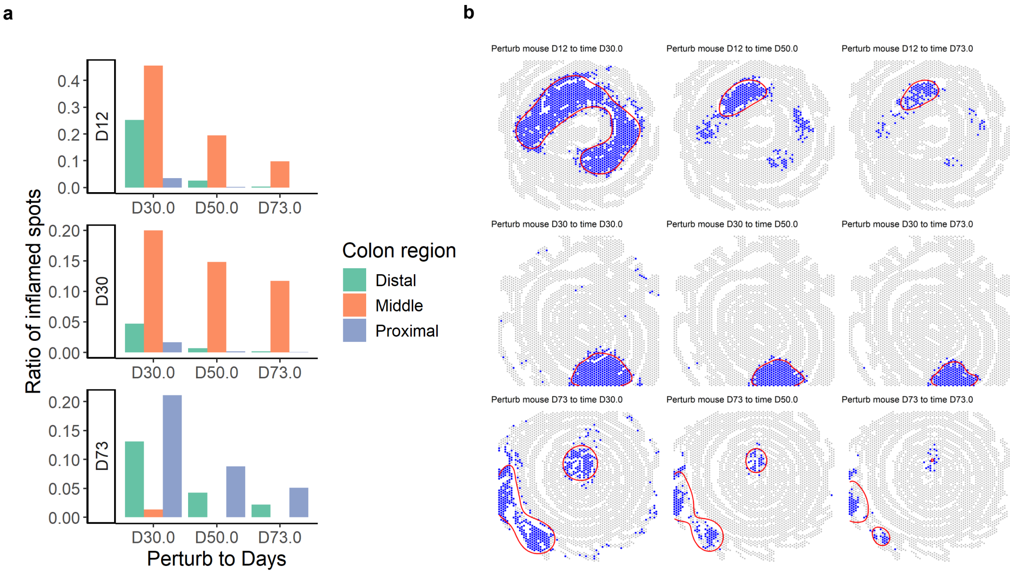

Supplementary Fig. 38: Positive and negative inflamed spots identified by using cutoff quantile 0.85 of marker gene Il1b. We illustrate that the number of positive cells for the markers in various region are consistently deceased for each mouse over time. These trends are clearly shown in bar (a) and dot plots (b).

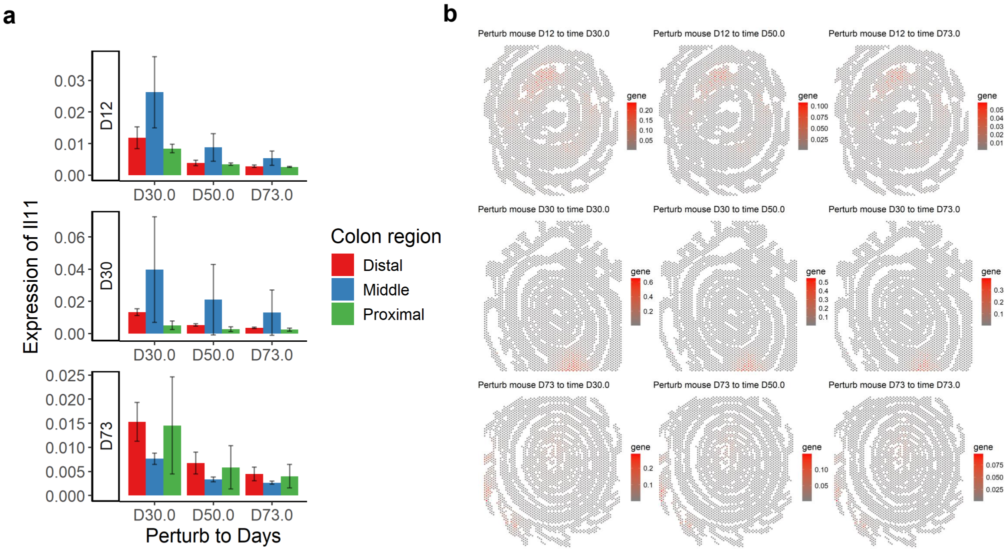

Supplementary Fig. 39: Imputed data of mouse gut for the missing time-points visualized by gene Il11. Normalized gene expression (NEX) values are shown in both bar (a) and dot plots (b). CONCERT can counterfactually predict the post-perturbation Il11 expression of each mouse over the entire timeline, and in-paint the NEX in the missing time points (day 50 here). We observed that the NEX of maker gene Il11 are consistently deceased in each colon region for each mouse over time.

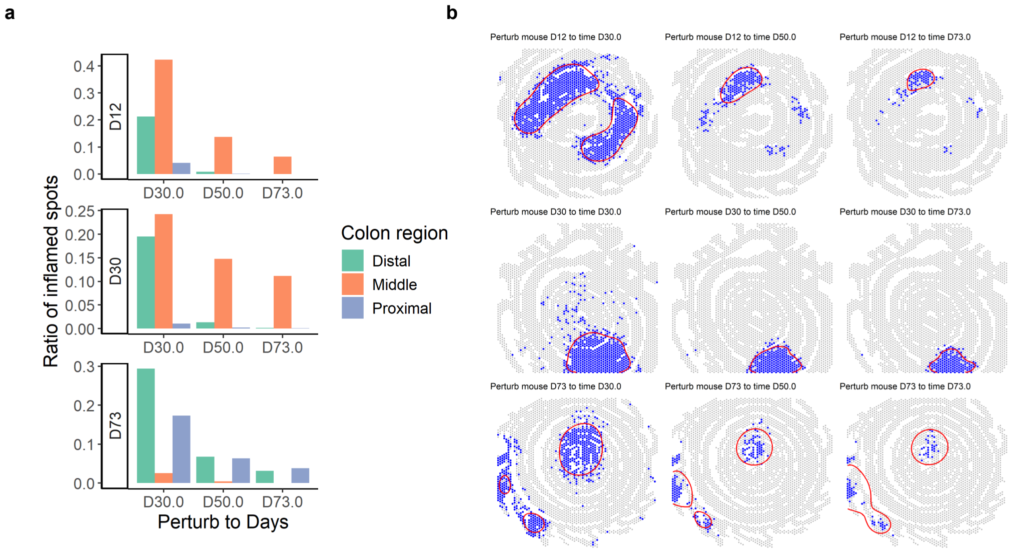

Supplementary Fig. 40: Positive and negative inflamed spots identified by using cutoff quantile 0.85 of marker gene Il11. We illustrate that the number of positive cells for the markers in various region are consistently deceased for each mouse over time. These trends are clearly shown in bar (a) and dot plots (b).

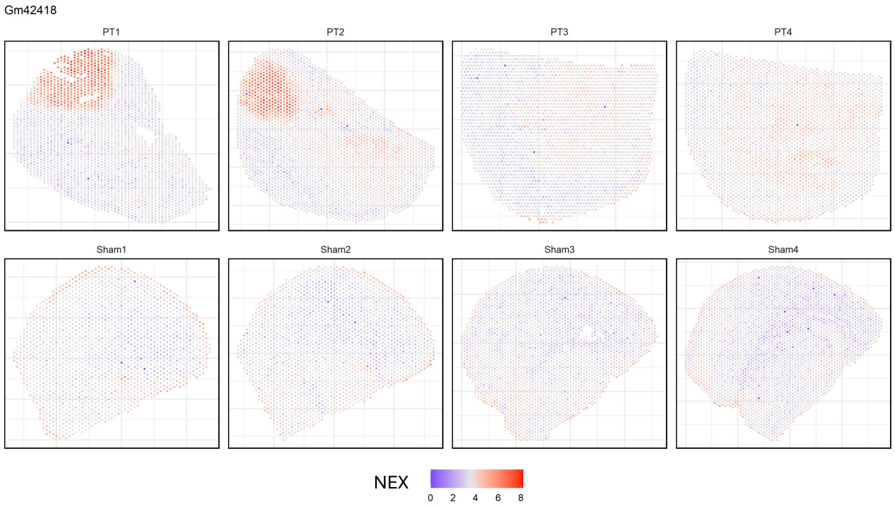

Supplementary Fig. 41: Normalized expression of the marker gene Gm42418 indicating the core ischemic region.

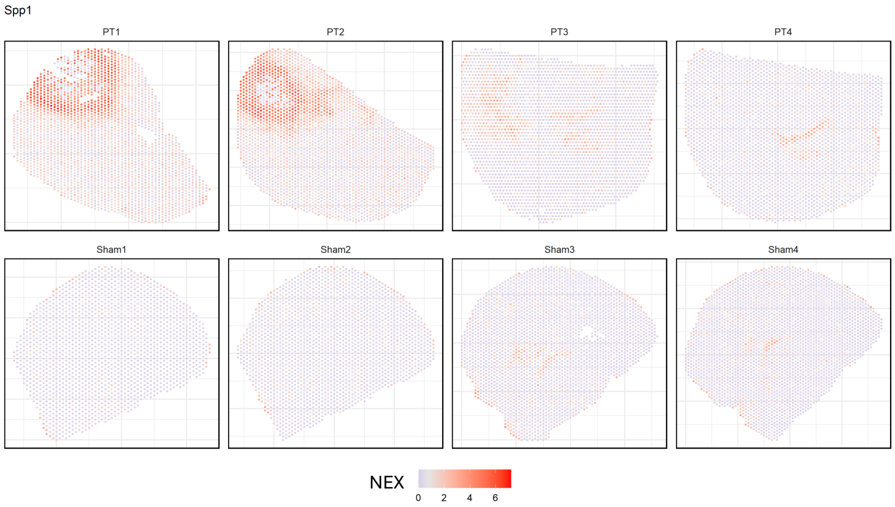

Supplementary Fig. 42: Normalized expression of the marker gene Supp1 indicating the periphery ischemic region.

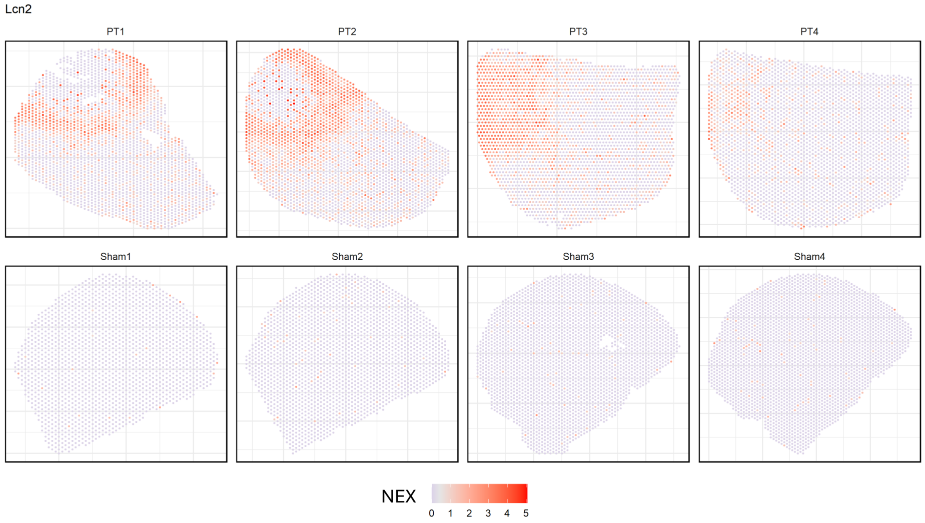

Supplementary Fig. 43: Normalized expression of the marker gene Lnc2 indicating the periphery ischemic region.

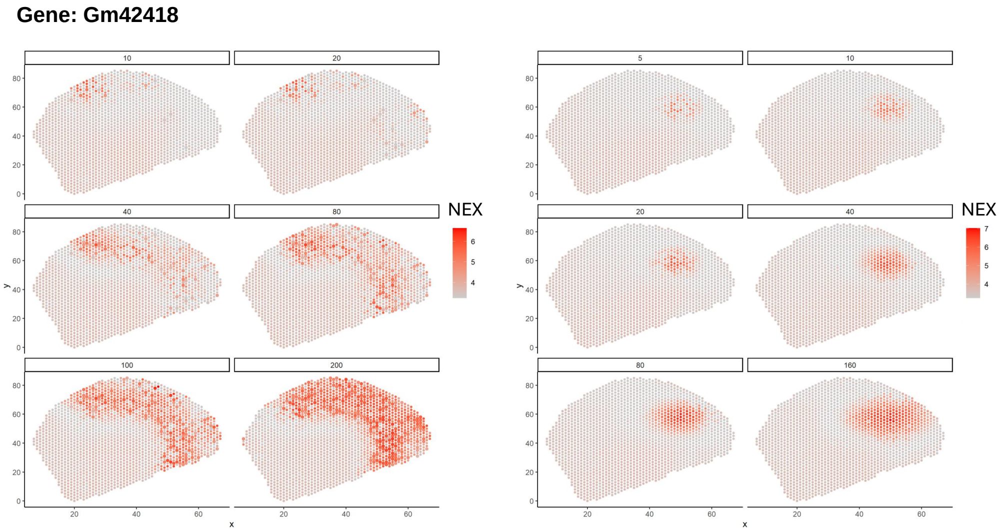

Supplementary Fig. 44: Predicted post-perturbation expression of marker gene Gm42418 to *in-silico* PT perturbation. Spots are sampled random over slide (left) and in the same region to form a patch (right). The numbers of sampled spots are set to 10, 20, 40, 80, 100, and 200

Spp1

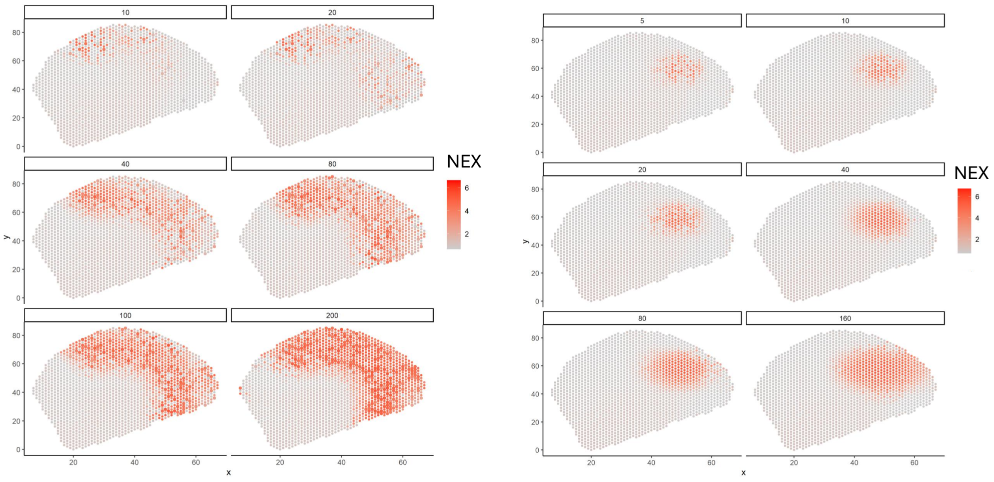

Supplementary Fig. 45: Predicted post-perturbation expression of marker gene Supp1 to *in-silico* PT perturbation. Spots are sampled random over slide (left) and in the same region to form a patch (right). The numbers of sampled spots are set to 10, 20, 40, 80, 100, and 200

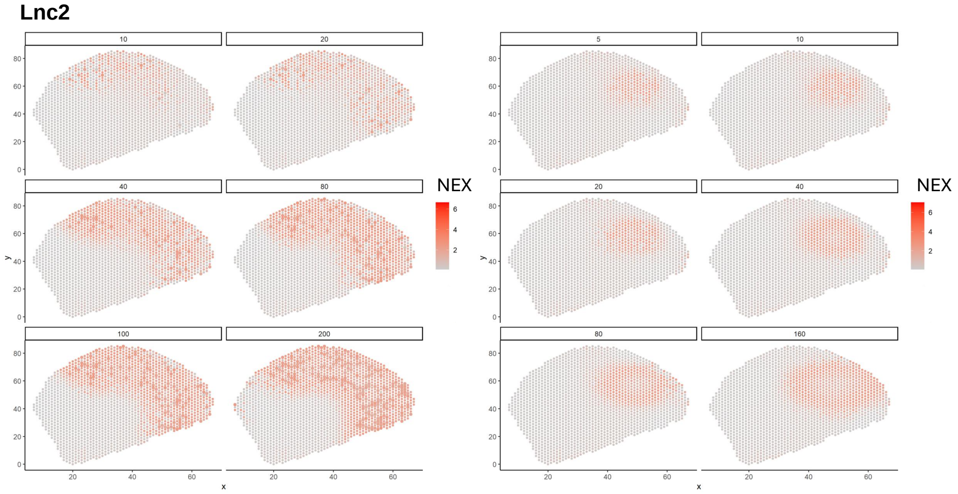

Supplementary Fig. 46: Predicted post-perturbation expression of marker gene Lnc2 to *in-silico* PT perturbation. Spots are sampled randomly over the slide (left) and in the same region to form a patch (right). The numbers of sampled spots are set to 10, 20, 40, 80, 100, and 200.

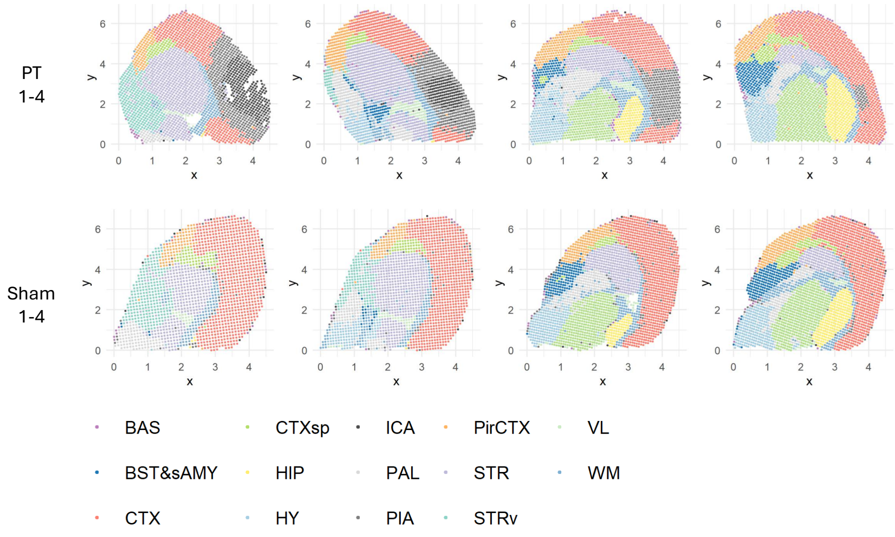

Supplementary Fig. 47: Aligned PT and Sham slides in mouse stroke datasets for building 3D coordinate system.

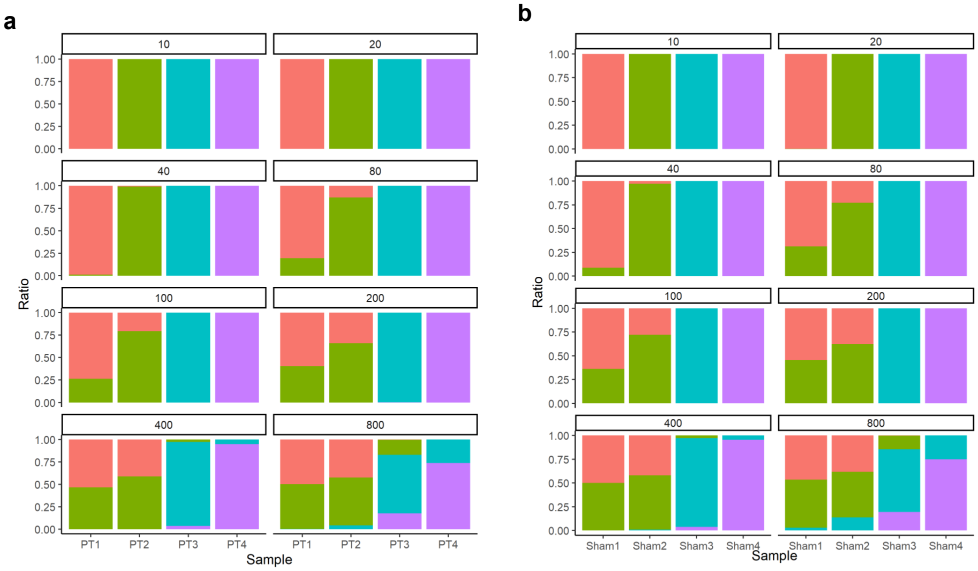

Supplementary Fig. 48: Neighbors within and across samples using 3D coordinates in the mouse stroke dataset (PT: a; sham: b).

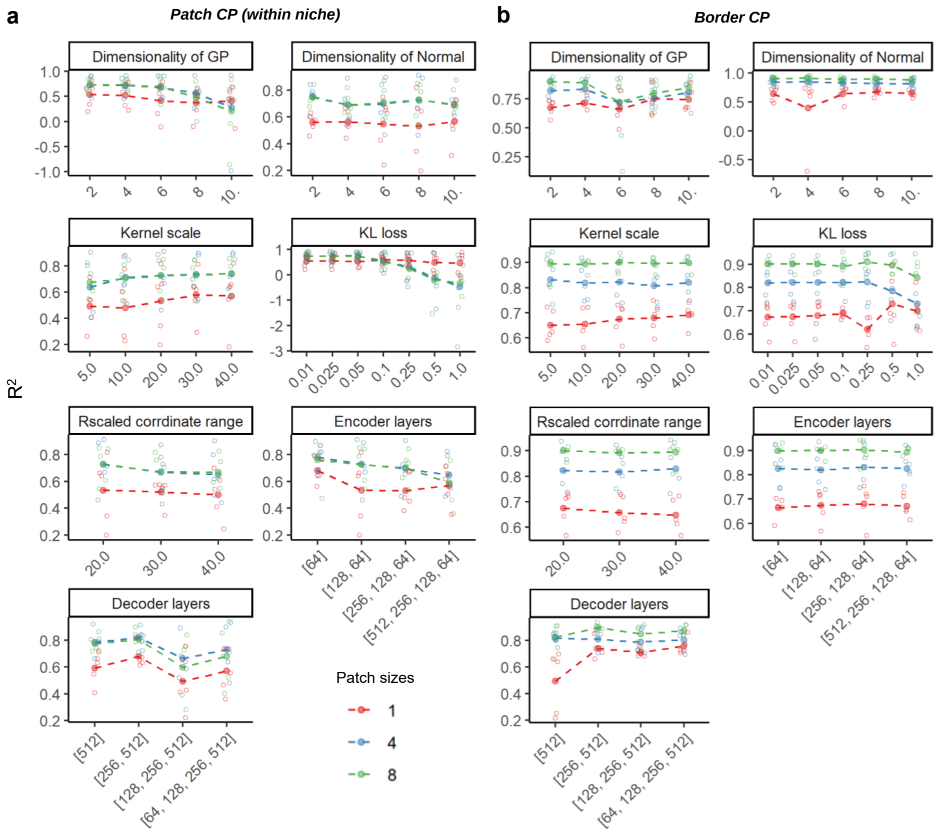

Supplementary Fig. 49: Sensitivity analyses of CONCERT. We ran sensitivity analyses of CONCERT on both patch (a) and border (b) tasks.

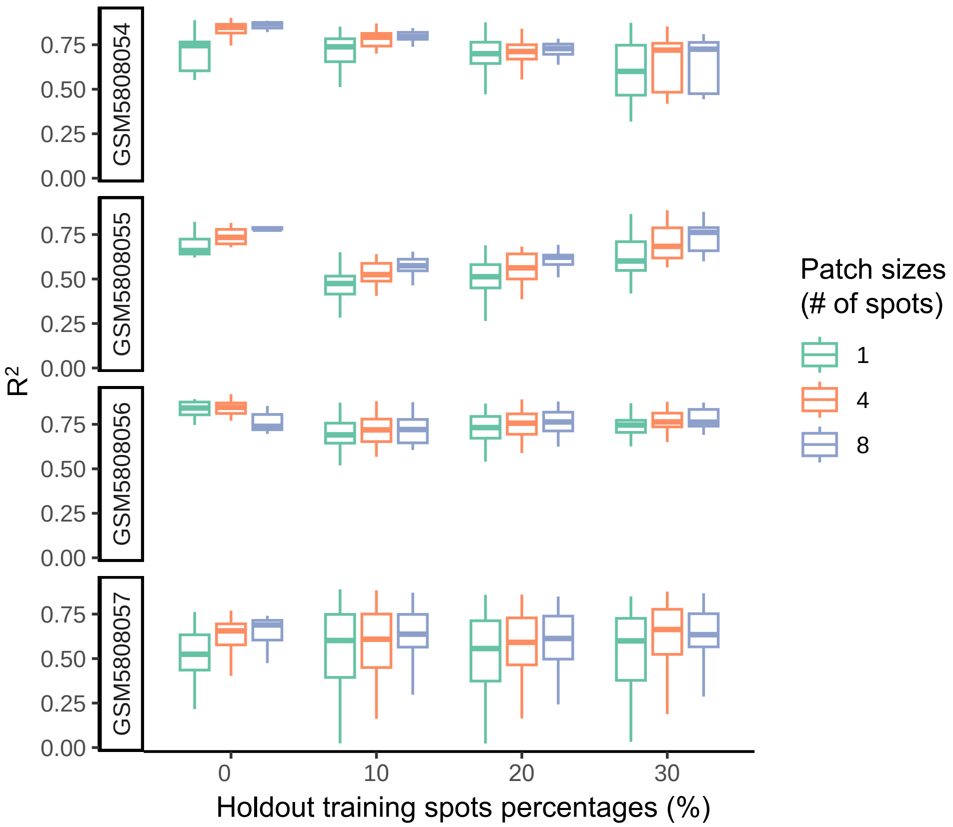

Supplementary Fig. 50: Sensitivity of CONCERT to training size. We subsampled the training data by removing 10%, 20%, and 30% of spots and evaluated performance on the within-niche patch CP task for GSM5808054, GSM5808055, GSM5808056, and GSM5808057. Performance is reported as E-distance.
